# A Framework for Integrating Qualitative and Quantitative Data in Knowledge, Attitude, and Practice Studies: A Case Study of Pesticide Usage in Eastern Uganda

**DOI:** 10.3389/fpubh.2017.00318

**Published:** 2017-12-08

**Authors:** James Muleme, Clovice Kankya, John C. Ssempebwa, Stella Mazeri, Adrian Muwonge

**Affiliations:** ^1^Department of Biosecurity, Ecosystems and Veterinary Public Health, School of Biosecurity, Biotechnical and Laboratory Sciences, Makerere University, Kampala, Uganda; ^2^Department of Disease Control and Environmental Health, School of Public Health, Makerere University, Kampala, Uganda; ^3^Division of Genetics and Genomics, The Roslin Institute, Royal (Dick) School of Veterinary Studies, University of Edinburgh, Easter Bush, Midlothian, United Kingdom

**Keywords:** knowledge, attitudes, practices, quantitative and qualitative, methodology

## Abstract

Knowledge, attitude, and practice (KAP) studies guide the implementation of public health interventions (PHIs), and they are important tools for political persuasion. The design and implementation of PHIs assumes a linear KAP relationship, i.e., an awareness campaign results in the desirable societal behavioral change. However, there is no robust framework for testing this relationship before and after PHIs. Here, we use qualitative and quantitative data on pesticide usage to test this linear relationship, identify associated context specific factors as well as assemble a framework that could be used to guide and evaluate PHIs. We used data from a cross-sectional mixed methods study on pesticide usage. Quantitative data were collected using a structured questionnaire from 167 households representing 1,002 individuals. Qualitative data were collected from key informants and focus group discussions. Quantitative and qualitative data analysis was done in R 3.2.0 as well as qualitative thematic analysis, respectively. Our framework shows that a KAP linear relationship only existed for households with a low knowledge score, suggesting that an awareness campaign would only be effective for ~37% of the households. Context specific socioeconomic factors explain why this relationship does not hold for households with high knowledge scores. These findings are essential for developing targeted cost-effective and sustainable interventions on pesticide usage and other PHIs with context specific modifications.

## Introduction

The success of public health interventions (PHIs) in resource-limited settings critically depends on our understanding of the socio-anthropological and economic aspects of the context in which these interventions are implemented (1–3). However, PHIs have historically followed a top down approach, consistently ignoring the social, political, and cultural context which perpetuates “a-one-size-fits-all” mentality (4–6). The impact that comes with a departure from such an approach is well demonstrated in the recent Ebola outbreak in West Africa (3). By taking into account the behavioral and social norms within the affected communities, public health officials were able to limit the scale of the outbreak (3). Such context-specific public health information is gathered through knowledge, attitude, and practice (KAP) studies (1). These studies are usually aimed at identifying indicators that can inform and improve the development and implementation of PHIs (7). Here, we used knowledge on pesticide usage and practices associated with poisoning to provide public health context. This is because the World Health Organization and United Nations Environment Program estimate that ~1.5 million agricultural workers are affected by pesticides poisoning every year globally (8–10). Indeed 200,000 of these lose their lives or survive with adverse health effects (10, 11). This figure is likely a gross under estimation considering that most developing countries in Africa and Asia have poor reporting systems. Anecdotal reports suggest that pesticide poisoning in Africa alone could be double the global estimates (12, 13). Countries like Uganda are experiencing a rapid population growth characterized by rapid urbanization (13, 14). This comes with profound changes in food security especially in crop production which is now more than ever dependent on the ubiquitous use of pesticides (13). Such demographic shifts inherently alter societal beliefs and practices; therefore, it is important to consider these changes when designing PHIs (14). KAP surveys are the most widely used studies for uncovering societal context specific dynamics in public health (1, 7). These studies are popular because (a) they are easy to design, (b) the data output is quantifiable, (c) the interpretation is robust if both qualitative and quantitative aspects are used, and (d) their utility is generalizable for context specific problems (15). However, it is important to note that there are remarkably few KAP studies that combine both qualitative and quantitative data (1).

Knowledge, attitude, and practice studies fundamentally assume a linear association between knowledge, attitude, and behavioral change (16). Therefore PHIs informed by KAP data target knowledge through awareness campaigns with the expectation that this would promote good attitudes and ultimately lead to the desirable positive change in behavior (2). This rarely tested axiom is the basis upon which billions of tax payer’s money is spent on PHIs. This is also possibly the source of historical and contemporary criticism against the tool. For example, in 1977, Werner highlighted the inconsistencies in the relationship between attitudes and practices on family planning which made it difficult to evaluate the usefulness of the intervention at the time (7). These sentiments are still being echoed in literature reviews today (1). Despite the criticism, the tool is still popular and used with varied consideration for integrating qualitative and quantitative data (2).

**Figure 1:**
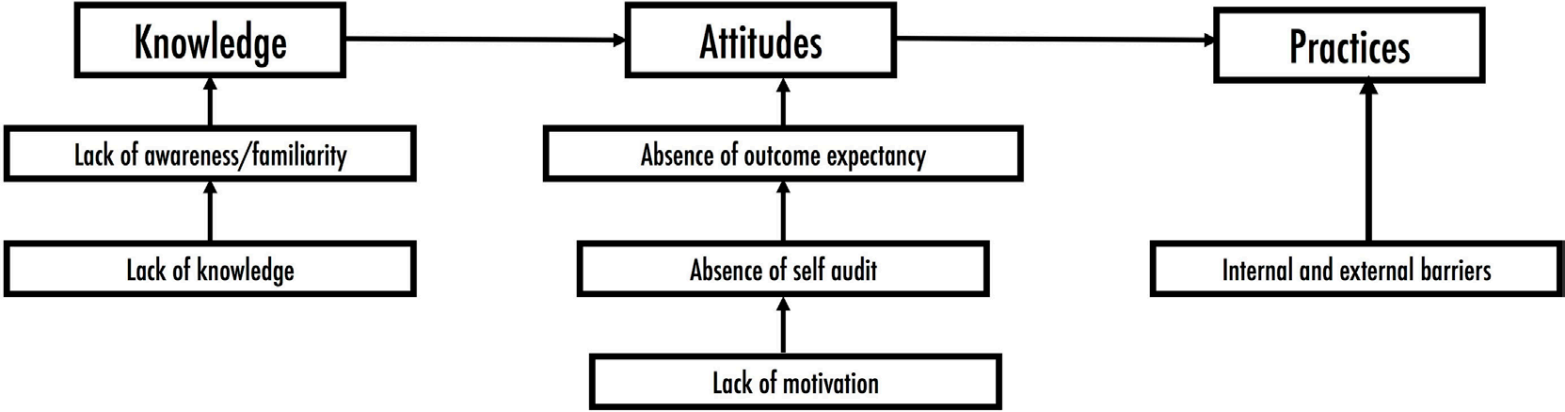
A conceptual framework of the knowledge, attitude, and practice on pesticides usage. It shows the model through which practices can be changed by adjusting attitudes through knowledge/awareness.

To fundamentally bring about a sustainable social and behavioral change regarding exposure and health side effects of pesticides, the World Health Organization recommends that we develop evidence-based interventions (17). The KAP conceptual framework in Figure 1 has been exploited for this purpose in this study. It is based on the assumed linear relationship between knowledge, attitudes, and practices. The lack of knowledge assessed as function of awareness or familiarity of health-related aspects is assumed to influence motivation for self-audit on public health-related aspects. It is, thus, expected that such a scenario would be characterized by attitudes centered on a lack of specific expectations and only be reversed by creating awareness on the public health issue in question. This intervention is then expected to produce the desirable actions and is the fundamental basis of most PHIs (2).

Here, we used qualitative and quantitative data collected in a cross-sectional study on pesticide usage to (a) test the linear relationship between KAPs; (b) identify context specific factors explaining the relationship between KAP; and (c) combine the analytical tools used in a and b to develop a framework for integrating quantitative and qualitative data for KAP studies.

## Materials and Methods

### Study Site

**Figure 2:**
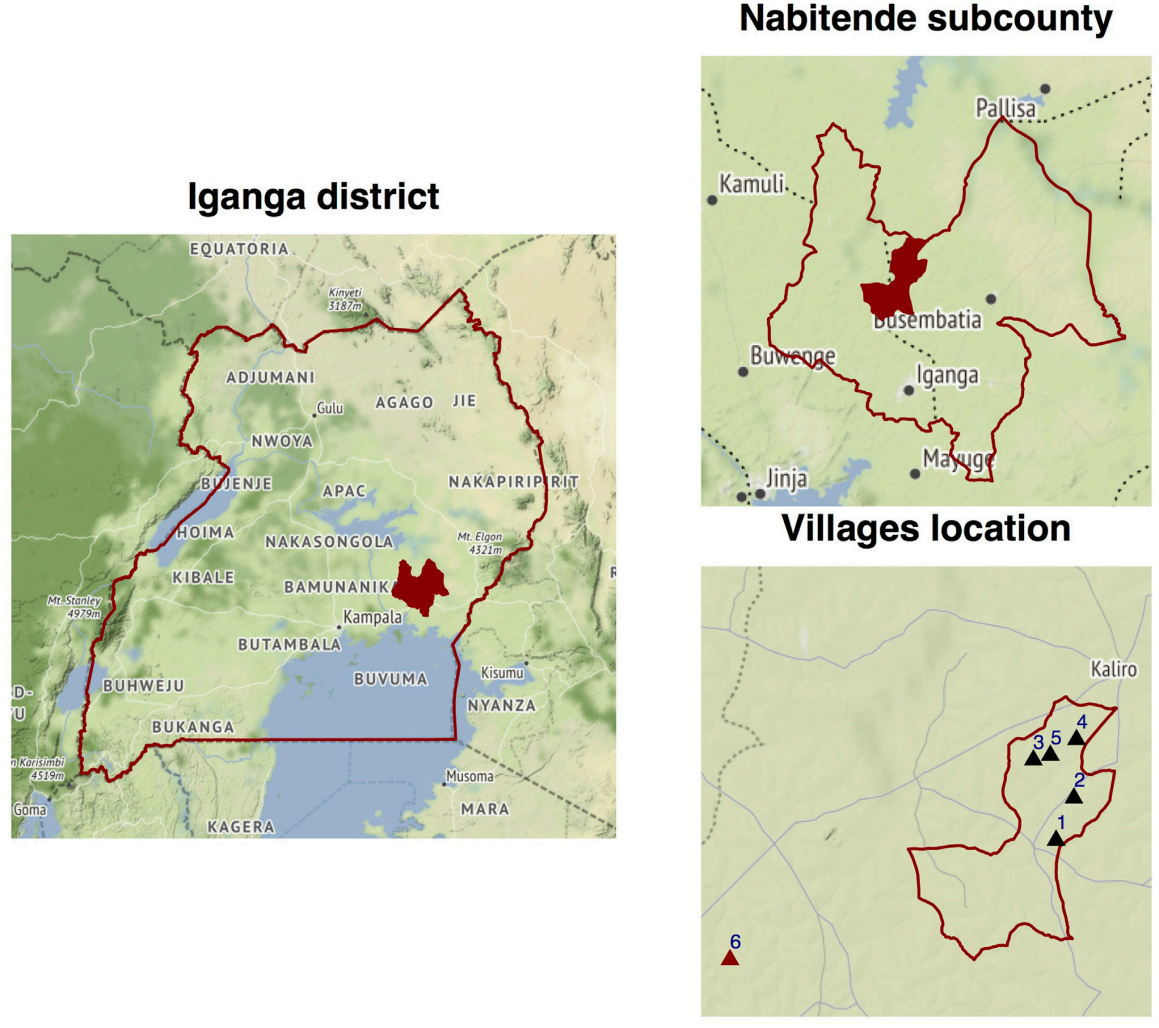
The highlighted shape is the map of Iganga district within eastern Uganda. In the next caption, within Iganga district is the map of Nabitende Sub County the study site. The triangles in the third caption are the villages visited during this study, i.e., 1–5 is Bukaigo, Buvule, Namusisi, Kasambika 1, and Buwerempe. 6 is Namungalwe where the questionnaire was pretested. The map is generated using open source shape files from http://openstreetmapdata.com with ggplot and ggmap in R.

The cross-sectional study was carried out in Nabitende Sub County found in Kigulu North County, Iganga district, located in the Eastern region of Uganda (Figure 2). Nabitende is predominantly a rural agricultural area subdivided into six parishes. It is home to 28,170 people which represents ~6% of the district population. The population in this sub county is housed in ~5,225 households with on average six occupants (18). The residents of this sub county have historically grown cash crops like maize, sugar, tea, and coffee using conventional methods with a limited use of pesticides (19), the *status quo* on pesticide usage has, however, changed in the recent past (13).

### Data Source Triangulation

**Figure 3:**
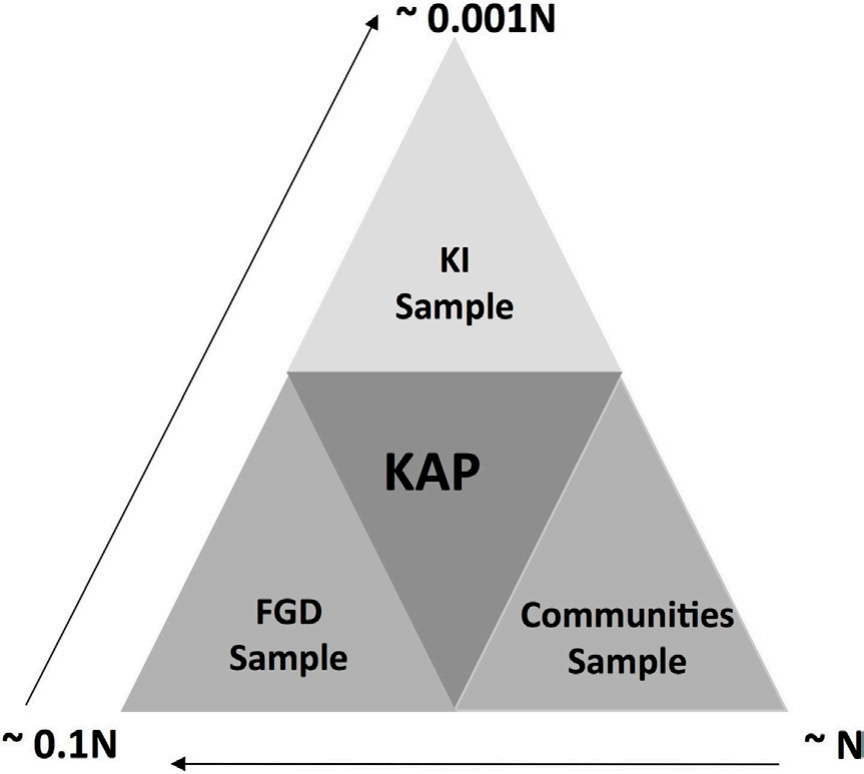
Data triangulation approach used in this study. *N* represents the sample size used for questionnaire administration. 10 and 1% of *N* was used of the focus group discussions (FDG) and key informant interviews (KI).

The triangulation of data shown in Figure 3 allowed for the utilization of information from different sources providing depth and breadth to our understanding of the KAP relationship (20). Here, we used data from three sources: (1) quantitative data at household level collected using a structured questionnaire; (2) data collected through focus group discussions (FGDs) involving a selection of health workers, farmers, and local leaders from the sub county, and (3) data from key informants collected predominantly from pesticide sellers through open-ended interviews.

### Sample Size Estimation

This study was conducted between November 2014 and February 2015 employing quantitative and qualitative data collection methodologies. With full written consent, we administered questionnaires to 167 participants each from a household which represents ~4 and 20% of the households in the sub county and parish, respectively. The parishes chosen in this study represent the highest concentration of farmers, therefore, ideal for investigating agricultural pesticide usage (19). The sample size estimation procedure adopted here uses household as the unit of investigation as previously documented (21).

$$N=\left[Z_{\alpha /2}^{2}\times P\frac{\left(1-P\right)}{D}\times \text{design effect}\right]$$

where *N* is the required number of households and $Z_{\alpha /2}^{2}$represents the statistical level of significance. *P* is the prevalence of pesticide usage in this sub county, here we assumed this parameter to be 50% due to lack of precise documentation of usage. The SE *D* was taken to be 8% as had been done elsewhere (21). We allowed for a non-response ~10% of the sample size. With an average household occupancy of six (18), the views collected in this study are representative of an average of ~1,002 individuals in this sub county.

### Defining Context

#### Knowledge

In this study, knowledge assesses the extent to which individuals from a household know public health concepts regarding pesticide usage. This was not limited to knowledge of biochemical facts but indeed included local knowledge and beliefs, knowledge of available pesticide distribution systems, awareness of rights to access, chemical risk documentation, and awareness of risk to pesticide poisoning and most of all literacy.

#### Attitudes

The attitude attribute characterizes an individual’s feelings, inclinations and indeed those of other household members with regards to pesticide usage. These were characterized as *negative (bad)* or *positive (good)* in relation to the scientifically documented risks of pesticide poisoning and environmental contamination.

#### Practices

The practice attribute documents the actions related to pesticide usage, right from purchasing, usage, to disposal of the pesticide receptacles. These were also characterized as *proper* or *improper* in relation to the scientifically documented risks of pesticide poisoning and environmental contamination.

### Designing the Survey Questionnaire

An interviewer-led structured questionnaire was designed and administered exclusively to households to collect quantitative data. A total of 53 questions were included in this questionnaire categorized as follows; 8 covering the participant’s social and demographic characteristics, 11 questions on knowledge assessment, 12 questions were used to characterize attitudes, and 22 questions for profiling practices toward pesticide usage (S1 in Supplementary Material). We also used an observation checklist, which included 10 questions that were used to verify certain practices like storage and disposal of pesticide and packaging material.

#### Validating the Survey Questionnaire

This was done partly to determine if some important dichotomous variables could be pre-recoded and provided as options for responses. We also wanted to get an expert opinion on the completeness and effectiveness of the questions; therefore, the questionnaire was reviewed by two experts in pesticides usage at the Ministry of Agriculture Animal Industry and Fisheries; Uganda. The field level validation was done through a pre-testing process, where 40 questionnaires were pretested in a nearby sub county of Namungalwe (see Figure 2). This was aimed at establishing an approximate time needed to administer the questionnaire without causing distress to the participants as well as obtain feedback on the appropriateness of the content and accuracy of the translations. The responses obtained here were analyzed using a principal component analysis (PCA) to test the contribution of each question to KAP attributes using component correlation coefficients (see methodology Quantitative data section). This measure was used to identify irrelevant questions, which were then removed. So, the final questionnaire included 48 questions categorized as follows: 9 covering the participant’s social and demographic characteristics, 11 questions on knowledge assessment, 11 questions were used to characterize attitudes, and 17 questions for profiling practices toward pesticide usage (S1 in Supplementary Material).

#### Social Desirability and Acquiescence Biases

A pretest of the data collection tools was run to ensure that acquiescence bias was limited. The pretest allowed for a balanced questionnaire, i.e., positively and negatively keyed questions of the content. On the other hand, social desirability bias was limited by ensuring that all respondents answer the questionnaires independently without external influence. We ensured that the interviews were conducted in a secluded place and established good rapport with the interviewee.

### Qualitative Data Collection

Qualitative data were collected from key informant interviews and FGDs as described in detail below (Qualitative evaluation checklist, see S7 in Supplementary Material).

#### Key Informant Interviews

The selection of key informants was based on their participation and influence on the pesticide supply chain in this area. We, therefore, conducted seven key informant interviews with pesticide sellers in the major townships of Nabitende Sub County and Iganga district highlighted by the participants as being their sources of pesticides. The key informant interviews were conducted by trained members of our team. This ensured a high response rate given that these interviews required scheduling meetings in advance. A 45-min open-ended interview structured around KAP was conducted with aid of an audio recorder and later transcribed into themes. Here, we anticipated bias to arise from using only one sector as our key informants; however, attempts were made to mitigate this by building rapport and asking questions that would provide insights into their networks along the pesticide supply chain.

#### Focus Group Discussions

The FGDs were modulated by two members of the team and were designed to generate a diverse range of opinions on the attributes within the 1-h meeting. The groups included farmers, community health workers, and local leaders. Farmers included in FGD meetings did not take part in the questionnaire-based data collection. Six FGDs were conducted in total, two in Kasambika and Bukaigo villages, respectively. In addition, an FDG was conducted at the health center (HC II) and village local council meeting, respectively. Each FGD comprised of eight participants (female and male in a ratio ~1:1) with an age range of 34–45. The former was aimed at ensuring representativeness. Each FGD line of discussion was stopped at saturation point, i.e., a point when no new information was generated. The FGD discussions too were structured around knowledge, attitudes, and practices. The farmer participation in these FDGs was through local leader nomination. We minimized participation bias by limiting the amount of information known prior to the discussions but clearly communicating the intention of the meeting. We also assured the participants at the beginning of each meeting that there are no wrong or right answers and that what was important for us was their opinion on this matter. The discussions were conducted in the local language and later translated and transcribed in English.

### Data Management

#### Collating Quantitative Data

These data were collected and collated from the questionnaire and an observation checklist. The data were then coded and entered into EPI-DATA software version 3.02 and then exported to SPSS version 19.0 and R version 3.1.2 for analysis.

### Quantitative Data

#### Developing the KAP Metrics

We used knowledge-based questions to develop a metric that ranked a respondent’s knowledge. We chose 11 questions (S1 in Supplementary Material) to which the respondent had to answer YES or NO. Yes indicating their acknowledgment of knowing an aspect about pesticide usage and NO indicating the opposite. The knowledge metric is the proportion of YES response out of the 11 knowledge-based questions for each individual. This metric was converted into a binary outcome, i.e., adequate knowledge (knowledge metric >50%) and inadequate knowledge (knowledge metric <50%) for use in logistic regression modeling.

To evaluate the contribution of each question to this metrics, all the questions were used in a PCA. The weight of each question was taken to be the correlation coefficient with the component that explained the most variation (S2 in Supplementary Material).

### Analysis of KAP Using PCA

A PCA was used to explore correlation–structure free relationships between KAP with regards to pesticide usage. Similar to the Knowledge metric, an attitude and practices ranking metrics was developed from 11 and 17 attitude and practices questions, respectively (S2 in Supplementary Material). Here, YES and NO responses reflected positive/negative attitudes and good/bad practices. These three metrics were then used to examine the linear relationship between KAP by (a) visualizing the variability of the 167 data points along three orthogonal lines corresponding to three components; (b) computing and comparing the correlation coefficients between KAP as well as other sociodemographic factors (S2 in Supplementary Material). A positive correlation coefficient indicates a direct relationship between KAP, while a negative correlation coefficient indicates an antagonistic relationship.

Internal consistency and reliability of the factors included in the PCA for linear relationship were tested using Cronbach’s alpha (22, 23) (Table 1).

**Table 1:** Reliability and internal consistency testing of factors used in PCA.

	Reliability if factor is dropped (With Age and Sex)				Reliability if factor is dropped			
Factors	Raw alpha	Std. alpha	95% Confidence interval (CI)	Cronbach’s alpha	Raw alpha	Std. alpha	95% CI	Cronbach’s alpha
Knowledge	0.24	0.36	(−0.084, 0.084)	0.561	0.46	0.72	(−0.038, 0.038)	0.823
Attitude	0.23	0.33	(−0.085, 0.085)		0.42	0.66	(−0.044, 0.044)	
Practice	0.50	0.36	(−0.049, 0.049)		0.87	0.87	(−0.020, 0.044)	
Age	0.65	0.66	(−0.030, 0.030)		NA	NA	NA	
Sex	0.63	0.66	(−0.035, 0.035)		NA	NA	NA	

### Analysis of Associations between Knowledge, Attitudes, and Practices Using Logistic Regression

The linear relationship was further evaluated; first, correlation coefficients for the relationship between KAP were estimated. Thereafter, a multivariable logistic regression model was built to identify which variables could predict an adequate knowledge score. In order to do this, the binary knowledge score was used as an outcome variable. Explanatory variables included each person’s social demographics, as well as responses to the attitudes and practices questions individually. Initially, a univariable analysis was carried out, where the relationship of each variable to the outcome was compared individually and odds ratios, *p*-values, and confidence intervals (CIs) were reported in tables. Variables with a *p*-value <0.25 were then used to develop a logistic regression model. The model was built using the backward selection of explanatory variables until we obtained model stabilization with the lowest Akaike information criterion. Model validation was done using the standard Hosmer Lemeshow test and area under the curve.

### Qualitative Data Analysis

Qualitative data were analyzed using the content thematic approach, which was guided by the Graneheim and Lundman framework (24). We identified study themes and sub-themes following multiple reading of interview and discussion transcripts. Qualitative data were then transcribed into patterns and themes that addressed the objectives of the study and the observations were triangulated onto the rest of the data to add depth.

## Results

### Sociodemographic Characteristics of Respondents in Eastern Uganda

The sociodemographic analysis shows that up to 81.4% (136/167) of the respondents were married with at least two dependents in the household (Table 2). The majority of the respondents were 17–44 years of age and nearly half of them had primary level education as the highest education attainment. The summary analysis also shows that subsistence farming was the most common source of livelihood mostly practiced on family-owned land (Table 2).

**Table 2:** Sociodemographic characteristics of the respondents in eastern Uganda.

Factors	Level	Frequency (*N* = 167)	Percentage (%)
Age	<22	20	6.5
	22–27	28	25.0
	28–31	23	21.4
	32–39	19	
	40–45	29	22.6
	46–53	23	24.4
	>54	25	

Sex	Male	84	50
	Female	84	50

Marital status	Married	136	81.4
	Single	24	14.4
	Widowed	7	4.2

Number of dependents	None	12	7.2
	Less than three	15	9.0
	Three and above	140	83.8

Education level	Never	35	21.0
	Primary	74	44.3
	Secondary	54	32.3
	Tertiary	4	2.4

Farming type	Subsistence	157	94.0
	Commercial	10	6.0

Land ownership	Family	125	74.9
	Relative	11	6.6
	Friend	9	5.4
	Rented	22	13.2

Farming experience	Below 24 years	103	61.7
	25–48 years	55	32.9
	Above 48 years	9	5.4

### Evaluation of the Linear Relationship between KAP Using PCA

**Figure 4:**
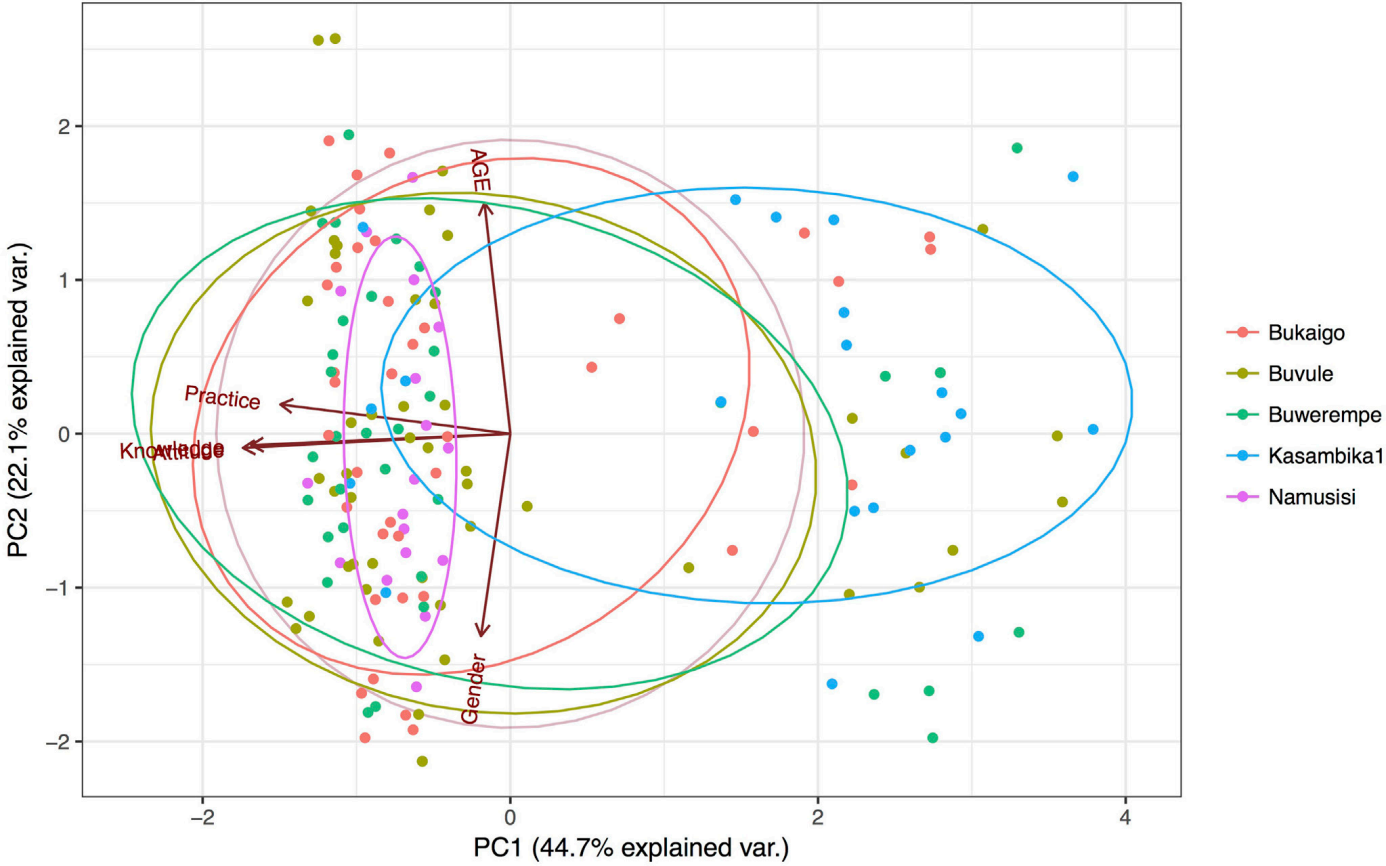
Principal component analysis of knowledge, attitudes, and practices toward pesticide usage in eastern Uganda. Each colored point plotted corresponds to a respondent’s village of residence.

The results of the PCA analysis between knowledge, attitudes, and practices of pesticide usages are shown in Figure 4 and Table 4. The findings show a linear relationship between KAPs in the first component, which explains 44.7% of the variation. It is important to note that practice is not as close as attitude is to knowledge, the same relationship is revealed by correlation coefficients in Tables 4 and 5. The second component explains 22.1% of the variation most of which is influenced by the respondent’s gender and years of experience in farming. Note that the latter and former are in opposition to each other in this component (Figure 4). The third component explains 18.5% of the variation in this dataset, here too the respondent’s gender and years of experience in farming are the most influential. The fourth and fifth component explain 14.4% of the variation, here all but knowledge exhibiting the same influence in both components. We also explored the driver of the variation observed in the first principal component and in this regard findings in S2 in Supplementary Material suggest that knowledge was the main driver behind this variation. We, therefore, further tested the validity of the linear relationship at the two discernible levels of knowledge, i.e., high and low, >50 and <50% score on the knowledge scores, respectively. It is noteworthy that 63 and 37% of the farmer scored high and low on knowledge, respectively. The results in S2 and S3 in Supplementary Material revealed that the linear relationship only held for individuals with a low score (>50%).

Furthermore, the evaluation for reliability and internal consistency was highest (Cronbach’s alpha = 0.82) when gender and age were dropped; these were, however, retained in the PCA analysis for context purposes.

### Analysis of Associations between Knowledge, Attitudes, and Practices

The parametric KAP linear relationship evaluation is shown in Table 5. When the evaluation was done on the full dataset, i.e., without splitting the data by knowledge dichotomy, the knowledge and attitude metrics were strongly correlated (Correlation coefficient = 0.76, *p* = 2.2e−16). The same was not true for the knowledge and practice metric. On the other hand, when data were split by the knowledge dichotomy, the linear relationship holds for all the attributes, i.e., knowledge, attribute, and practice metric (correlation coefficient = 0.61, 0.66, and 0.69, respectively) in the low knowledge group. There was a weak positive correlation between the knowledge and attitude metric for the high knowledge category, but the results also reveal a negative correlation between the knowledge-practice and attitude-practice metric, respectively (Table 5).

#### Knowledge and Sociodemographic Characteristics

**Figure 5:**
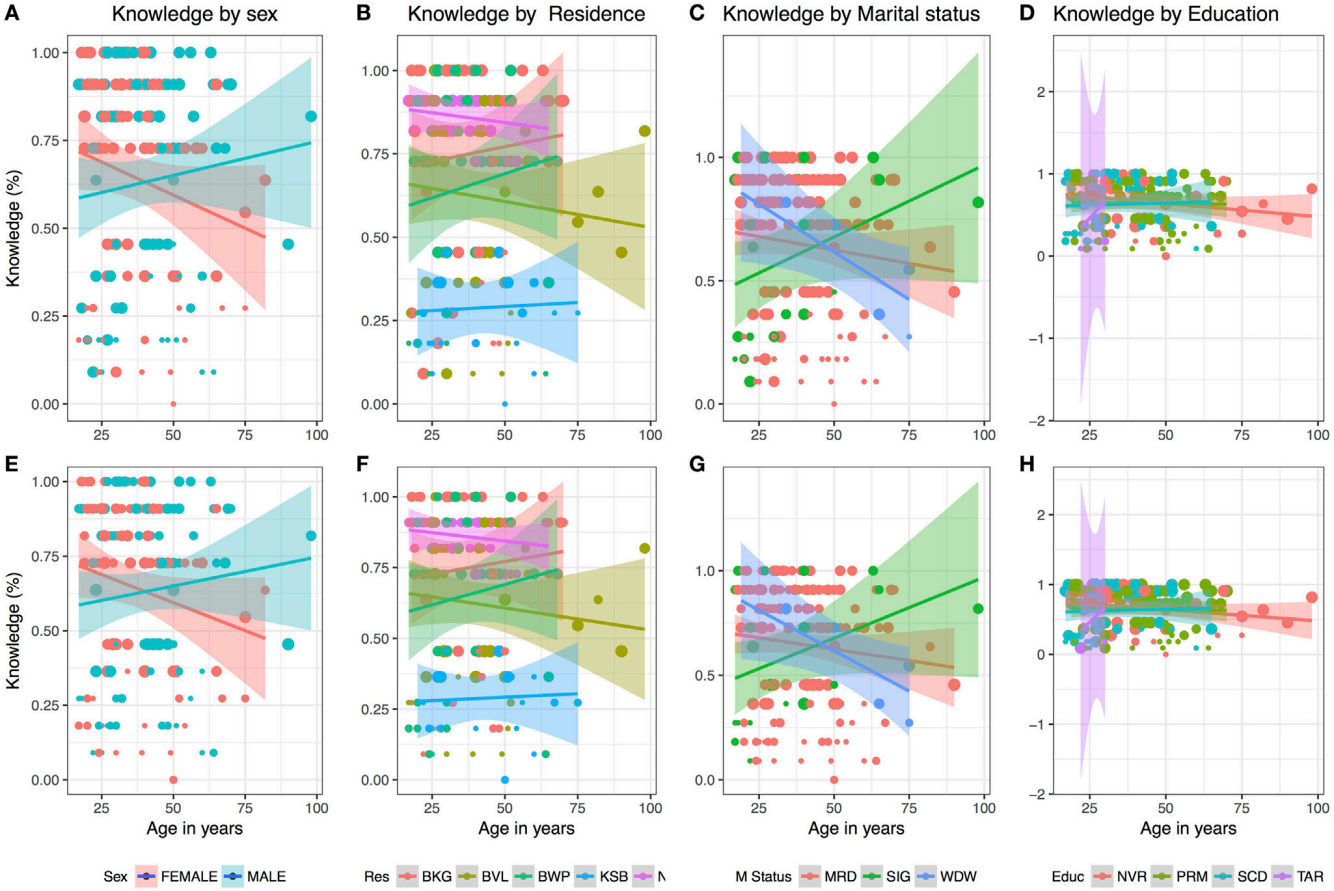
Variation of the Knowledge metric by age of the respondents in years when a sociodemographic characteristic is taken into account. The line whose color corresponds to sociodemographic characteristic is a linear regression line while size of the points **(A–H)** reflects the score on the attitude and practice metric, respectively. These scores ranged between 0.12 and 0.875 and 0.33 and 0.77 on the attitude and practice scale, respectively. Res, residence; where BKG, BVL, BWP, KSB, and NMS represent villages Bukaigo, Buvule, Buwerempe, Kasambika 1, and Namusisi, respectively, M status, marital status, where MRD, SIG, and WDW represent married, single, and widowed, respectively, Educ, education level where NVR, PRM, SCD, and TAR represent never had and education, primary, secondary, and tertiary, respectively.

To investigate the relationship between sociodemographic factors and knowledge, we first represented this graphically by plotting the knowledge metric against age split by different sociodemographic characteristic as shown in Figure 5. We observe an increase in the percentage score on the knowledge metric with age among male respondents while the opposite relationship appears true for female respondents (Figure 5). The parish of residence also appears to be associated with the score on the knowledge metric. For example; the knowledge score was inversely related to age in Buvule and Namusisi, while the opposite is true for the rest of the villages. Although there were few respondents with tertiary education, the scores on the knowledge score increased with age of respondent in this category. A univariable analysis of these relationships is shown in Table 3.

**Table 3:** Show the univariable relationship between knowledge and respondents’ attitudes and practices toward pesticide usage.

Category	Questions	Response	Odds ratio (95% confidence interval)	*p*-Value
Attitudes	Do you think it is important to observe weather conditions when before spraying?	Yes	Ref	
		No	1.04 (−1.08, 3.16)	0.96
	If yes, which of following is the least conducive weather for spraying?	Windy	Ref	
		Rainy	1.21 (−0.56, 2.98)	0.74
		Sunny	3.57 (1.77, 5.37)	0.03
		Not sure	1.71 (−0.58, 4.00)	0.51
	Do you think mixing of pesticides is very important?	Yes	Ref	
		No	0.86 (−0.57, 2.29)	0.68
	Do you care that pesticides can enter our bodies?	Yes	Ref	
		No	0.75 (0.25, 1.25)	0.57
	Do you care about the harmful effects of pesticide exposure?	Yes	Ref	
		No	1.48 (−0.10, 3.06)	0.39
	Do you think the following can be potential pesticide exposure symptoms; nausea, vomiting, salivation, skin irritation, and blurred vision?	Yes	Ref	
		No	0.32 (−1.23, 1.87)	0.01
	Do you think you can reduce on the amount of pesticides you use in agriculture without affecting the expected yield?	Yes	Ref	
		No	0.69 (−0.94, 2.32)	0.44
		Don’t know	0.93 (−0.95, 2.81)	0.91

Practices	Where do you buy pesticides?	Iganga	1	
		Nabitende	1.40 (−0.48, 3.28)	0.59
		Kampala	0.42 (−3.52, 4.36)	0.52
	What do you wear when spraying?	Ordinary clothing	1	
		Gloves	0.91 (−0.61, 2.43)	0.82
		Overall	5.78 (2.86, 8.70)	0.10
		Mask	0.43 (−2.10, 2.96)	0.36
		Hat	1.28 (−2.18, 4.74)	0.84
		Long sleeved shirt	0.43 (−2.10, 2.96)	0.36
		Gumboots	1.93 (−1.29, 5.15)	0.58
	What is your average spray time?	<2 h	1	
		2–4 h	0.97 (−0.48, 2.42)	0.93
		>4 h	0.57 (−0.97, 2.11)	0.18
	Do you clean equipment after use?	Yes	1	
		No	2.34 (0.74, 3.94)	0.068
	Where do you do equipment cleaning?	Field	1	
		House	1.40 (−0.11, 2.91)	0.42
		Water source	0.57 (−0.97, 2.11)	0.19
	Where do you store pesticides and equipment?	Inside the house	Ref	
		Outside the house	0.93 (−0.49, 2.35)	0.83
	How are pesticides stored	Left in the original container	1	
		Decanted into another container	0.60 (−0.89, 2.09)	0.204
	Where and how do you dispose the used pesticide containers?	Burn	Ref	
		House	2.13 (0.23, 4.03)	0.24
		Reused	0	0.99
		Latrine	2.50 (0.55, 4.45)	0.17
		Bush	2.45 (0.53, 4.37)	0.17
	How far on average is pesticide storage to water containers for home use	<10 m		
		>10 m	0.50 (−0.92, 1.92)	0.048
	Is the store accessible to children	Accessible	Ref	
		Non-accessible	0.72 (−0.91, 2.35)	0.502

Sociodemographic	Age	<22	Ref	Ref
		22–27	0.713 (0.19–2.40)	0.59
		28–31	0.42 (0.11–1.46)	0.18
		32–39	1.73 (0.40–8.07)	0.46
		40–45	1.02 (0.28–3.55)	0.96
		46–53	0.46 (0.12–1.58)	0.22
		>54	0.82 (0.22–2.89)	0.75
	Gender	Male	Ref	Ref
		Female	1.311 (0.698–2.479)	0.339
	Marital status	Married	Ref	Ref
		Single	0.563 (0.232–1.359)	0.197
		Widowed	1.408 (0.291–10.087)	0.681
	Education level	Never		
		Primary	0.882 (0.363–2.066)	0.777
		Secondary	0.644 (0.256–1.564)	0.338
		Tertiary	0.478 (0.051–4.413)	0.4886
	Land ownership	Family	Ref	Ref
		Relative	0.707 (0.202–2.577)	0.585
		Friend	2.064 (0.474–14.248)	0.378
		Rented	0.766 (0.312–1.9285)	0.563

#### Knowledge and Attitudes

We also explored the univariable relationship between knowledge and attitudes as shown in Table 3. The results show that respondents who thought that sunny days were the least conducive days to spray had higher probability of scoring highly on the knowledge metric odds ratio (OR) = 3.57, 95% CI (1.77–5.37) and *p* = 0.03. On the contrary, respondents who thought that signs and symptoms like nausea, vomiting, salivation, skin irritation, and blurred vision were not linked to pesticide poisoning had lower probability of scoring highly on the knowledge metric OR = 0.32, 95% CI (−1.23 to 1.87), *p* = 0.01 (Table 3).

#### Knowledge and Practices

The relationship between knowledge and practices of respondents regarding pesticide usage are also shown in Table 3. Respondents who kept pesticides at a distance less than 10 m away from a water for household use had a lower probability of scoring high on the knowledge metric compared to those who stored the pesticides at a distance more than 10 m [OR = 0.50, 95% CI (−0.92, 1.92), *p* = 0.048]. Respondents who wore overalls as their personal protective equipment gear had a higher probability of scoring high on the knowledge metric than those who wore their ordinary clothing while spraying [OR = 5.78, 95% CI (2.86–8.70), *p* = 0.10].

#### Logistic Regression Model Results

The final logistic regression model is shown in Table 6. No sociodemographic characteristic variables were retained in the final model. In terms of attitudes, the model showed that respondents who thought that it was least conducive to spray in sunny and dry conditions were three times more likely to have a good knowledge score than those who thought it was unsafe to do so in windy conditions. With regards to practices, the model shows an association between knowledge and pesticide storage practices. Respondents who answered that they stored pesticides at a distance more than 10 m away from a water source were less likely to have a low score on the knowledge metric (Table 6).

### Thematic Qualitative Analysis of KAP

#### Natural Versus Chemical Pesticides

One of the themes that emerged from our FGD and key informant interviews was pesticide categories. Even though there was common agreement on the efficacy of synthetic Versus natural herbal pesticides, i.e., the latter being less efficacious at killing pests than commercially produced chemical pesticides, the majority of the participants in the FGDs expressed their ongoing use of smoked cow dung to repel flies and pests, urine and tea leaves to kill or repel pests as well as snakes. This was in contrast with the views of key informants who argued that it was not worth risking crops with inferior approaches especially in the current times when the weather is unpredictable.

We know that natural pesticides exist, in fact our ancestors used them for thousands of years. But in this day and age we want to be sure of the returns on our hard labor. So we use chemical pesticides because we are sure they will kill all the pests (FGD in Itanda parish).

Farmers have started complaining that unlike in the past when they used natural pesticides, when they reduce the amount of chemical pesticides in the gardens, pests return quickly and the crop yields are also poor (Key Informant discussion in Iganga town).

#### Beliefs on Chemical Potency

Beliefs on potency and potentiation were also central to the discussions; some FGD participants believed that the potency of a chemical pesticide could be potentiated by mixing two or more pesticide types. This contrasted the views held by the key informants most of who believed that mixing pesticides would lead to a reduction in their potency.

I have noticed that when I do not mix these chemicals, the weeds and pest return quickly which means I have to repeatedly do the spraying (FGD in Bukaigo).

#### Awareness of Safety Measures

The end justifies the means; is a sentiment that was commonly held by most FGDs participants. Good crop yield was the most important outcome; in this regard, farmers were keen on what the pesticides did when applied than what the manufacturer safety instruction said. In fact, the majority of the participants said they did not read the Material Safety Data Sheets on the pesticide containers. The Key informants corroborated this, they said that most of the farmers would not have had a chance to read these instructions because they bought pesticides in volumes smaller than what the manufacturer distributes.

The majority of the farmers buy the pesticides in volumes smaller than retail container volumes, so unless I read for them the instructions, there is no chance they will ever know what the manufacturer’s instructions said. You see they buy them in polythene bags or used Soda bottles (Key informants interview in Nabitende).

#### Pesticide Supply Chain and Access

The FGDs also attempted to identify solutions to improve safety awareness among pesticide users. Most of the participants did not think the *status quo* could change if the pesticide supply chain remained solely controlled by profit motivated individuals and organizations.

These pesticide sellers are only interested in money, once you hand it to them and they hand you the pesticide, it is the end of matter. I do not remember a single day any of them bothered to explain the dangers of these chemicals, on the other hand I don’t think I have ever bothered to ask (FGD in Buvule Village).

#### Timing and Frequency of Spraying

We also explored the practices related to spraying and here most of the FGD participants said that the timing of spraying was always dependent on the environment. For example, the weather, especially wind speeds, was important for timing. Although a few argued for sunny days, the majority thought windy days were the most conducive for spraying. They argued that chemicals tended to have a pungent smell, so on windy days the smell could be limited by wind dispersal.

These chemicals have a bad smell. If you do not have a mask and you do not want the smell then it is better to spray during windy days. The disadvantage is that it blows away the pesticide so one uses more but also ends up spraying un-intended crops. The rainy seasons are not good for spraying; it washes off the pesticides, so it is not effective. On the other hand during the dry season the leaves tend to fold, so the chemical may not reach the plant well, (FGD Discussion in Kasambika Parish).

Ultimately the quantity and frequency of spraying by far seemed to depend on available disposable income. On the other hand the type of prevalent weed or pest seems to greatly influence whether or not farmers sprayed.

There is no chance that i will be buying pesticides if there is no food at home, yes even if my fields are infested with ’Kayongo’ (Striga Hermonthica). “Kayongo” is our biggest problem causing a lot of poor crop yields (FGD Discussion in Bukaigo Parish).

#### Spraying and Equipment Management

The key informant discussions also revealed interesting safety concerns surrounding equipment management, for example; it is common for pump nozzles to break or get clogged during spraying. In such cases the majority of the farmers would simply use their mouth to blow through the nozzles in an effort to unclog them.

“Many of the farmers do not own spray pumps, they borrow from neighbors or rent from us. when the nozzles break or get clogged, the farmers want to unblock them before returning the pumps to us for fear of being charged.” They use any method possible including blowing through it by mouth (Key Informants, Iganga Town).

## Discussion

Approximately $134 billion was spent by the global aid industry in 2015, a significant proportion of which went to health and public health activities in developing countries (25, 26). The fund-raising campaigns behind such huge sums of money are underwritten by information collected from KAP studies (1, 3). Likewise, the implementation of such PHIs is critically dependent on KAP generated data in order to account for the socio-anthropological context (1, 2). While KAP studies began as scientific instruments to distil social context, they have become incredibly effective tools for political persuasion (1, 7, 16). As a tool, KAP studies ought to be regularly and robustly evaluated and updated, unfortunately this is not the case. In fact one of the shortfalls of this tool has been the lack of a framework for integrating qualitative and quantitative data (1, 3). In this study, we have used pesticide usage data to test the validity of the assumed linear relationship between KAP. Furthermore, we have identified context specific factors associated with increased knowledge and proposed a framework for integrating quantitative and qualitative data for KAP studies.

### Linear Relationship between KAP

Public health interventions that promote awareness are designed and implemented with an assumption that there is a linear relationship between knowledge, attitude, and behavior (1, 16). The findings from our PCA show this linear relationship between KAPs, moreover the relationship between attitudes and knowledge appears to be much stronger than that with practice (Figure 4; Tables 4 and 5). This empirical linear relationship is very rarely considered or demonstrated (27), indeed most studies have evaluated this relationship with attitude as a proxy for knowledge (28–30). Therefore, our findings support the validity of targeting awareness in PHIs in general (1, 16). However, we also show that this relationship does not hold for all individuals in a community. Here, we show that the linear relationship only held for individuals with a low knowledge score (<50%). Therefore, targeting individuals in this category with awareness campaigns would be expected to produce the desirable behavioral change. This has practical implications with regards to designing, implementing and expected impact of public health strategies, i.e., identifying predictors for a low knowledge score can be used to focus awareness campaigns as a public health strategy on farmers in Kasambika parish. On the other hand, the KAP linear relationship does not hold among individuals with high knowledge scores (>50%) a phenomenon that has been previously documented (28, 31, 32). In this regard, we can assume that an awareness campaign alone would not be successful at causing a behavioral change in most farmers (~63%) predominately residing in Bukaigo and Buwerempe parish. Indeed, this non-linear KAP relationship could explain why certain international PHIs have not been successful (1, 7). Van Doorn and colleagues developed a theoretical and mathematical model for describing this non-linear attitude–behavior relationship (31). They argue that there is a critical knowledge–attitude level beyond which the linearity with practice breaks down, our empirical comparison between attitude and practice metric appears to fit their theoretical model (S2 in Supplementary Material). In economics, this non-linearity has been exploited to segment consumers by attitudes and then targeted them with specific advertisements (31). In this study, it would be analogous to segmenting our communities by whether or not this linear relationship holds and designing suitable PHIs for each of the groups. It should be noted that although the internal consistency and reliability was high (Cronbach’s alpha = 0.82); in order to ensure that this analysis retained context, we maintained Age and Gender in the PCA. This is because these two attributes provide categorical and temporal context to the KAP measurements.

**Table 4:** The Linear relationship between PCA components and the knowledge, attitude, and practice attributes.

	PC1	PC2	PC3	PC4	PC5
Knowledge metric	0.59061	−0.01687	−0.14596	−0.44421	0.65721
Attitude metric	0.61042	0.00129	−0.11252	−0.25045	−0.7423
Practice metric	0.52180	0.05183	0.15289	0.82803	0.12652
Gender	0.03971	0.72863	0.65632	−0.19169	−0.0008
Experience	0.06855	−0.68272	0.71546	−0.13120	−0.0089

**Table 5:** Pearson’s correlation coefficient analysis between knowledge, attitude, and practice attributes.

	Knowledge metric		Attitude metric		Practice metric	
	Corr [95% confidence interval (CI)]	*p*-Value	Corr (95% CI)	*p*-Value	Corr (95% CI)	*p*-Value
**Combined knowledge categories**						
Attitude metric	0.76 (0.69–0.81)	2.2e−16	1	–	0.50 (0.38–0.61)	3.243e−12
Practice metric	0.32 (0.18–0.45)	1.87e−05	0.50 (0.38–0.61)	3.24e−12	1	–

**High knowledge category (>50%)**						
Attitude metric	0.40 (0.23–0.55)	2.01e−05	1	–	−0.64 (−0.74 to 0.52)	1.00e−13
Practice metric	−0.37 (−0.53 to 0.20)	7.37e−05	−0.64 (−0.74 to 0.52)	1.00e−13	1	–

**Low knowledge category (<50%)**						
Attitude metric	0.61 (0.42–0.74)	1.34e−07	1	–	0.69 (0.53–0.80)	4.14e−10
Practice metric	0.66 (0.49–0.78)	4.42e−09	0.69 (0.53–0.80)	4.14e−10	1	–

*Corr is the Pearson’s correlation coefficient*.

**Table 6:** Multivariate logistic regression model of knowledge, attitudes, and practices of pesticide usage in Eastern Uganda.

Category	Question	Response	odds ratio 95% confidence interval	*p*-Value
Attitudes	When do you think it is not safe to spray?	Windy	Ref	
		Rainy	1.08 (0.330, 3.589)	0.889
		Sunny and dry	3.441 (1.014, 12.045)	0.047
		Not sure	1.264 (0.228, 7.425)	0.788
	Do you think the following can be potential pesticide exposure symptoms; nausea, vomiting, headache, skin irritation?	Yes	Ref	
		No	0.574 (0.169, 1.264)	0.142

Practices	Proximity of pesticide storage to water source	<10m—Near	Ref	
		>10m—Far	0.451 (0.205, 0.948)	0.040
	Do you clean or wash your sprayer after use?	Yes	Ref	
		No	2.486 (0.969, 7.106)	0.069

*Area under the curve = 0.703, p = 0.512, Akaike information criterion = 207*.

### Factors Affecting the KAP Axiom

Results from our logistic regression and the qualitative thematic analysis were used to explain the dynamics behind this linear relationship, i.e., the individuals for who the linear relationship held. For example; the model shows that attitudes toward weather and its potential to influence decisions on when to spray had a statistically significant association with a high knowledge score. Individuals who thought that it was least conducive to spray in the dry and sunny periods were ~3 times more likely to have a high score on the knowledge metric when compared to those who thought windy weather was least conducive. This seems to go against the common wisdom and the manufacturer’s recommendations. However, the farmer’s reasoning behind this was captured from our FGD. The individuals who preferred spraying on windy days were not bothered about the safety aspects of spraying pesticides, but rather keen on limiting the pungent smell associated with pesticides. Indeed, the FDG discussions revealed that attitudes against spraying during sunny and dry periods were underpinned by the belief that the folding of leaves would limit the effectiveness of the pesticides. Similar sentiments have been echoed in reports on pesticide usage in Ecuador and in West Africa (33, 34). Ignoring such intricate, context specific, and counterintuitive aspects of this relationship are likely to determine whether or not an intervention will be successful for a particular community.

The qualitative data also revealed that the quantity and frequency of spraying was ultimately dependent on the availability of disposable income in household, this could also explain the non-linear KAP relationship observed among individuals who had a high score on the knowledge metric (S2 in Supplementary Material). The nature of this linear relationship among the two knowledge categories combined with the findings from the thematic analysis indeed suggest presence of a knowledge threshold (31) beyond which the limiting factor to behavioral change becomes socioeconomical. Therefore, one could argue that a PHI that does not include economic empowerment is likely to fail for certain segments of a community.

Practices surrounding pesticide storage were also significantly associated with the level of knowledge. Respondents who stored pesticides far (≥10 m) from water storages were less likely to score poorly on the knowledge metric than those who stored pesticides closer to water storage. It is more than likely that this is an indirect association of a practice with knowledge that reflects more than one pesticide usage characteristic of this community. For example; the key informant interviews revealed that the majority of pesticides are bought in receptacles different from those supplied by manufacturer. This inherently implies that farmers do not have access to safety documentation for the pesticides, which is expected to influence their safety and storage practices. The association between pesticide storage and knowledge has also been documented elsewhere (13, 33, 35). For example; in Palestine, storage of pesticides in secure stores was associated with high knowledge scores by farmers (36), while in Tanzania it was linked to a farmer’s education level (35). The findings in the study also highlight safety concerns associated with spray equipment and ownership. The FGD discussions revealed that farmers have to hire spray equipment, which come with fines if returned damaged. This means farmers attempt all means to repair equipment even if it means blowing through clogged spray nozzles with their mouth in order to avoid fines (13).

### A Framework for Integrating Quantitative and Qualitative KAP Data

**Figure 6:**
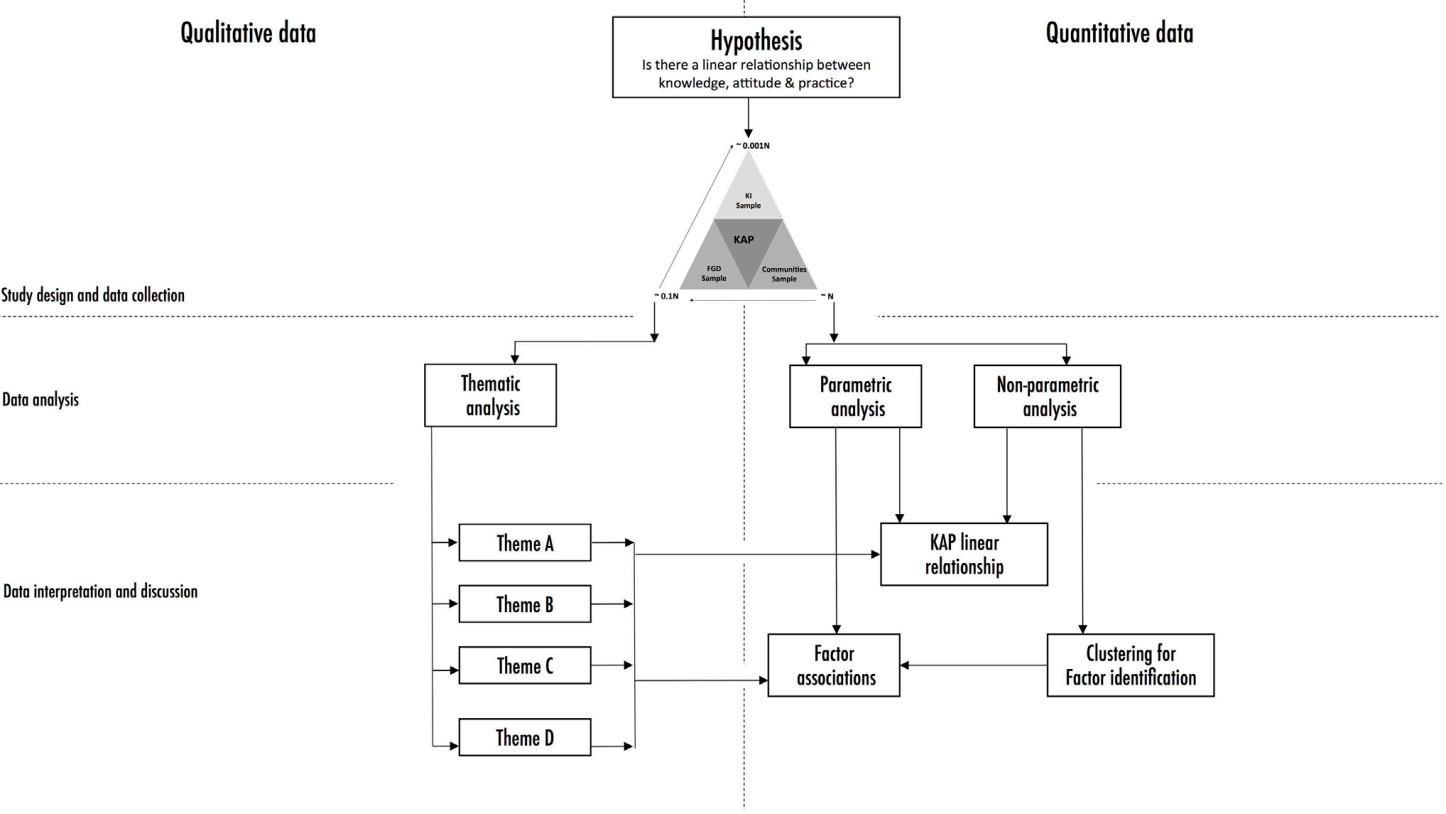
Framework that can be used to map qualitative data onto trends and associations identified by quantitative data. This starts with a fundamental question public health interventions should ask (hypothesis). Is there a direct link between knowledge and practice in the population of interest? The question can be answered by designing a survey using a data triangulation framework to collect both qualitative and quantitative data. These data are collated, managed, and analyzed using thematic as well as standard parametric methods. Identified themes are then used to discuss/explain statistical associations with knowledge to identify which groups to target as well as identify strategies of targeting them.

The fundamental objective of integrating qualitative and quantitative data is to exploit the synergistic aspects in such data in order to add breadth and depth to our understanding of socio-anthropological context. The theoretical framework for this integration was first proposed 17 years ago (37) with a few subsequent modification (38). Our study provides an empirical extension to this framework. Indeed, this framework is built on a hypothesis based on qualitative ideologies (1, 7), but tested using quantitative tools. In a nutshell, the assumption that knowledge influences attitude which in turn modulates actions is a socio-anthropological construct that we have tested as a linear relationship (Figure 6). We have used data triangulation as the foundation for the study design and data collection (20). This not only allowed us to account for qualitative and quantitative data input but also forms the platform for downstream integration (37). The subsequent data analysis uses conventional tools available for both methodologies, i.e., thematic analysis for qualitative data and parametric analysis for quantitative data. The outputs of this step provided us with the points of reference, i.e., identifying qualitative themes and statistical associations that can be used in integrating and explaining outputs from both methodologies.

### Study Limitations

Our selection criterion for participants allowed for adults only due to ethical recommendation. This inherently restricted the breadth of views of pesticide users outside this age bracket. None the less the findings reported here reflect a significant proportion of the farmer dynamics as reported basing on the national demographic report (18). Second, as this was a cross-sectional study, it only provides a snapshot of views which reality could change in time and space. Furthermore, given that PHI follow a minimum of three contact points with communities, i.e., introduction, implementation, and evaluation, a longitudinal study with a minimum of three sampling time points would have been the ideal design. This, however, was not possible because of limited resources. However, the approaches used are still valid for future three contact points KAP studies.

### Relevance to Public Health

Billions of dollars are spent on PHIs informed by KAP studies every year in developing countries. This money can effectively be spent if we can tailor intervention to specific communities with a degree of certainty on their potential impact. Here, we have empirically demonstrated the linear relationship between KAP using data on pesticide usage at household level. Furthermore, we show that the level of knowledge determines whether this linear relationship holds or not. This would for example suggest that implementing an awareness campaign alone might not be enough to cause the desirable change. Indeed, we show this approach is likely to succeed in only 37% of our participants. Therefore, we have developed a user-friendly framework that comes with our database and executable R-code to guide users in testing the KAP linear relationship as well as integrating quantitative and qualitative data. This should allow for cost-effective planning, designing, implementation, and evaluation of PHI.

## Conclusion

The findings in our study reveal consistencies and inconsistencies with the KAP linear relationship as well as identify associated factors at household level in Uganda. The findings suggest that a pesticide safety awareness campaign would only be effective at causing change in behavior among households with limited knowledge. The opposite would be true for households with high scores on the knowledge metric, which is linked to economic and socio-anthropologic aspects of farming. This highlights why “one-size-fits-all” PHI approaches have not been successful in such a setting. However, by exploiting this framework to establish the nature and distribution of the KAP linear relationship, development and implementation of targeted PHIs can be improved.

## Ethical Approval and Consent to Participate

Ethical approval to conduct the study was obtained from Makerere University School of Public Health. Permission was also sought from the district (district health officer’s office Iganga district) and subcounty officials to carry out the research. Consent was obtained from the respondents before interviewing them. Participation in the study was voluntary and respondents who were unwilling to participate and wanted to quit were told to do so without any restriction. All responses from the farmers interviewed were kept confidential. Rapport was created with the respondents before their actual participation. Research participants were informed of their roles in the study. Risks and benefits involved in the study were told to the participants. A signed copy of the consent form was left to the study participants. There were no human or animal samples involved in our current study.

## Availability of Data and Materials

The datasets generated during and or analyzed during the current study will be publicly available including the R-code used to generate all the results presented in this study manipulating.

## Author Contributions

JM contributed to the design, data collection, and drafting of the manuscript. CK contributed to conception, design, supervision, and drafting of the manuscript. JS contributed to supervision and guidance in design and data collection and manuscript drafting. SM contributed to data analysis and drafting of the manuscript, and AM contributed to conception, design, data analysis, supervision, and drafting of the manuscript. All authors read and approved the final manuscript and have agreed to be accountable for the content of the work.

## Disclaimer

There are no disclaimers for the study and the manuscript in particular.

## Conflict of Interest Statement

The authors declare that the research was conducted in the absence of any commercial or financial relationships that could be construed as a potential conflict of interest.

## Abbreviations

CI, confidence interval; DHO, district health officer; FGD, focus group discussion; IPM, integrated pesticide management; KAP, knowledge, attitude, and practices; OR, odds ratio; PCA, principal component analysis; PPE, personal protective equipment; ROC, receiver operating characteristic; UNHS, Uganda National Household Survey; PHIs, public health interventions.
