# Supplementary material for: A Framework for Integrating Qualitative and Quantitative Data in Knowledge, Attitude, and Practice Studies: A Case Study of Pesticide Usage in Eastern Uganda
Source: Front Public Health. 2017 Dec 8;5:318. doi: 10.3389/fpubh.2017.00318 (PMC5727069; doi:10.3389/fpubh.2017.00318)
Supplement: Supplementary file 1 [file Data_Sheet_1.zip › supplementary Material/KAP supplementary material - R code for quantitative analysis.html]

KAP supplementary material - R code for quantitative analysis
Introduction
The following document provides R code and explanation in order of the
reader to be able to carry out the methodology suggested in our paper
using their own data. Here we provide our own dataset called “DB11.csv”
as an example.
Load libraries
If you do not have these packages please download them using the
function /install.packages(“package name”)/. If any of them is missing
then some of the downstream code will not work!!
|library(knitr)
library(foreign)
library(lme4)
library(boot)
library(parallel)
library(snow)
library(ggplot2)
library(devtools)
library(grid)
library(rms)
library(ResourceSelection)
library(MKmisc)
library(ROCR)
library(pROC)
library(Hmisc)
library(cowplot)|
Read in Data
Here we read in the database, please note that “DB11.csv” contains 167
rows and 157 columns.
\* Columns 1-83 questions from the questionaire
\* 84-91 are the question used for the Attitude metric
\* 92-109 are the question used for the Practice metric
\* 110-120 are the question used for the Knowlege metric
In order to evaluate the contribution (weight) of each question to each
of the metric the response were recorded numerically, columns:
\* 150-157 are the question used for the Attitude metric(GOOD==1,BAD==2)
\* 132-149 are the question used for the Practice
metric(PROPER==1,IMPROPER==2)
\* 121-131 are the question used for the Knowlege metric(YES==1,NO==2)
|PESTICIDE\_DB<-read.csv("DB11.csv",sep = ",",header = T)|
Extract KAP questions and metric
|# The code below subsets questions from which knowledge, attitude and practice matrices will be generated
KNOWLEDGE\_QN<- PESTICIDE\_DB[,c(110:120),]
ATTITUTE\_QN<- PESTICIDE\_DB[,c(84:91),]
PRACTICE\_QN<- PESTICIDE\_DB[,c(92:109),]
## # The code below subsets numerically coded questions for the knowledge, attitude and practice matrices which have been used to evaluated the contribution of each question
KNOWLEDGE\_QN1<- PESTICIDE\_DB[,c(121:131),]
ATTITUTE\_QN1<- PESTICIDE\_DB[,c(150:157),]
PRACTICE\_QN1<- PESTICIDE\_DB[,c(132:149),]
# Here we generate the knowledge matric, which is the proportion of question to which the respondent gave a YES out of the 11 total question used to generate the Knowledge metric
KNOWLEDGE\_QN$Knowledge\_Metric <- apply(KNOWLEDGE\_QN, 1, function(x)
length(which(x == "YES" | x == "YES")) / 11)
# Here we generate the Attitude matric, which is the proportion of question for which the response is indicative of GOOD attitude out of the 8 total question used to generate the Attitude metric
ATTITUTE\_QN$Attitude\_metric <- apply(ATTITUTE\_QN, 1, function(x)
length(which(x == "GOOD" | x == "GOOD")) / 8)
# Here we generate the Pratcice matric, which is the proportion of question for which the response is indicative of PROPER/acceptable practice out of the 18 total question used to generate the Pratcice metric
PRACTICE\_QN$Practice\_metric <- apply(PRACTICE\_QN, 1, function(x)
length(which(x == "PROPER" | x == "PROPER")) / 18)
# When then add each of the columns with the corresponding metric to original database for downstream analysis
PESTICIDE\_DB$Knowledge\_Metric<-KNOWLEDGE\_QN$Knowledge\_Metric
PESTICIDE\_DB$Attitude\_metric<-ATTITUTE\_QN$Attitude\_metric
PESTICIDE\_DB$Practice\_metric<-PRACTICE\_QN$Practice\_metric
# Here we generate the binary variable from the knowledge metric which will be used for logistic regression and univariate regression
PESTICIDE\_DB$knowlege\_binary<-NA
PESTICIDE\_DB$knowlege\_binary[PESTICIDE\_DB$Knowledge\_Metric>=0.50]<-1
PESTICIDE\_DB$knowlege\_binary[PESTICIDE\_DB$Knowledge\_Metric<0.50]<-0 |
Data exploration
Here we use the t.test to explore and associations between the binary
variable of knowledge and our explanatory variables.
|t.test(table(PESTICIDE\_DB$knowlege\_binary,PESTICIDE\_DB$N53PROXI))|
|##
## One Sample t-test
##
## data: table(PESTICIDE\_DB$knowlege\_binary, PESTICIDE\_DB$N53PROXI)
## t = 4.6278, df = 3, p-value = 0.019
## alternative hypothesis: true mean is not equal to 0
## 95 percent confidence interval:
## 12.9613 70.0387
## sample estimates:
## mean of x
## 41.5|
|t.test(table(PESTICIDE\_DB$knowlege\_binary,PESTICIDE\_DB$N47DO))|
|##
## One Sample t-test
##
## data: table(PESTICIDE\_DB$knowlege\_binary, PESTICIDE\_DB$N47DO)
## t = 2.5315, df = 3, p-value = 0.08531
## alternative hypothesis: true mean is not equal to 0
## 95 percent confidence interval:
## -10.67174 93.67174
## sample estimates:
## mean of x
## 41.5|
|t.test(table(PESTICIDE\_DB$knowlege\_binary,PESTICIDE\_DB$N36DO))|
|##
## One Sample t-test
##
## data: table(PESTICIDE\_DB$knowlege\_binary, PESTICIDE\_DB$N36DO)
## t = 2.17, df = 3, p-value = 0.1185
## alternative hypothesis: true mean is not equal to 0
## 95 percent confidence interval:
## -19.36299 102.36299
## sample estimates:
## mean of x
## 41.5|
Visual exploration of the knowledge metric with individual attributes
Here we create a plot which uses the continuous scale of the knowledge
metric against some of the respondents attributes, here we use a
regression line to give us an indication of the direction of the
relationship.
|# Save each plot as an object
PIA1a<-ggplot(PESTICIDE\_DB, aes(x=N5\_AGE, y=Knowledge\_Metric, fill=N4\_SEX, colour=N4\_SEX)) +
labs(title = "Knowledge by sex", x=" ", y="Knowledge (%)") +
scale\_color\_discrete(name="Sex") + theme(legend.title = element\_text(size = 7)) +
geom\_point(aes(size=Attitude\_metric)) + geom\_smooth(method=lm) + theme\_bw() +
theme(legend.position="bottom", plot.title = element\_text(hjust = 0.5)) + guides(fill = "none", color= "none", size = "none") + scale\_size(range = c(1,3))
PIA1b<-ggplot(PESTICIDE\_DB, aes(x=N5\_AGE, y=Knowledge\_Metric, fill=N4\_SEX, colour=N4\_SEX)) +
labs(title = "", x="Age in years", y="Knowledge (%)") +
scale\_fill\_discrete(name="Sex") + theme(legend.title = element\_text(size = 6)) +
geom\_point(aes(size=Practice\_metric)) + geom\_smooth(method=lm) + theme\_bw() +
theme(legend.position="bottom",plot.title = element\_text(hjust = 0.5)) + guides(color = "none",
size = "none",text.font=2) + scale\_size(range = c(1,3)) +
theme(legend.title = element\_text(size = 9)) + theme(legend.text = element\_text(size = 9))+ theme(legend.key.size = unit(0.5, "cm"))
PIA2a<-ggplot(PESTICIDE\_DB, aes(x=N5\_AGE, y=Knowledge\_Metric, fill=N3VILLAG.1,colour=N3VILLAG.1)) +
labs(title = "Knowledge by Residence", x=" ", y=" ") + scale\_color\_discrete(name="Res") +
theme(legend.title = element\_text(size = 5)) + geom\_point(aes(size=Attitude\_metric)) +
geom\_smooth(method=lm) + theme\_bw() + theme(legend.position="bottom") +
guides(fill = "none", color="none",
size = "none",text.font=2)+ scale\_size(range = c(1,3)) +
theme(legend.title = element\_text(size = 9),plot.title = element\_text(hjust = 0.5)) + theme(legend.text = element\_text(size = 9)) + theme(legend.key.size = unit(0.5, "cm"))
PIA2b<-ggplot(PESTICIDE\_DB, aes(x=N5\_AGE, y=Knowledge\_Metric, fill=N3VILLAG.1,colour=N3VILLAG.1)) +
labs(title = "", x="Age in years", y="") +
theme(legend.title = element\_text(size = 5)) +
geom\_point(aes(size=Practice\_metric)) + geom\_smooth(method=lm) +
theme\_bw() +
theme(legend.position="bottom") + scale\_color\_discrete(name="Res") +
guides(fill = "none",size = "none",text.font=2) + scale\_size(range = c(1,3)) +
theme(legend.title = element\_text(size = 9),plot.title = element\_text(hjust = 0.5)) + theme(legend.text = element\_text(size = 9)) + theme(legend.key.size = unit(0.5, "cm"))
PIA3a<-ggplot(PESTICIDE\_DB, aes(x=N5\_AGE, y=Knowledge\_Metric, fill=N6\_MARITAL\_STATUS, colour=N6\_MARITAL\_STATUS)) +
labs(title = "", x="Age in years", y="") + scale\_color\_discrete(name="M Status") +
theme(legend.title = element\_text(size = 5)) + geom\_point(aes(size=Practice\_metric)) +
geom\_smooth(method=lm) + theme\_bw() + theme(legend.position="bottom") +
guides(fill = "none", size = "none",text.font=2) + scale\_size(range = c(1,3)) +
theme(legend.title = element\_text(size = 9),plot.title = element\_text(hjust = 0.5)) + theme(legend.text = element\_text(size = 9))+ theme(legend.key.size = unit(0.5, "cm"))
PIA3b<-ggplot(PESTICIDE\_DB, aes(x=N5\_AGE, y=Knowledge\_Metric, fill=N6\_MARITAL\_STATUS, colour=N6\_MARITAL\_STATUS)) +
labs(title = "Knowledge by Marital status ", x=" ", y="") + scale\_color\_discrete(name="M status") + theme(legend.title = element\_text(size = 5),plot.title = element\_text(hjust = 0.5)) + geom\_point(aes(size=Attitude\_metric)) +
geom\_smooth(method=lm) + theme\_bw() + theme(legend.position="bottom") +
guides(fill = "none", color="none",
size = "none",text.font=2)+ scale\_size(range = c(1,3))
PIA5a<-ggplot(PESTICIDE\_DB, aes(x=N5\_AGE, y=Knowledge\_Metric, fill=PESTICIDE\_DB$N8EDUCAT,colour=N8EDUCAT)) +
labs(title = "Knowledge by Education", x="", y="") + scale\_color\_discrete(name="Educ") +
theme(legend.title = element\_text(size = 5),plot.title = element\_text(hjust = 0.5)) + geom\_point(aes(size=Practice\_metric)) + theme(legend.position="bottom") +
geom\_smooth(method=lm) + theme\_bw() + theme(legend.position="bottom")+ guides(color = "none",fill="none", size = "none",text.font=2)+ scale\_size(range = c(1,3))
PIA5b<-ggplot(PESTICIDE\_DB, aes(x=N5\_AGE, y=Knowledge\_Metric, fill=N8EDUCAT,colour=N8EDUCAT)) +
labs(title = " ", x="Age in years", y=" " ) + scale\_color\_discrete(name="Educ") +
theme(legend.title = element\_text(size = 5)) + geom\_point(aes(size=Attitude\_metric)) + theme(legend.position="bottom") +
geom\_smooth(method=lm) + theme\_bw() + theme(legend.position="bottom")+ guides(fill = "none",
size="none",text.font=2) + scale\_size(range = c(1,3)) +
theme(legend.title = element\_text(size = 9)) + theme(legend.text = element\_text(size = 9)) + theme(legend.key.size = unit(0.5, "cm"))
# use package plot\_grid to show all these figures in one, this comes as part of the cowplot package
plot\_grid(PIA1a, PIA2a,PIA3b, PIA5a,PIA1b, PIA2b,PIA3a,PIA5b,labels=c("A", "B", "C", "D","E","F","G","H"), ncol=4)|
Exploring the relationship between the Practice and Attitude metric
This plot allows us to compare our imperical data with a theorem
proposed by Van Doorn J et al 2017, who stated, that there is a
threshold beyond which practice has linear relationship to
knowlege-attitude. So here we have ploted the practice and attitude
metric to explore this theorem. Note that we have used attitude instead
of knowledge since these two are correlated.
|ggplot(PESTICIDE\_DB, aes(x=Attitude\_metric, y= Practice\_metric)) +
geom\_jitter(width=0.015, height=0.015,aes(colour= as.factor(PESTICIDE\_DB$N8EDUCAT),alpha=0.7,shape= as.factor(PESTICIDE\_DB$N4\_SEX))) +
geom\_vline(xintercept = 0.581, col="grey") + geom\_hline(yintercept = 0.5, col="grey") +
scale\_color\_discrete(name="Education level") + scale\_shape\_discrete(name="Gender") +
theme(legend.title = element\_text(size = 10)) + theme(legend.position="bottom") + theme\_bw() +
theme(plot.title = element\_text(hjust=0.5)) +
labs( x="Percentage score on Attitude metric", y="Percentage score on Pratice metric", title="Relationship between Practice & Attitude") |
NON PARAMETRIC Principal component analysis for KAP linear
relationship
|### The code for ggbiplot was developed by the Vincent Q Vu and can be freely downloaded at https://github.com/vqv/ggbiplot
ggbiplot<-function(pcobj, choices = 1:2, scale = 1, pc.biplot = TRUE,
obs.scale = 1 - scale, var.scale = scale,
groups = NULL, ellipse = FALSE, ellipse.prob = 0.68,
labels = NULL, labels.size = 3, alpha = 1,
var.axes = TRUE,
circle = FALSE, circle.prob = 0.69,
varname.size = 3, varname.adjust = 1.5,
varname.abbrev = FALSE, ...)
{
library(ggplot2)
library(plyr)
library(scales)
library(grid)
stopifnot(length(choices) == 2)
# Recover the SVD
if(inherits(pcobj, 'prcomp')){
nobs.factor <- sqrt(nrow(pcobj$x) - 1)
d <- pcobj$sdev
u <- sweep(pcobj$x, 2, 1 / (d \* nobs.factor), FUN = '\*')
v <- pcobj$rotation
} else if(inherits(pcobj, 'princomp')) {
nobs.factor <- sqrt(pcobj$n.obs)
d <- pcobj$sdev
u <- sweep(pcobj$scores, 2, 1 / (d \* nobs.factor), FUN = '\*')
v <- pcobj$loadings
} else if(inherits(pcobj, 'PCA')) {
nobs.factor <- sqrt(nrow(pcobj$call$X))
d <- unlist(sqrt(pcobj$eig)[1])
u <- sweep(pcobj$ind$coord, 2, 1 / (d \* nobs.factor), FUN = '\*')
v <- sweep(pcobj$var$coord,2,sqrt(pcobj$eig[1:ncol(pcobj$var$coord),1]),FUN="/")
} else if(inherits(pcobj, "lda")) {
nobs.factor <- sqrt(pcobj$N)
d <- pcobj$svd
u <- predict(pcobj)$x/nobs.factor
v <- pcobj$scaling
d.total <- sum(d^2)
} else {
stop('Expected a object of class prcomp, princomp, PCA, or lda')
}
# Scores
choices <- pmin(choices, ncol(u))
df.u <- as.data.frame(sweep(u[,choices], 2, d[choices]^obs.scale, FUN='\*'))
# Directions
v <- sweep(v, 2, d^var.scale, FUN='\*')
df.v <- as.data.frame(v[, choices])
names(df.u) <- c('xvar', 'yvar')
names(df.v) <- names(df.u)
if(pc.biplot) {
df.u <- df.u \* nobs.factor
}
# Scale the radius of the correlation circle so that it corresponds to
# a data ellipse for the standardized PC scores
r <- sqrt(qchisq(circle.prob, df = 2)) \* prod(colMeans(df.u^2))^(1/4)
# Scale directions
v.scale <- rowSums(v^2)
df.v <- r \* df.v / sqrt(max(v.scale))
# Change the labels for the axes
if(obs.scale == 0) {
u.axis.labs <- paste('standardized PC', choices, sep='')
} else {
u.axis.labs <- paste('PC', choices, sep='')
}
# Append the proportion of explained variance to the axis labels
u.axis.labs <- paste(u.axis.labs,
sprintf('(%0.1f%% explained var.)',
100 \* pcobj$sdev[choices]^2/sum(pcobj$sdev^2)))
# Score Labels
if(!is.null(labels)) {
df.u$labels <- labels
}
# Grouping variable
if(!is.null(groups)) {
df.u$groups <- groups
}
# Variable Names
if(varname.abbrev) {
df.v$varname <- abbreviate(rownames(v))
} else {
df.v$varname <- rownames(v)
}
# Variables for text label placement
df.v$angle <- with(df.v, (180/pi) \* atan(yvar / xvar))
df.v$hjust = with(df.v, (1 - varname.adjust \* sign(xvar)) / 2)
# Base plot
g <- ggplot(data = df.u, aes(x = xvar, y = yvar)) +
xlab(u.axis.labs[1]) + ylab(u.axis.labs[2]) + coord\_equal()
if(var.axes) {
# Draw circle
if(circle)
{
theta <- c(seq(-pi, pi, length = 50), seq(pi, -pi, length = 50))
circle <- data.frame(xvar = r \* cos(theta), yvar = r \* sin(theta))
g <- g + geom\_path(data = circle, color = muted('white'),
size = 1/2, alpha = 1/3)
}
# Draw directions
g <- g +
geom\_segment(data = df.v,
aes(x = 0, y = 0, xend = xvar, yend = yvar),
arrow = arrow(length = unit(1/2, 'picas')),
color = muted('red'))
}
# Draw either labels or points
if(!is.null(df.u$labels)) {
if(!is.null(df.u$groups)) {
g <- g + geom\_text(aes(label = labels, color = groups),
size = labels.size)
} else {
g <- g + geom\_text(aes(label = labels), size = labels.size)
}
} else {
if(!is.null(df.u$groups)) {
g <- g + geom\_point(aes(color = groups), alpha = alpha)
} else {
g <- g + geom\_point(alpha = alpha)
}
}
# Overlay a concentration ellipse if there are groups
if(!is.null(df.u$groups) && ellipse) {
theta <- c(seq(-pi, pi, length = 50), seq(pi, -pi, length = 50))
circle <- cbind(cos(theta), sin(theta))
ell <- ddply(df.u, 'groups', function(x) {
if(nrow(x) <= 2) {
return(NULL)
}
sigma <- var(cbind(x$xvar, x$yvar))
mu <- c(mean(x$xvar), mean(x$yvar))
ed <- sqrt(qchisq(ellipse.prob, df = 2))
data.frame(sweep(circle %\*% chol(sigma) \* ed, 2, mu, FUN = '+'),
groups = x$groups[1])
})
names(ell)[1:2] <- c('xvar', 'yvar')
g <- g + geom\_path(data = ell, aes(color = groups, group = groups))
}
# Label the variable axes
if(var.axes) {
g <- g +
geom\_text(data = df.v,
aes(label = varname, x = xvar, y = yvar,
angle = angle, hjust = hjust),
color = 'darkred', size = varname.size)
}
# Change the name of the legend for groups
# if(!is.null(groups)) {
# g <- g + scale\_color\_brewer(name = deparse(substitute(groups)),
# palette = 'Dark2')
# }
# TODO: Add a second set of axes
return(g)
}
## Here we convert all the variables to be used in this principal component analysis to log for scaling purposes
PESTICIDE\_DB$Knowledge<-log(PESTICIDE\_DB$Knowledge\_Metric)
PESTICIDE\_DB$Attitude<-log(PESTICIDE\_DB$Attitude\_metric)
PESTICIDE\_DB$Practice<-log(PESTICIDE\_DB$Practice\_metric)
PESTICIDE\_DB$Experience<-log(PESTICIDE\_DB$N11HOW)
PESTICIDE\_DB$AGE<-log(PESTICIDE\_DB$N5\_AGE)
PESTICIDE\_DB$SEX<-NA
PESTICIDE\_DB$SEX[PESTICIDE\_DB$N4\_SEX=="MALE"]<-1
PESTICIDE\_DB$SEX[PESTICIDE\_DB$N4\_SEX=="FEMALE"]<-2
PESTICIDE\_DB$Gender<-log(PESTICIDE\_DB$SEX)
PESTICIDE\_DBPC<- PESTICIDE\_DB[-155,] # removes a row with an infinite outcome for the knowldge metric
KAP.pca\_2 <- prcomp(PESTICIDE\_DBPC[,c(162:164,166,168),],
center = TRUE,
scale. = TRUE)
print(KAP.pca\_2)|
|## Standard deviations:
## [1] 1.4943215 1.0502814 0.9627093 0.7170930 0.4721024
##
## Rotation:
## PC1 PC2 PC3 PC4 PC5
## Knowledge -0.58967221 -0.03715822 -0.1581479 -0.4513610 0.64974493
## Attitude -0.60861516 -0.04595541 -0.1533739 -0.2202232 -0.74528779
## Practice -0.52338316 0.09490830 0.1938289 0.8120403 0.14171572
## AGE -0.05890921 0.74727668 0.6061113 -0.2620835 -0.04526196
## Gender -0.06688837 -0.65503995 0.7392734 -0.1402774 -0.01567319|
|summary(KAP.pca\_2)|
|## Importance of components:
## PC1 PC2 PC3 PC4 PC5
## Standard deviation 1.4943 1.0503 0.9627 0.7171 0.47210
## Proportion of Variance 0.4466 0.2206 0.1854 0.1028 0.04458
## Cumulative Proportion 0.4466 0.6672 0.8526 0.9554 1.00000|
|screeplot(KAP.pca\_2,type="lines",col=3)|
|g\_1 <- ggbiplot(KAP.pca\_2, obs.scale = 1, var.scale = 1,
groups = PESTICIDE\_DBPC$N3VILLAG, ellipse = TRUE,
circle = TRUE)
g\_1 <- g\_1 + scale\_color\_discrete(name = '')
g\_1 <- g\_1 + theme(legend.direction = 'horizontal',
legend.position = 'top') + theme\_bw()
print(g\_1)|
Principal component analysis: When data is split into low and high
knowledge score
In the biplot above you can see that there are two populations spread
along PC1, we though that this could have an effect on the linearity of
the KAP metrics, so we divided the sample into those who scored high and
low on knowldge metric to see if the KAP axiom holds. Below is the
analysis with the the group that score low on the knowledge metric.
|PESTICIDE\_DBLOW<-PESTICIDE\_DBPC[PESTICIDE\_DBPC$Knowledge\_Metric<=0.5,]
PESTICIDE\_DBHIGH<-PESTICIDE\_DBPC[PESTICIDE\_DBPC$Knowledge\_Metric>=0.5,]
KAP.pca\_lowscore <- prcomp(PESTICIDE\_DBLOW[,c(162:164,166,168),],
center = TRUE,
scale. = TRUE)
print(KAP.pca\_lowscore)|
|## Standard deviations:
## [1] 1.4862985 1.0096148 0.9980412 0.6697181 0.5718270
##
## Rotation:
## PC1 PC2 PC3 PC4 PC5
## Knowledge 0.5598110 -0.08380318 -0.06835079 0.78659497 -0.2370341
## Attitude 0.5590189 0.08092486 -0.24561717 -0.57336863 -0.5402496
## Practice 0.5933834 0.07386176 0.06552265 -0.17378257 0.7797095
## AGE 0.1441984 -0.49892670 0.82861326 -0.13248577 -0.1616371
## Gender 0.0348196 0.85559616 0.49407209 0.06902103 -0.1336850|
|summary(KAP.pca\_lowscore)|
|## Importance of components:
## PC1 PC2 PC3 PC4 PC5
## Standard deviation 1.4863 1.0096 0.9980 0.6697 0.5718
## Proportion of Variance 0.4418 0.2039 0.1992 0.0897 0.0654
## Cumulative Proportion 0.4418 0.6457 0.8449 0.9346 1.0000|
|screeplot(KAP.pca\_lowscore,type="lines",col=3)|
|g\_lowscore <- ggbiplot(KAP.pca\_lowscore, obs.scale = 1, var.scale = 1,
groups = PESTICIDE\_DBLOW$N3VILLAG, ellipse = TRUE,
circle = TRUE)
g\_lowscore <- g\_lowscore + scale\_color\_discrete(name = '')
g\_lowscore <- g\_lowscore + theme(legend.direction = 'horizontal',
legend.position = 'top') + theme\_bw()
print(g\_lowscore)|
|## Here is the analysis for the group that score high on the knowledge metric
KAP.pca\_highscore <- prcomp(PESTICIDE\_DBHIGH[,c(162:164,166,168),],
center = TRUE,
scale. = TRUE)
print(KAP.pca\_highscore)|
|## Standard deviations:
## [1] 1.4272010 1.0693251 0.9260639 0.7930930 0.5771052
##
## Rotation:
## PC1 PC2 PC3 PC4 PC5
## Knowledge -0.50004617 0.0002919966 -0.3134344 0.7999378 0.108683775
## Attitude -0.59425108 -0.1271512873 0.2918169 -0.1590872 -0.721271329
## Practice 0.58980495 0.0216239897 -0.2619102 0.3577170 -0.674614063
## AGE 0.20998373 -0.6642403404 0.6124233 0.3560681 0.113335368
## Gender 0.06968815 0.7363081136 0.6106903 0.2829220 -0.002543151|
|summary(KAP.pca\_highscore)|
|## Importance of components:
## PC1 PC2 PC3 PC4 PC5
## Standard deviation 1.4272 1.0693 0.9261 0.7931 0.57711
## Proportion of Variance 0.4074 0.2287 0.1715 0.1258 0.06661
## Cumulative Proportion 0.4074 0.6361 0.8076 0.9334 1.00000|
|screeplot(KAP.pca\_highscore,type="lines",col=3)|
|g\_highscore <- ggbiplot(KAP.pca\_highscore, obs.scale = 1, var.scale = 1,
groups = PESTICIDE\_DBHIGH$N3VILLAG, ellipse = TRUE,
circle = TRUE)
g\_highscore <- g\_highscore + scale\_color\_discrete(name = '')
g\_highscore <- g\_highscore + theme(legend.direction = 'horizontal',
legend.position = 'top') + theme\_bw()
print(g\_highscore)|
PARAMETRIC ANALYSIS Evaluating the linear relationship using
Pearson’s correlation coefficient
Low score on knowledge metric
Here would like to see if the same is true if we used a parametric
analysis, so we run the knowledge metrics for the group that had a low
score and the attitude and practices using the pearson correlation
coefficient. A positive correlation suggests a linear relation ship and
negative suggests the opposite.
|cor.test(PESTICIDE\_DBLOW$Knowledge\_Metric,PESTICIDE\_DBLOW$Attitude\_metric)|
|##
## Pearson's product-moment correlation
##
## data: PESTICIDE\_DBLOW$Knowledge\_Metric and PESTICIDE\_DBLOW$Attitude\_metric
## t = 5.9772, df = 60, p-value = 1.341e-07
## alternative hypothesis: true correlation is not equal to 0
## 95 percent confidence interval:
## 0.4261791 0.7467421
## sample estimates:
## cor
## 0.6109176|
|cor.test(PESTICIDE\_DBLOW$Knowledge\_Metric,PESTICIDE\_DBLOW$Practice\_metric)|
|##
## Pearson's product-moment correlation
##
## data: PESTICIDE\_DBLOW$Knowledge\_Metric and PESTICIDE\_DBLOW$Practice\_metric
## t = 6.8547, df = 60, p-value = 4.425e-09
## alternative hypothesis: true correlation is not equal to 0
## 95 percent confidence interval:
## 0.4948473 0.7828889
## sample estimates:
## cor
## 0.6627069|
|cor.test(PESTICIDE\_DBLOW$Attitude\_metric,PESTICIDE\_DBLOW$Practice\_metric)|
|##
## Pearson's product-moment correlation
##
## data: PESTICIDE\_DBLOW$Attitude\_metric and PESTICIDE\_DBLOW$Practice\_metric
## t = 7.4575, df = 60, p-value = 4.14e-10
## alternative hypothesis: true correlation is not equal to 0
## 95 percent confidence interval:
## 0.5367820 0.8040466
## sample estimates:
## cor
## 0.6935634|
High score on knowledge metric
This is the same analysis as above on the group that had a high score on
the knowledge metric, please read the article for to get the interpretation.
|cor.test(PESTICIDE\_DBHIGH$Knowledge\_Metric,PESTICIDE\_DBHIGH$Attitude\_metric)|
|##
## Pearson's product-moment correlation
##
## data: PESTICIDE\_DBHIGH$Knowledge\_Metric and PESTICIDE\_DBHIGH$Attitude\_metric
## t = 4.4724, df = 102, p-value = 2.013e-05
## alternative hypothesis: true correlation is not equal to 0
## 95 percent confidence interval:
## 0.2302799 0.5542759
## sample estimates:
## cor
## 0.4049107|
|cor.test(PESTICIDE\_DBHIGH$Knowledge\_Metric,PESTICIDE\_DBHIGH$Practice\_metric)|
|##
## Pearson's product-moment correlation
##
## data: PESTICIDE\_DBHIGH$Knowledge\_Metric and PESTICIDE\_DBHIGH$Practice\_metric
## t = -4.1324, df = 102, p-value = 7.372e-05
## alternative hypothesis: true correlation is not equal to 0
## 95 percent confidence interval:
## -0.5324493 -0.2007463
## sample estimates:
## cor
## -0.3786937|
|cor.test(PESTICIDE\_DBHIGH$Attitude\_metric,PESTICIDE\_DBHIGH$Practice\_metric)|
|##
## Pearson's product-moment correlation
##
## data: PESTICIDE\_DBHIGH$Attitude\_metric and PESTICIDE\_DBHIGH$Practice\_metric
## t = -8.5994, df = 102, p-value = 1.007e-13
## alternative hypothesis: true correlation is not equal to 0
## 95 percent confidence interval:
## -0.7475488 -0.5207208
## sample estimates:
## cor
## -0.648295|
Evaluating the weight of questions included in a questionnaire
Attitude questions
This analysis aims at evaluating the weight each question brings to the
corresponding metric. We have used a PCA and we taking the correlation
coefficient for each question in the PC1 (which explains the largest
variation) as the weight.
|ATTITUTE\_QN1<-ATTITUTE\_QN1[,-2]
KAP.pca\_Att <- prcomp(ATTITUTE\_QN1,
center = TRUE,
scale. = TRUE)
screeplot(KAP.pca\_Att,type="lines",col=3)|
|summary(KAP.pca\_Att)|
|## Importance of components:
## PC1 PC2 PC3 PC4 PC5 PC6
## Standard deviation 2.2587 1.0632 0.63814 0.45354 0.28095 0.21209
## Proportion of Variance 0.7288 0.1615 0.05817 0.02939 0.01128 0.00643
## Cumulative Proportion 0.7288 0.8903 0.94850 0.97789 0.98917 0.99559
## PC7
## Standard deviation 0.17566
## Proportion of Variance 0.00441
## Cumulative Proportion 1.00000|
|print(KAP.pca\_Att)|
|## Standard deviations:
## [1] 2.2587211 1.0632461 0.6381355 0.4535430 0.2809490 0.2120871 0.1756564
##
## Rotation:
## PC1 PC2 PC3 PC4
## ReCoded\_N30DO 0.4126221 0.076337501 -0.35266369 0.5328275
## ReCoded\_N32MIXIN -0.3403354 0.323779426 -0.80813767 -0.3400460
## ReCoded\_N33DO 0.4228886 0.074070425 -0.29827104 0.3490422
## ReCoded\_N35DO 0.4224837 0.003741583 0.10416918 -0.4279425
## ReCoded\_N36DO 0.4315658 0.060451966 -0.08956715 -0.2437587
## ReCoded\_N37AFTER 0.1611698 -0.843930484 -0.31944755 -0.2678525
## ReCoded\_N38DO 0.3799513 0.409836480 0.11249878 -0.4053826
## PC5 PC6 PC7
## ReCoded\_N30DO -0.048880716 0.4457038 0.463246758
## ReCoded\_N32MIXIN -0.100351011 0.0225254 -0.006576403
## ReCoded\_N33DO -0.001769396 -0.3217010 -0.708087644
## ReCoded\_N35DO -0.751683761 0.2274821 -0.103590527
## ReCoded\_N36DO 0.089501546 -0.6974090 0.498263057
## ReCoded\_N37AFTER 0.248439978 0.1511190 -0.058775236
## ReCoded\_N38DO 0.593949319 0.3693031 -0.146695202|
Practice questions
|PRACTICE\_QN1<-PRACTICE\_QN1[,-c(2,6),]# removed columes with NA
KAP.pca\_prac <- prcomp(PRACTICE\_QN1,
center = TRUE,
scale. = TRUE)
screeplot(KAP.pca\_prac,type="lines",col=3)|
| print(KAP.pca\_prac)|
|## Standard deviations:
## [1] 3.295592e+00 1.727973e+00 8.690084e-01 7.257374e-01 5.432658e-01
## [6] 3.902441e-01 3.702052e-01 3.391939e-01 2.706090e-01 1.802173e-01
## [11] 1.368119e-01 1.150142e-01 1.094476e-01 1.078571e-01 1.025475e-01
## [16] 1.737463e-16
##
## Rotation:
## PC1 PC2 PC3 PC4
## ReCoded\_N39DO -0.2640956 -0.190254066 0.103409098 -0.09378346
## ReCoded\_N42ON 0.2566397 0.280523682 0.133235281 -0.05418094
## ReCoded\_N43HOW -0.1621634 0.243880340 -0.503263939 -0.80833407
## ReCoded\_N44DO 0.2526442 -0.282943672 0.021982087 -0.19798866
## ReCoded\_N46IN -0.2649780 -0.005875491 0.403935178 -0.21759403
## ReCoded\_N47DO 0.2465466 -0.306926016 0.133509490 -0.20794756
## ReCoded\_N48IF -0.2649790 0.097982345 0.449898987 -0.19034558
## ReCoded\_N49LOCAT -0.2645922 0.107834157 0.440076533 -0.17869645
## ReCoded\_N50WHERE 0.2623812 0.252931561 0.037893875 0.01362465
## ReCoded\_N51HOW 0.2535798 -0.274542670 -0.005826879 -0.18476357
## ReCoded\_N52DOES -0.2505147 -0.285678270 -0.168033445 0.08144654
## ReCoded\_N53PROXI -0.2549804 -0.283840630 -0.148146097 0.06535515
## ReCoded\_N54PROXI -0.2312334 -0.275614929 -0.198724467 0.11085960
## ReCoded\_N55REACH 0.2444080 -0.310116219 0.156950061 -0.19837257
## ReCoded\_N56STORA 0.2444080 -0.310116219 0.156950061 -0.19837257
## ReCoded\_N58WHAT 0.2630273 0.245509490 0.017938433 0.02628992
## PC5 PC6 PC7 PC8
## ReCoded\_N39DO -0.467903753 0.16548731 -0.2972482157 -0.193547436
## ReCoded\_N42ON -0.046436521 0.16445793 -0.2598258180 -0.242619076
## ReCoded\_N43HOW 0.043678872 -0.06156072 -0.0414404432 0.008946593
## ReCoded\_N44DO -0.007415807 0.37570807 0.3450130465 -0.119451301
## ReCoded\_N46IN -0.038739713 0.45262086 -0.2351401104 0.628520236
## ReCoded\_N47DO 0.062322675 -0.18636755 -0.1335604741 0.030577856
## ReCoded\_N48IF 0.233530436 -0.10524043 0.2153440157 -0.235879291
## ReCoded\_N49LOCAT 0.241715044 -0.14494042 0.2221929011 -0.272980767
## ReCoded\_N50WHERE 0.336822609 0.12003237 -0.0506036044 0.181695445
## ReCoded\_N51HOW -0.023557228 0.44016968 0.3877986298 -0.125633175
## ReCoded\_N52DOES 0.248990493 -0.05647594 0.1421390965 0.212434929
## ReCoded\_N53PROXI 0.120539940 -0.14540741 0.2531392014 0.274244423
## ReCoded\_N54PROXI 0.556831340 0.33962042 -0.4626673134 -0.368481841
## ReCoded\_N55REACH 0.073839784 -0.29387263 -0.2271762775 0.061154288
## ReCoded\_N56STORA 0.073839784 -0.29387263 -0.2271762775 0.061154288
## ReCoded\_N58WHAT 0.383540162 0.09157097 -0.0002876316 0.225393867
## PC9 PC10 PC11 PC12
## ReCoded\_N39DO 0.7050125967 -4.128228e-02 6.114795e-05 -1.963471e-04
## ReCoded\_N42ON -0.0690047758 5.426250e-01 -8.491259e-07 -5.460106e-06
## ReCoded\_N43HOW -0.0002205906 2.136345e-07 7.150670e-03 8.695835e-04
## ReCoded\_N44DO 0.0083620174 -2.953236e-05 -1.141874e-01 7.224731e-01
## ReCoded\_N46IN -0.2471446374 3.906730e-03 -1.187396e-03 5.847543e-03
## ReCoded\_N47DO -0.0004868588 -5.300335e-06 -8.209284e-01 -2.285789e-01
## ReCoded\_N48IF 0.0404056085 -1.268303e-04 1.363467e-02 -9.897727e-02
## ReCoded\_N49LOCAT 0.0559704044 -4.406191e-04 -3.045603e-03 7.722440e-02
## ReCoded\_N50WHERE 0.4438029279 -5.912433e-02 1.058780e-05 -6.972048e-05
## ReCoded\_N51HOW 0.0071885560 -8.985929e-06 2.339669e-01 -6.341968e-01
## ReCoded\_N52DOES 0.2035750976 7.775703e-01 -1.316393e-06 6.826500e-06
## ReCoded\_N53PROXI 0.1490527215 -2.294273e-01 1.998286e-06 -1.842411e-05
## ReCoded\_N54PROXI -0.1326498730 -1.605919e-01 1.429133e-07 -5.060290e-07
## ReCoded\_N55REACH -0.0025701512 6.040158e-06 3.592002e-01 6.238425e-02
## ReCoded\_N56STORA -0.0025701512 6.040158e-06 3.592002e-01 6.238425e-02
## ReCoded\_N58WHAT 0.3924259813 -1.315311e-01 -1.372033e-06 5.003219e-05
## PC13 PC14 PC15 PC16
## ReCoded\_N39DO -1.036916e-03 -5.713060e-02 2.046981e-02 -2.005259e-17
## ReCoded\_N42ON -3.105274e-04 2.047470e-01 5.814241e-01 3.769177e-17
## ReCoded\_N43HOW -5.924892e-05 -7.073999e-08 1.326567e-09 4.035117e-17
## ReCoded\_N44DO -8.270957e-02 -1.137801e-04 3.381114e-06 -2.049116e-16
## ReCoded\_N46IN 3.757430e-02 1.717935e-03 -5.338047e-04 9.653998e-17
## ReCoded\_N47DO 1.954120e-02 2.493065e-05 -5.864411e-07 7.035709e-16
## ReCoded\_N48IF -7.087340e-01 -1.405818e-03 1.058855e-04 -1.000650e-16
## ReCoded\_N49LOCAT 6.930609e-01 1.294298e-03 -8.041482e-05 3.013350e-17
## ReCoded\_N50WHERE -1.404830e-03 6.345313e-01 -3.147067e-01 -6.194482e-17
## ReCoded\_N51HOW 9.315845e-02 1.368269e-04 -5.000569e-06 1.054817e-16
## ReCoded\_N52DOES 1.890609e-04 -1.053711e-01 -1.818848e-01 -4.256448e-17
## ReCoded\_N53PROXI -5.894229e-04 3.423782e-01 6.790887e-01 -2.113945e-17
## ReCoded\_N54PROXI -1.259369e-05 6.802042e-03 1.054175e-02 8.952886e-17
## ReCoded\_N55REACH -4.703774e-03 -5.786264e-06 1.202605e-07 7.071068e-01
## ReCoded\_N56STORA -4.703774e-03 -5.786264e-06 1.202605e-07 -7.071068e-01
## ReCoded\_N58WHAT 1.325747e-03 -6.510038e-01 2.610204e-01 2.565328e-17|
| summary(KAP.pca\_prac)|
|## Importance of components:
## PC1 PC2 PC3 PC4 PC5 PC6
## Standard deviation 3.2956 1.7280 0.8690 0.72574 0.54327 0.39024
## Proportion of Variance 0.6788 0.1866 0.0472 0.03292 0.01845 0.00952
## Cumulative Proportion 0.6788 0.8654 0.9126 0.94554 0.96399 0.97351
## PC7 PC8 PC9 PC10 PC11 PC12
## Standard deviation 0.37021 0.33919 0.27061 0.18022 0.13681 0.11501
## Proportion of Variance 0.00857 0.00719 0.00458 0.00203 0.00117 0.00083
## Cumulative Proportion 0.98207 0.98926 0.99384 0.99587 0.99704 0.99787
## PC13 PC14 PC15 PC16
## Standard deviation 0.10945 0.10786 0.10255 1.737e-16
## Proportion of Variance 0.00075 0.00073 0.00066 0.000e+00
## Cumulative Proportion 0.99862 0.99934 1.00000 1.000e+00|
Knowledge questions
|KAP.pca\_know <- prcomp(KNOWLEDGE\_QN1,
center = TRUE,
scale. = TRUE)
print(KAP.pca\_know)|
|## Standard deviations:
## [1] 2.1919002 1.2310601 1.0817902 0.9162944 0.8231440 0.7590523 0.6299403
## [8] 0.6078018 0.5891423 0.4427993 0.3272073
##
## Rotation:
## PC1 PC2 PC3
## ReCoded\_Mixingpesticides -0.2772244 0.07471687 0.38159525
## ReCoded\_Alternativetopesticides -0.3874795 -0.17278065 -0.01201387
## ReCoded\_Trainingonpesticdeuse -0.3575161 -0.29646031 -0.10283461
## ReCoded\_KnowledgeonPPE -0.2452812 0.31787483 -0.47417258
## ReCoded\_FarmerswearPPE -0.2949953 0.46010033 -0.03586822
## ReCoded\_SpiltPesticides -0.4260238 -0.03347768 0.13220746
## ReCoded\_Knowledgeonmarks -0.3721320 -0.18338343 0.11794089
## ReCoded\_trainingonsafePecdehandlg -0.2529991 -0.46196200 -0.14250318
## ReCoded\_Pesticideexposuresymptoms -0.2017756 0.22050221 -0.59819159
## ReCoded\_COntainers -0.2484850 0.16385466 0.35555998
## ReCoded\_Effectonhealth -0.1075092 0.49021433 0.28563530
## PC4 PC5 PC6
## ReCoded\_Mixingpesticides -0.27325231 0.49624869 -0.527797492
## ReCoded\_Alternativetopesticides 0.01877308 0.15433617 0.308811404
## ReCoded\_Trainingonpesticdeuse 0.19542188 -0.03608142 0.036193320
## ReCoded\_KnowledgeonPPE -0.19838994 0.26837624 0.416771602
## ReCoded\_FarmerswearPPE 0.10263355 0.13048241 0.146684907
## ReCoded\_SpiltPesticides -0.05717022 0.04353041 -0.006212966
## ReCoded\_Knowledgeonmarks -0.10606622 -0.07217167 -0.093284930
## ReCoded\_trainingonsafePecdehandlg 0.42766646 -0.11105732 -0.049415844
## ReCoded\_Pesticideexposuresymptoms -0.06857584 -0.33751185 -0.612059744
## ReCoded\_COntainers -0.36164295 -0.70696098 0.197331996
## ReCoded\_Effectonhealth 0.70992498 -0.08011740 -0.069286975
## PC7 PC8 PC9
## ReCoded\_Mixingpesticides 0.36163415 -0.135973544 0.05078222
## ReCoded\_Alternativetopesticides -0.06827782 0.130556940 0.37856456
## ReCoded\_Trainingonpesticdeuse 0.20410527 0.378731865 0.45107125
## ReCoded\_KnowledgeonPPE 0.12963440 -0.507627765 0.05232511
## ReCoded\_FarmerswearPPE 0.05554218 0.583445029 -0.52794814
## ReCoded\_SpiltPesticides -0.21672269 -0.001951938 -0.10248642
## ReCoded\_Knowledgeonmarks -0.70314683 -0.201315405 -0.18533800
## ReCoded\_trainingonsafePecdehandlg 0.39037715 -0.304809874 -0.49124879
## ReCoded\_Pesticideexposuresymptoms -0.04306544 0.054882877 0.12829870
## ReCoded\_COntainers 0.31563756 -0.097749440 0.01705138
## ReCoded\_Effectonhealth -0.08803238 -0.277356018 0.25738548
## PC10 PC11
## ReCoded\_Mixingpesticides 0.013606448 0.14288901
## ReCoded\_Alternativetopesticides 0.724104422 0.10478685
## ReCoded\_Trainingonpesticdeuse -0.576943807 0.10719508
## ReCoded\_KnowledgeonPPE -0.223003919 0.04370898
## ReCoded\_FarmerswearPPE 0.009885230 0.16912479
## ReCoded\_SpiltPesticides -0.081727442 -0.85472034
## ReCoded\_Knowledgeonmarks -0.178798593 0.43307610
## ReCoded\_trainingonsafePecdehandlg 0.128986503 0.03653583
## ReCoded\_Pesticideexposuresymptoms 0.193028819 -0.03179217
## ReCoded\_COntainers 0.015425240 0.07585450
## ReCoded\_Effectonhealth -0.003337038 0.01991757|
|summary(KAP.pca\_know)|
|## Importance of components:
## PC1 PC2 PC3 PC4 PC5 PC6 PC7
## Standard deviation 2.1919 1.2311 1.0818 0.91629 0.8231 0.75905 0.62994
## Proportion of Variance 0.4368 0.1378 0.1064 0.07633 0.0616 0.05238 0.03607
## Cumulative Proportion 0.4368 0.5745 0.6809 0.75725 0.8188 0.87123 0.90730
## PC8 PC9 PC10 PC11
## Standard deviation 0.60780 0.58914 0.44280 0.32721
## Proportion of Variance 0.03358 0.03155 0.01782 0.00973
## Cumulative Proportion 0.94089 0.97244 0.99027 1.00000|
|screeplot(KAP.pca\_know,type="lines",col=3)|
Logistic regression model
The code below assumes that you have run the t.test and univariable
regression on all the variables to identify which one can be included in
the model. Below we run a logistic regression and extract the odds
ratios. We also evaluate the model fit using the HL test as well as the AUC.
|PESTICIDE\_DB$N31IF\_REF = relevel(PESTICIDE\_DB$N31IF, ref= "windy")
mylogit<-glm(knowlege\_binary ~ N53PROXI+N31IF\_REF+N36DO +N47DO , data = PESTICIDE\_DB, family = "binomial")
summary((mylogit))|
|##
## Call:
## glm(formula = knowlege\_binary ~ N53PROXI + N31IF\_REF + N36DO +
## N47DO, family = "binomial", data = PESTICIDE\_DB)
##
## Deviance Residuals:
## Min 1Q Median 3Q Max
## -2.3343 -1.0744 0.5719 0.8987 1.6408
##
## Coefficients:
## Estimate Std. Error z value Pr(>|z|)
## (Intercept) 0.63108 0.85198 0.741 0.4589
## N53PROXI>10metres(far) -0.81406 0.38827 -2.097 0.0360 \*
## N31IF\_REFNOt-sure 0.22873 0.87726 0.261 0.7943
## N31IF\_REFrainy 0.06698 0.59828 0.112 0.9109
## N31IF\_REFvery-sunny&dry 1.22817 0.62434 1.967 0.0492 \*
## N36DOYES 0.79747 0.50304 1.585 0.1129
## N47DOYES -0.92875 0.50322 -1.846 0.0649 .
## ---
## Signif. codes: 0 '\*\*\*' 0.001 '\*\*' 0.01 '\*' 0.05 '.' 0.1 ' ' 1
##
## (Dispersion parameter for binomial family taken to be 1)
##
## Null deviance: 215.58 on 161 degrees of freedom
## Residual deviance: 193.40 on 155 degrees of freedom
## (5 observations deleted due to missingness)
## AIC: 207.4
##
## Number of Fisher Scoring iterations: 4|
|exp(cbind(Odds\_and\_OR=coef(mylogit), confint(mylogit)))|
|## Odds\_and\_OR 2.5 % 97.5 %
## (Intercept) 1.8796467 0.3543791 10.3622633
## N53PROXI>10metres(far) 0.4430542 0.2021419 0.9327032
## N31IF\_REFNOt-sure 1.2570000 0.2263677 7.4151561
## N31IF\_REFrainy 1.0692755 0.3283295 3.5380834
## N31IF\_REFvery-sunny&dry 3.4149579 1.0049232 11.9752099
## N36DOYES 2.2199212 0.8422995 6.1597782
## N47DOYES 0.3950468 0.1380482 1.0152292|
|PESTICIDE\_DB$knowlege\_binary<- as.numeric(PESTICIDE\_DB$knowlege\_binary)
mylogit\_l<- lrm(knowlege\_binary ~ N53PROXI+N31IF+N36DO +N47DO , data = PESTICIDE\_DB, method = "lrm.fit" ,
model = T, x = T, y = T,
linear.predictors = T, se.fit = F)
mylogit\_l|
|##
## Logistic Regression Model
##
## lrm(formula = knowlege\_binary ~ N53PROXI + N31IF + N36DO + N47DO,
## data = PESTICIDE\_DB, method = "lrm.fit", model = T, x = T,
## y = T, linear.predictors = T, se.fit = F)
## Frequencies of Missing Values Due to Each Variable
## knowlege\_binary N53PROXI N31IF N36DO
## 0 1 5 1
## N47DO
## 1
##
##
## Model Likelihood Discrimination Rank Discrim.
## Ratio Test Indexes Indexes
## Obs 162 LR chi2 22.18 R2 0.174 C 0.708
## 0 62 d.f. 6 g 0.930 Dxy 0.415
## 1 100 Pr(> chi2) 0.0011 gr 2.534 gamma 0.464
## max |deriv| 2e-09 gp 0.197 tau-a 0.197
## Brier 0.204
##
## Coef S.E. Wald Z Pr(>|Z|)
## Intercept 0.8598 0.8980 0.96 0.3383
## N53PROXI=>10metres(far) -0.8141 0.3883 -2.10 0.0360
## N31IF=rainy -0.1617 0.7247 -0.22 0.8234
## N31IF=very-sunny&dry 0.9994 0.7671 1.30 0.1926
## N31IF=windy -0.2287 0.8773 -0.26 0.7943
## N36DO=YES 0.7975 0.5030 1.59 0.1129
## N47DO=YES -0.9288 0.5032 -1.85 0.0649|
|# goodness of fit evaluation
residuals(mylogit\_l,type = "gof")|
|## Sum of squared errors Expected value|H0 SD
## 33.1023999 33.2423542 0.2012340
## Z P
## -0.6954802 0.4867544|
|prob <- predict(mylogit, newdata=PESTICIDE\_DB, type="response")
pred <- prediction(prob, PESTICIDE\_DB$knowlege\_binary)
perf <- performance(pred, measure = "tpr", x.measure = "fpr")
plot(perf)|
|auc <- performance(pred, measure = "auc")
auc <- auc@y.values[[1]]
auc|
|## [1] 0.7075|
