# Supplementary material for: A Framework for Integrating Qualitative and Quantitative Data in Knowledge, Attitude, and Practice Studies: A Case Study of Pesticide Usage in Eastern Uganda
Source: Front Public Health. 2017 Dec 8;5:318. doi: 10.3389/fpubh.2017.00318 (PMC5727069; doi:10.3389/fpubh.2017.00318)
Supplement: Supplementary file 1 [file Data_Sheet_1.zip › supplementary Material/Supplementary S1.pdf]

ASSESSMENT OF KNOWLEDGE, ATTITUDES AND PRACTICES ON PESTICIDE USAGE AMONG FARMERS IN NABITENDE SUB COUNTY FARMERS' SEMI STRUCTURED QUESTIONNAIRE

Date	…………/……………../…………
Parish	……………………..
Village	……………………..

- **CIRCLE THE MOST APPROPRIATE RESPONSE (S)**

**A. SOCIO-DEMOGRAPHICS**		
**S/N**	**VARIABLE**	**RESPONSE**
A1.	Sex	1. Male<br>2. Female
A2.	Age (In full years)	…………………………………….
A3.	Marital status	1. Married<br>2. Single<br>3. Widowed<br>4. Others (specify):……………………….
A4.	Number of dependents	1. None<br>2. One<br>3. Two<br>4. Above two
A5.	Education Level	1. Never<br>2. Primary<br>3. Secondary<br>4. Tertiary and above
A6.	Do you carry out farming?	1. Yes<br>2. No
A7.	What type of farming do you practice?	1. Subsistence farming<br>2. Commercial farming
A8.	Who owns the land where you do farming?	1. Family<br>2. Relative

		3. Friend<br>4. Other, (Specify): ……………………
A9.	How long have you spent in crop growing? (in years)	………………………………………………

B. KNOWLEDGE ASSESSMENT

B1.	Do you mix different types of pesticides for effectiveness	1. Yes<br>2. No
B2.	Have you ever had any training on how to use and handle pesticides?	1. Yes<br>2. No
B3.	Should farmers wear PPE?	1. Yes<br>2. NO
B4.	Can you understand the information on pesticide packages?	1. Yes<br>2. No
B5.	Do you know the pesticide exposure symptoms?	1. Yes<br>2. No
B6.	Do you think pesticides can have a negative effect on your health?	1. Yes<br>2. No
B7.	Do you know of any alternative to pesticide?	1. Yes<br>2. NO
B8.	Do you know about PPE?	1. Yes<br>2. No
B9.	Have you ever spilt pesticides on your body?	1. Yes<br>2. No
B10.	Have you been trained about safe pesticide handling methods?	1. Yes<br>2. NO
B11.	Do the pesticide containers have any symptoms marking their toxicity?	1. Yes<br>2. No

C. ATTITUDE ASSESSMENT

C1	Do you think it is important to observe weather conditions when going to spray?	1. Yes<br>2. NO
C2.	Mixing of different types of chemicals is very important?	1. Yes<br>2. No
C3.	If yes, in which ways can they enter?	…………………………………………
C4.	Do you care about the harmful effects of pesticide exposure?	1. Yes<br>2. No
C5.	After spraying, we can continue using the pesticide container for other work, isn't it?	1. Yes<br>2. No
C6.	if yes, in which weather do you not spray?	………………………………………..
C7.	Do you think pesticides can enter our bodies?	1. Yes<br>2. NO
C8.	If yes, in which ways can they enter?	………………………………..
C10.	Do you think the following can be potential pesticide exposure symptoms; Nausea, salivation, blurred vision, skin irritation, vomiting	1. Yes<br>2. No
C11.	Do you think you can reduce on the amount of pesticides you use in agriculture without affecting the expected yield?	1. Yes<br>2. No

D. PRACTICES

D1.	You do post harvest spraying?	1. Yes<br>2. No
D2.	What do you wear during pesticide spraying?	1. Yes<br>2. No
D3.	On average, how long does it take you per spray?	1. <2hrs<br>2. 2-4 hrs

		3. > 4 hrs
D4.	How long does it take you to return to the field after spraying?	1. Hours<br>2. Days<br>3. Weeks<br>4. Months
D5.	Do you follow a particular schedule when spraying?	1. Yes<br>2. No
D6.	What do you use for applying the pesticide?	1. Knap sack sprayer<br>2. Basing on weather condition<br>3. Pail<br>4. Others (Specify):…..
D7.	In case you get a blockage of your sprayer nozzle, what do you do?	1. Use mouth to blow and unblock<br>2. Use a sharp object to unblock<br>3. Replace with another nozzle<br>4. Take to the technician for repair<br>5. Others (Specify):…
D8.	Do you clean or wash your sprayer after use?	1. Yes<br>2. No
D9.	If yes, where do you clean it?	1. House<br>2. Water source<br>3. In the field<br>4. Others (Specify):……
D10.	What is the storage place for the pesticides and the knap sack?	1. House<br>2. Water source<br>3. In the field<br>Others (Specify):……
D11.	Where are the pesticide empty containers kept?	1. Burnt

		2. Re-used<br>3. In the house<br>4. Garden<br>5. Others (specify):…..
D12.	Does the knap sacker leak?	1. Yes<br>2. NO
D13.	Storage within children's access:	1. Far<br>2. Near<br>3. Others (Specify):…..
D14.	Storage in relation to food stores	1. Far<br>2. Near<br>Others (Specify):…..
D15.	Storage in relation to water storage containers	1. Far<br>2. Near<br>Others (Specify):…..
D16.	Are chemicals stored in relation to prescribed storage conditions?	1. Yes<br>2. No
D17.	What is the condition of the PPE?	1. Sound<br>2. Non-sound with holes

Thank you for your participation.
