# Supplementary material for: A Framework for Integrating Qualitative and Quantitative Data in Knowledge, Attitude, and Practice Studies: A Case Study of Pesticide Usage in Eastern Uganda
Source: Front Public Health. 2017 Dec 8;5:318. doi: 10.3389/fpubh.2017.00318 (PMC5727069; doi:10.3389/fpubh.2017.00318)
Supplement: Supplementary file 1 [file Data_Sheet_1.zip › supplementary Material/Supplementary S2.pdf]

KAP supplementary material - R code for quantitative analysis

Introduction

The following document provides R code and explanation in order of the reader to be able to carry out the methodology suggested in our paper using their own data. Here we provide our own dataset called “DB11.csv” as an example.

Load libraries

If you do not have these packages please download them using the function *install.packages(“package name”)*. If any of them is missing then some of the downstream code will not work!!

```
library(knitr)
library(foreign)
library(lme4)
library(boot)
library(parallel)
library(snow)
library(ggplot2)
library(devtools)
library(grid)
library(rms)
library(ResourceSelection)
library(MKmisc)
library(ROCR)
library(pROC)
library(Hmisc)
library(cowplot)
```

Read in Data

Here we read in the database, please note that “DB11.csv” contains 167 rows and 157 columns.

- Columns 1-83 questions from the questionaire
- 84-91 are the question used for the Attitude metric
- 92-109 are the question used for the Practice metric
- 110-120 are the question used for the Knowlege metric

In order to evaluate the contribution (weight) of each question to each of the metric the response were recorded numerically, columns:

- 150-157 are the question used for the Attitude metric(GOOD==1,BAD==2)
- 132-149 are the question used for the Practice metric(PROPER==1,IMPROPER==2)
- 121-131 are the question used for the Knowlege metric(YES==1,NO==2)

```
PESTICIDE_DB<-read.csv("DB11.csv",sep = ",",header = T)
```

Extract KAP questions and metric

```
# The code below subsets questions from which knowledge, attitude and practice
matrices will be generated
KNOWLEDGE_QN<- PESTICIDE_DB[,c(110:120),]
ATTITUTE_QN<- PESTICIDE_DB[,c(84:91),]
PRACTICE_QN<- PESTICIDE_DB[,c(92:109),]

## # The code below subsets numerically coded questions for the knowledge, att
itude and practice matrices which have been used to evaluated the contribution
of each question
KNOWLEDGE_QN1<- PESTICIDE_DB[,c(121:131),]
ATTITUTE_QN1<- PESTICIDE_DB[,c(150:157),]
PRACTICE_QN1<- PESTICIDE_DB[,c(132:149),]

# Here we generate the knowledge matric, which is the proportion of question t
o which the respondent gave a YES out of the 11 total question used to generat
e the Knowledge metric
KNOWLEDGE_QN$Knowledge_Metric <- apply(KNOWLEDGE_QN, 1, function(x)
  length(which(x == "YES" | x == "YES")) / 11)

# Here we generate the Attitude matric, which is the proportion of question fo
r which the response is indicative of GOOD attitude out of the 8 total questio
n used to generate the Attitude metric
ATTITUTE_QN$Attitude_metric <- apply(ATTITUTE_QN, 1, function(x)
  length(which(x == "GOOD" | x == "GOOD")) / 8)

# Here we generate the Pratcice matric, which is the proportion of question fo
r which the response is indicative of PROPER/acceptable practice out of the 18
total question used to generate the Pratcice metric

PRACTICE_QN$Practice_metric <- apply(PRACTICE_QN, 1, function(x)
  length(which(x == "PROPER" | x == "PROPER")) / 18)

# When then add each of the columns with the corresponding metric to original
database for downstream analysis
PESTICIDE_DB$Knowledge_Metric<-KNOWLEDGE_QN$Knowledge_Metric
PESTICIDE_DB$Attitude_metric<-ATTITUTE_QN$Attitude_metric
PESTICIDE_DB$Practice_metric<-PRACTICE_QN$Practice_metric

# Here we generate the binary variable from the knowledge metric which will be
used for logistic regression and univariate regression
PESTICIDE_DB$knowlege_binary<-NA
PESTICIDE_DB$knowlege_binary[PESTICIDE_DB$Knowledge_Metric>=0.50]<-1
PESTICIDE_DB$knowlege_binary[PESTICIDE_DB$Knowledge_Metric<0.50]<-0
```

Data exploration

Here we use the t.test to explore and associations between the binary variable of knowledge and our explanatory variables.

```
t.test(table(PESTICIDE_DB$knowlege_binary,PESTICIDE_DB$N53PROXI))
```

```
##
##  One Sample t-test
##
## data:  table(PESTICIDE_DB$knowlege_binary, PESTICIDE_DB$N53PROXI)
## t = 4.6278, df = 3, p-value = 0.019
## alternative hypothesis: true mean is not equal to 0
## 95 percent confidence interval:
##  12.9613 70.0387
## sample estimates:
## mean of x
##      41.5
```

```
t.test(table(PESTICIDE_DB$knowlege_binary,PESTICIDE_DB$N47DO))
```

```
##
##  One Sample t-test
##
## data:  table(PESTICIDE_DB$knowlege_binary, PESTICIDE_DB$N47DO)
## t = 2.5315, df = 3, p-value = 0.08531
## alternative hypothesis: true mean is not equal to 0
## 95 percent confidence interval:
##  -10.67174  93.67174
## sample estimates:
## mean of x
##      41.5
```

```
t.test(table(PESTICIDE_DB$knowlege_binary,PESTICIDE_DB$N36DO))
```

```
##
##  One Sample t-test
##
## data:  table(PESTICIDE_DB$knowlege_binary, PESTICIDE_DB$N36DO)
## t = 2.17, df = 3, p-value = 0.1185
## alternative hypothesis: true mean is not equal to 0
## 95 percent confidence interval:
##  -19.36299 102.36299
## sample estimates:
## mean of x
##      41.5
```

Visual exploration of the knowledge metric with individual attributes

Here we create a plot which uses the continuous scale of the knowledge metric against some of the respondents attributes, here we use a regression line to give us an indication of the direction of the relationship.

```
# Save each plot as an object
PIA1a<-ggplot(PESTICIDE_DB, aes(x=N5_AGE, y=Knowledge_Metric, fill=N4_SEX, col
our=N4_SEX)) +
  labs(title = "Knowledge by sex", x=" ", y="Knowledge (%)") +
  scale_color_discrete(name="Sex") + theme(legend.title = element_text(size =
7)) +
  geom_point(aes(size=Attitude_metric)) + geom_smooth(method=lm) + theme_bw()
+
  theme(legend.position="bottom", plot.title = element_text(hjust = 0.5)) + gu
ides(fill = "none",  color= "none", size = "none") + scale_size(range = c(1,3)
)

PIA1b<-ggplot(PESTICIDE_DB, aes(x=N5_AGE, y=Knowledge_Metric, fill=N4_SEX, col
our=N4_SEX)) +
  labs(title = "", x="Age in years", y="Knowledge (%)") +
  scale_fill_discrete(name="Sex") + theme(legend.title = element_text(size = 6
)) +
  geom_point(aes(size=Practice_metric)) + geom_smooth(method=lm) + theme_bw()
+
  theme(legend.position="bottom",plot.title = element_text(hjust = 0.5)) + gui
des(color = "none",
  size = "none",text.font=2) + scale_size(range = c(1,3)) +
  theme(legend.title = element_text(size = 9)) + theme(legend.text = element_t
ext(size = 9))+ theme(legend.key.size = unit(0.5, "cm"))


PIA2a<-ggplot(PESTICIDE_DB, aes(x=N5_AGE, y=Knowledge_Metric, fill=N3VILLAG.1,
colour=N3VILLAG.1)) +
  labs(title = "Knowledge by  Residence", x=" ", y=" ") + scale_color_discrete
(name="Res") +
  theme(legend.title = element_text(size = 5)) + geom_point(aes(size=Attitude_
metric)) +
  geom_smooth(method=lm) + theme_bw() + theme(legend.position="bottom") +
  guides(fill = "none", color="none",
         size = "none",text.font=2)+ scale_size(range = c(1,3)) +
  theme(legend.title = element_text(size = 9),plot.title = element_text(hjust
= 0.5)) + theme(legend.text = element_text(size = 9)) + theme(legend.key.size
= unit(0.5, "cm"))


PIA2b<-ggplot(PESTICIDE_DB, aes(x=N5_AGE, y=Knowledge_Metric, fill=N3VILLAG.1,
colour=N3VILLAG.1)) +
  labs(title = "", x="Age in years", y="") +
  theme(legend.title = element_text(size = 5)) +
  geom_point(aes(size=Practice_metric)) + geom_smooth(method=lm) +
  theme_bw() +
  theme(legend.position="bottom") + scale_color_discrete(name="Res") +
  guides(fill = "none",size = "none",text.font=2) + scale_size(range = c(1,3))
+
  theme(legend.title = element_text(size = 9),plot.title = element_text(hjust
= 0.5)) + theme(legend.text = element_text(size = 9)) + theme(legend.key.size
= unit(0.5, "cm"))
```

```
PIA3a<-ggplot(PESTICIDE_DB, aes(x=N5_AGE, y=Knowledge_Metric, fill=N6_MARITAL_
STATUS, colour=N6_MARITAL_STATUS)) +
  labs(title = "", x="Age in years", y="") + scale_color_discrete(name="M Stat
us") +
  theme(legend.title = element_text(size = 5)) + geom_point(aes(size=Practice_
metric)) +
  geom_smooth(method=lm) + theme_bw()  + theme(legend.position="bottom") +
  guides(fill = "none", size = "none",text.font=2) + scale_size(range = c(1,3)
) +
  theme(legend.title = element_text(size = 9),plot.title = element_text(hjust
= 0.5)) + theme(legend.text = element_text(size = 9))+ theme(legend.key.size =
unit(0.5, "cm"))


PIA3b<-ggplot(PESTICIDE_DB, aes(x=N5_AGE, y=Knowledge_Metric, fill=N6_MARITAL_
STATUS, colour=N6_MARITAL_STATUS)) +
  labs(title = "Knowledge by Marital status ", x=" ", y="") + scale_color_disc
rete(name="M status") + theme(legend.title = element_text(size = 5),plot.title
= element_text(hjust = 0.5)) + geom_point(aes(size=Attitude_metric)) +
  geom_smooth(method=lm) + theme_bw() +  theme(legend.position="bottom") +
  guides(fill = "none", color="none",
         size = "none",text.font=2)+ scale_size(range = c(1,3))

PIA5a<-ggplot(PESTICIDE_DB, aes(x=N5_AGE, y=Knowledge_Metric, fill=PESTICIDE_D
B$N8EDUCAT,colour=N8EDUCAT)) +
  labs(title = "Knowledge by Education", x="", y="") + scale_color_discrete(na
me="Educ") +
  theme(legend.title = element_text(size = 5),plot.title = element_text(hjust
= 0.5)) + geom_point(aes(size=Practice_metric)) + theme(legend.position="botto
m") +
  geom_smooth(method=lm)  + theme_bw() +   theme(legend.position="bottom")+ gu
ides(color = "none",fill="none", size = "none",text.font=2)+ scale_size(range
= c(1,3))

PIA5b<-ggplot(PESTICIDE_DB, aes(x=N5_AGE, y=Knowledge_Metric, fill=N8EDUCAT,co
lour=N8EDUCAT)) +
  labs(title = " ", x="Age in years", y=" " ) + scale_color_discrete(name="Edu
c") +
  theme(legend.title = element_text(size = 5))  + geom_point(aes(size=Attitude
_metric)) +    theme(legend.position="bottom") +
  geom_smooth(method=lm)  + theme_bw() + theme(legend.position="bottom")+ guid
es(fill = "none",
  size="none",text.font=2) + scale_size(range = c(1,3)) +
  theme(legend.title = element_text(size = 9)) + theme(legend.text = element_t
ext(size = 9)) + theme(legend.key.size = unit(0.5, "cm"))

# use package plot_grid to show all these figures in one, this comes as part o
f the cowplot package
plot_grid(PIA1a, PIA2a,PIA3b, PIA5a,PIA1b, PIA2b,PIA3a,PIA5b,labels=c("A", "B"
, "C", "D","E","F","G","H"), ncol=4)
```

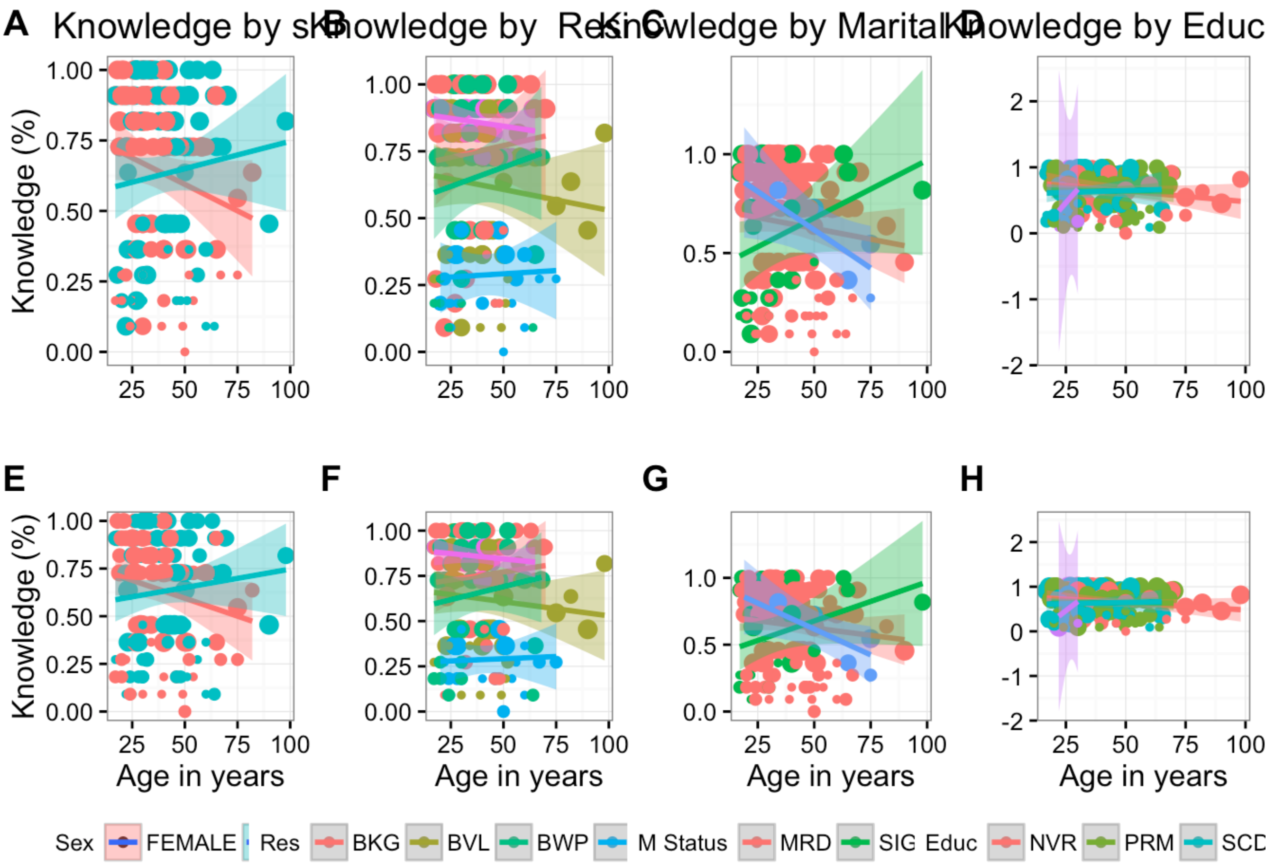

Exploring the relationship between the Practice and Attitude metric

This plot allows us to compare our imperical data with a theorem proposed by Van Doorn J et al 2017, who stated, that there is a threshold beyond which practice has linear relationship to knowlege-attitude. So here we have ploted the practice and attitude metric to explore this theorem. Note that we have used attitude instead of knowledge since these two are correlated.

```
ggplot(PESTICIDE_DB, aes(x=Attitude_metric, y= Practice_metric)) +
  geom_jitter(width=0.015, height=0.015,aes(colour= as.factor(PESTICIDE_DB$N8E
DUCAT),alpha=0.7,shape= as.factor(PESTICIDE_DB$N4_SEX))) +
  geom_vline(xintercept = 0.581, col="grey") + geom_hline(yintercept = 0.5, co
l="grey") +
  scale_color_discrete(name="Education level") +  scale_shape_discrete(name="G
ender") +
  theme(legend.title = element_text(size = 10)) + theme(legend.position="botto
m") + theme_bw() +
  theme(plot.title = element_text(hjust=0.5)) +
  labs( x="Percentage score on Attitude metric", y="Percentage score on Pratic
e metric", title="Relationship between Practice & Attitude")
```

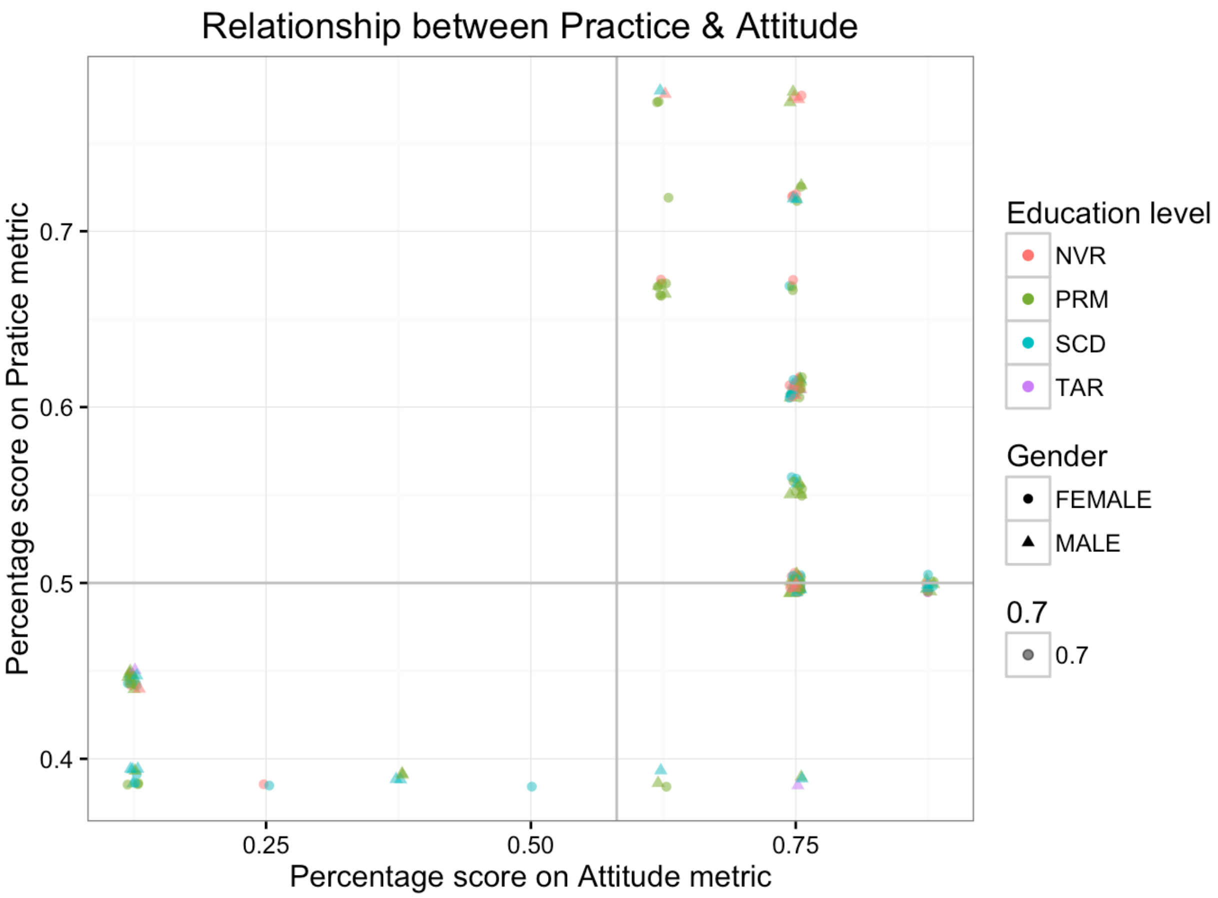

NON PARAMETRIC Principal component analysis for KAP linear relationship

```
### The code for ggbiplot was developed by the Vincent Q Vu and can be freely
downloaded at https://github.com/vqv/ggbiplot
ggbiplot<-function(pcobj, choices = 1:2, scale = 1, pc.biplot = TRUE,
         obs.scale = 1 - scale, var.scale = scale,
         groups = NULL, ellipse = FALSE, ellipse.prob = 0.68,
         labels = NULL, labels.size = 3, alpha = 1,
         var.axes = TRUE,
         circle = FALSE, circle.prob = 0.69,
         varname.size = 3, varname.adjust = 1.5,
         varname.abbrev = FALSE, ...)
{
  library(ggplot2)
  library(plyr)
  library(scales)
  library(grid)

  stopifnot(length(choices) == 2)

  # Recover the SVD
  if(inherits(pcobj, 'prcomp')){
    nobs.factor <- sqrt(nrow(pcobj$x) - 1)
    d <- pcobj$sdev
    u <- sweep(pcobj$x, 2, 1 / (d * nobs.factor), FUN = '*')
    v <- pcobj$rotation
  } else if(inherits(pcobj, 'princomp')) {
    nobs.factor <- sqrt(pcobj$n.obs)
    d <- pcobj$sdev
    u <- sweep(pcobj$scores, 2, 1 / (d * nobs.factor), FUN = '*')
    v <- pcobj$loadings
  } else if(inherits(pcobj, 'PCA')) {
    nobs.factor <- sqrt(nrow(pcobj$call$X))
    d <- unlist(sqrt(pcobj$eig)[1])
    u <- sweep(pcobj$ind$coord, 2, 1 / (d * nobs.factor), FUN = '*')
    v <- sweep(pcobj$var$coord,2,sqrt(pcobj$eig[1:ncol(pcobj$var$coord),1]),FU
N="/")
  } else if(inherits(pcobj, "lda")) {
    nobs.factor <- sqrt(pcobj$N)
    d <- pcobj$svd
    u <- predict(pcobj)$x/nobs.factor
    v <- pcobj$scaling
    d.total <- sum(d^2)
  } else {
    stop('Expected a object of class prcomp, princomp, PCA, or lda')
  }

  # Scores
  choices <- pmin(choices, ncol(u))
  df.u <- as.data.frame(sweep(u[,choices], 2, d[choices]^obs.scale, FUN='*'))

  # Directions
  v <- sweep(v, 2, d^var.scale, FUN='*')
  df.v <- as.data.frame(v[, choices])
```

```
names(df.u) <- c('xvar', 'yvar')
names(df.v) <- names(df.u)

if(pc.biplot) {
  df.u <- df.u * nobs.factor
}

# Scale the radius of the correlation circle so that it corresponds to
# a data ellipse for the standardized PC scores
r <- sqrt(qchisq(circle.prob, df = 2)) * prod(colMeans(df.u^2))^(1/4)

# Scale directions
v.scale <- rowSums(v^2)
df.v <- r * df.v / sqrt(max(v.scale))

# Change the labels for the axes
if(obs.scale == 0) {
  u.axis.labs <- paste('standardized PC', choices, sep='')
} else {
  u.axis.labs <- paste('PC', choices, sep='')
}

# Append the proportion of explained variance to the axis labels
u.axis.labs <- paste(u.axis.labs,
                     sprintf('(%0.1f%% explained var.)',
                             100 * pcobj$sdev[choices]^2/sum(pcobj$sdev^2)))

# Score Labels
if(!is.null(labels)) {
  df.u$labels <- labels
}

# Grouping variable
if(!is.null(groups)) {
  df.u$groups <- groups
}

# Variable Names
if(varname.abbrev) {
  df.v$varname <- abbreviate(rownames(v))
} else {
  df.v$varname <- rownames(v)
}

# Variables for text label placement
df.v$angle <- with(df.v, (180/pi) * atan(yvar / xvar))
df.v$hjust = with(df.v, (1 - varname.adjust * sign(xvar)) / 2)

# Base plot
g <- ggplot(data = df.u, aes(x = xvar, y = yvar)) +
  xlab(u.axis.labs[1]) + ylab(u.axis.labs[2]) + coord_equal()
```

```
if(var.axes) {
  # Draw circle
  if(circle)
  {
    theta <- c(seq(-pi, pi, length = 50), seq(pi, -pi, length = 50))
    circle <- data.frame(xvar = r * cos(theta), yvar = r * sin(theta))
    g <- g + geom_path(data = circle, color = muted('white'),
                       size = 1/2, alpha = 1/3)
  }

  # Draw directions
  g <- g +
    geom_segment(data = df.v,
                 aes(x = 0, y = 0, xend = xvar, yend = yvar),
                 arrow = arrow(length = unit(1/2, 'picas')),
                 color = muted('red'))
}

# Draw either labels or points
if(!is.null(df.u$labels)) {
  if(!is.null(df.u$groups)) {
    g <- g + geom_text(aes(label = labels, color = groups),
                       size = labels.size)
  } else {
    g <- g + geom_text(aes(label = labels), size = labels.size)
  }
} else {
  if(!is.null(df.u$groups)) {
    g <- g + geom_point(aes(color = groups), alpha = alpha)
  } else {
    g <- g + geom_point(alpha = alpha)
  }
}

# Overlay a concentration ellipse if there are groups
if(!is.null(df.u$groups) && ellipse) {
  theta <- c(seq(-pi, pi, length = 50), seq(pi, -pi, length = 50))
  circle <- cbind(cos(theta), sin(theta))

  ell <- ddply(df.u, 'groups', function(x) {
    if(nrow(x) <= 2) {
      return(NULL)
    }
    sigma <- var(cbind(x$xvar, x$yvar))
    mu <- c(mean(x$xvar), mean(x$yvar))
    ed <- sqrt(qchisq(ellipse.prob, df = 2))
    data.frame(sweep(circle %*% chol(sigma) * ed, 2, mu, FUN = '+'),
               groups = x$groups[1])
  })
  names(ell)[1:2] <- c('xvar', 'yvar')
  g <- g + geom_path(data = ell, aes(color = groups, group = groups))
}
```

```
  # Label the variable axes
  if(var.axes) {
    g <- g +
      geom_text(data = df.v,
                aes(label = varname, x = xvar, y = yvar,
                    angle = angle, hjust = hjust),
                color = 'darkred', size = varname.size)
  }
  # Change the name of the legend for groups
  # if(!is.null(groups)) {
  #   g <- g + scale_color_brewer(name = deparse(substitute(groups)),
  #                               palette = 'Dark2')
  # }

  # TODO: Add a second set of axes

  return(g)
}

## Here we convert all the variables to be used in this principal component an
alysis to log for scaling purposes
PESTICIDE_DB$Knowledge<-log(PESTICIDE_DB$Knowledge_Metric)
PESTICIDE_DB$Attitude<-log(PESTICIDE_DB$Attitude_metric)
PESTICIDE_DB$Practice<-log(PESTICIDE_DB$Practice_metric)
PESTICIDE_DB$Experience<-log(PESTICIDE_DB$N11HOW)
PESTICIDE_DB$AGE<-log(PESTICIDE_DB$N5_AGE)
PESTICIDE_DB$SEX<-NA
PESTICIDE_DB$SEX[PESTICIDE_DB$N4_SEX=="MALE"]<-1
PESTICIDE_DB$SEX[PESTICIDE_DB$N4_SEX=="FEMALE"]<-2
PESTICIDE_DB$Gender<-log(PESTICIDE_DB$SEX)

PESTICIDE_DBPC<- PESTICIDE_DB[-155,] # removes a row with an infinite outcome
for the knowldge metric

KAP.pca_2 <- prcomp(PESTICIDE_DBPC[,c(162:164,166,168),],
                    center = TRUE,
                    scale. = TRUE)

print(KAP.pca_2)
```

```
## Standard deviations:
## [1] 1.4943215 1.0502814 0.9627093 0.7170930 0.4721024
##
## Rotation:
##                   PC1         PC2        PC3        PC4         PC5
## Knowledge -0.58967221 -0.03715822 -0.1581479 -0.4513610  0.64974493
## Attitude  -0.60861516 -0.04595541 -0.1533739 -0.2202232 -0.74528779
## Practice  -0.52338316  0.09490830  0.1938289  0.8120403  0.14171572
## AGE       -0.05890921  0.74727668  0.6061113 -0.2620835 -0.04526196
## Gender    -0.06688837 -0.65503995  0.7392734 -0.1402774 -0.01567319
```

```
summary(KAP.pca_2)
```

```
## Importance of components:
##                           PC1    PC2    PC3    PC4     PC5
## Standard deviation     1.4943 1.0503 0.9627 0.7171 0.47210
## Proportion of Variance 0.4466 0.2206 0.1854 0.1028 0.04458
## Cumulative Proportion  0.4466 0.6672 0.8526 0.9554 1.00000
```

```
screeplot(KAP.pca_2,type="lines",col=3)
```

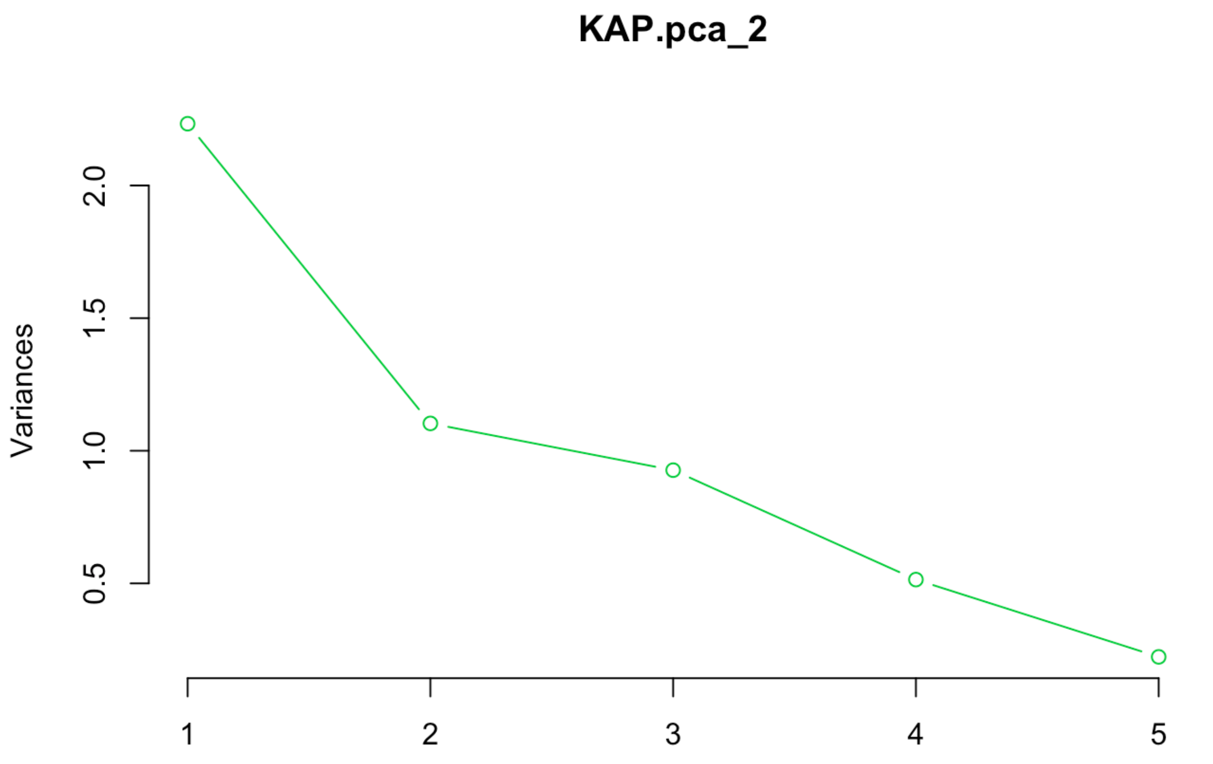


```
g_1 <- ggbiplot(KAP.pca_2, obs.scale = 1, var.scale = 1,
                groups = PESTICIDE_DBPC$N3VILLAG, ellipse = TRUE,
                circle = TRUE)
g_1 <- g_1 + scale_color_discrete(name = '')
g_1 <- g_1 + theme(legend.direction = 'horizontal',
                   legend.position = 'top') + theme_bw()
print(g_1)
```

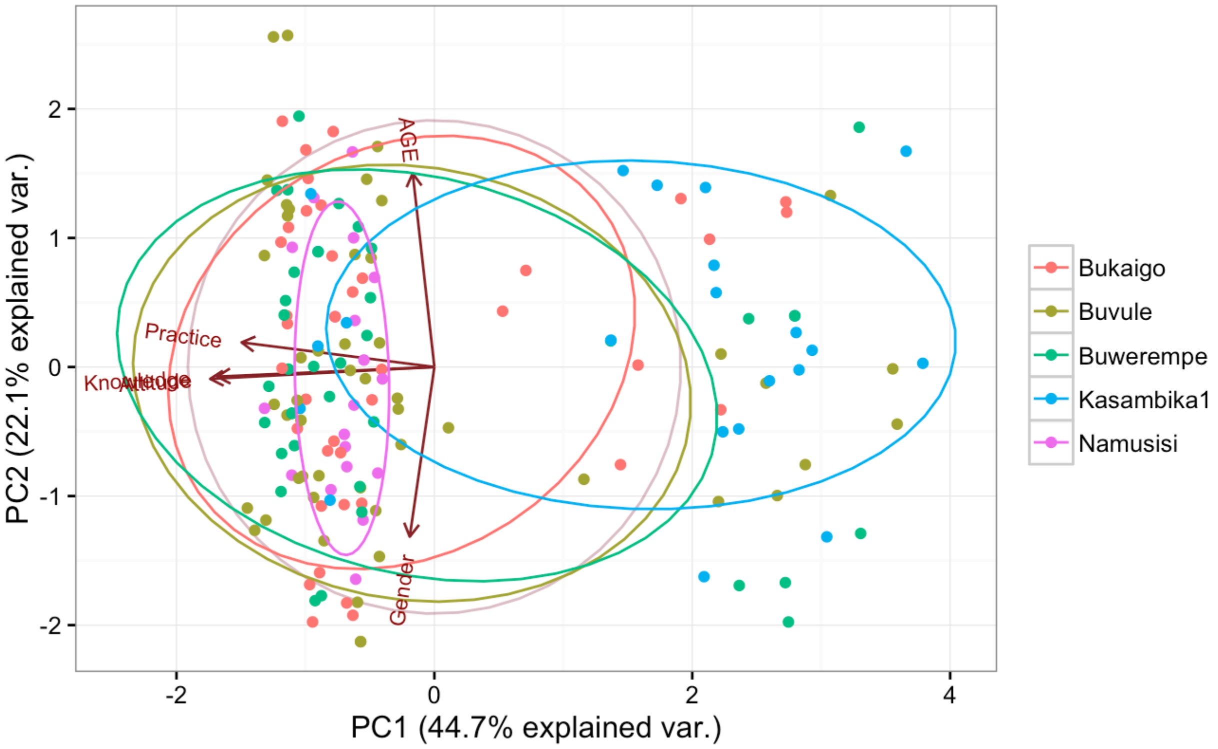

Principal component analysis: When data is split into low and high knowledge score

In the biplot above you can see that there are two populations spread along PC1, we though that this could have an effect on the linearity of the KAP metrics, so we divided the sample into those who scored high and low on knowldge metric to see if the KAP axiom holds. Below is the analysis with the the group that score low on the knowledge metric.

```
PESTICIDE_DBLOW<-PESTICIDE_DBPC[PESTICIDE_DBPC$Knowledge_Metric<=0.5,]
PESTICIDE_DBHIGH<-PESTICIDE_DBPC[PESTICIDE_DBPC$Knowledge_Metric>=0.5,]

KAP.pca_lowscore <- prcomp(PESTICIDE_DBLOW[,c(162:164,166,168),],
                           center = TRUE,
                           scale. = TRUE)

print(KAP.pca_lowscore)
```

```
## Standard deviations:
## [1] 1.4862985 1.0096148 0.9980412 0.6697181 0.5718270
##
## Rotation:
##                 PC1         PC2         PC3         PC4        PC5
## Knowledge 0.5598110 -0.08380318 -0.06835079  0.78659497 -0.2370341
## Attitude  0.5590189  0.08092486 -0.24561717 -0.57336863 -0.5402496
## Practice  0.5933834  0.07386176  0.06552265 -0.17378257  0.7797095
## AGE       0.1441984 -0.49892670  0.82861326 -0.13248577 -0.1616371
## Gender    0.0348196  0.85559616  0.49407209  0.06902103 -0.1336850
```

```
summary(KAP.pca_lowscore)
```

```
## Importance of components:
##                           PC1    PC2    PC3    PC4    PC5
## Standard deviation     1.4863 1.0096 0.9980 0.6697 0.5718
## Proportion of Variance 0.4418 0.2039 0.1992 0.0897 0.0654
## Cumulative Proportion  0.4418 0.6457 0.8449 0.9346 1.0000
```

```
screeplot(KAP.pca_lowscore,type="lines",col=3)
```

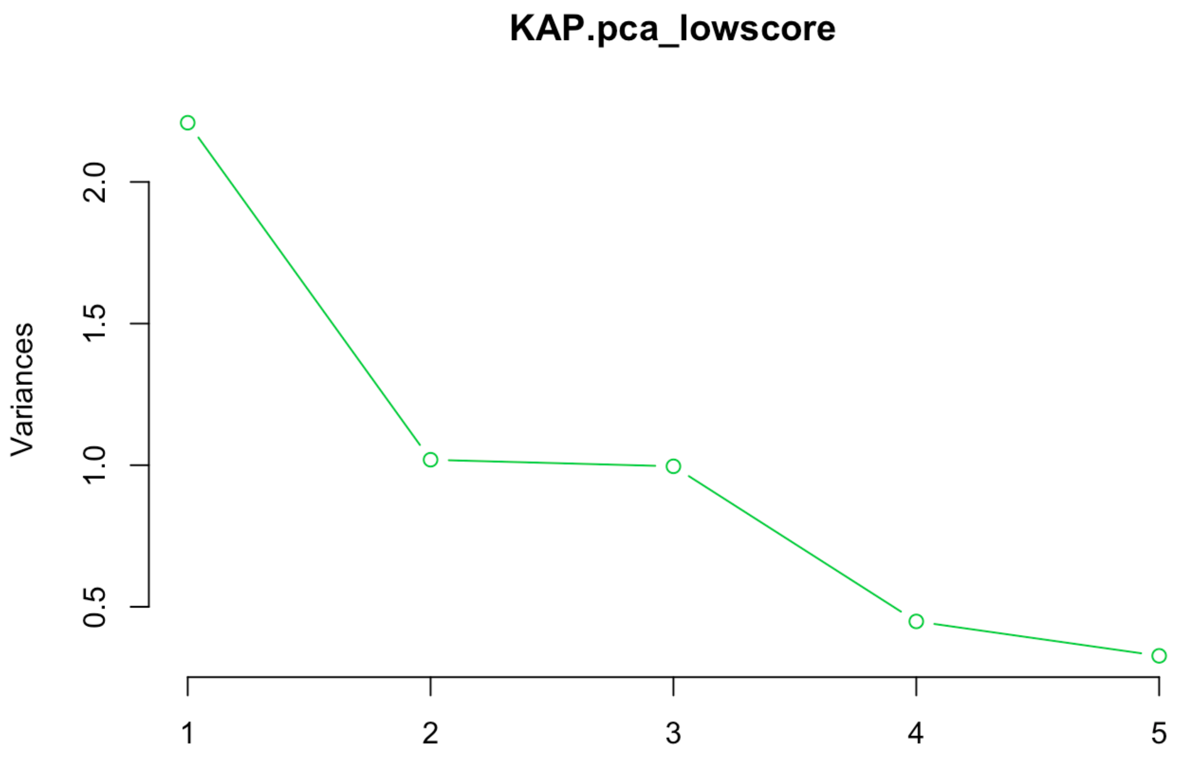

```
g_lowscore <- ggbiplot(KAP.pca_lowscore, obs.scale = 1, var.scale = 1,
                       groups = PESTICIDE_DBLOW$N3VILLAG, ellipse = TRUE,
                       circle = TRUE)
g_lowscore <- g_lowscore + scale_color_discrete(name = '')
g_lowscore <- g_lowscore + theme(legend.direction = 'horizontal',
                                 legend.position = 'top') + theme_bw()
print(g_lowscore)
```

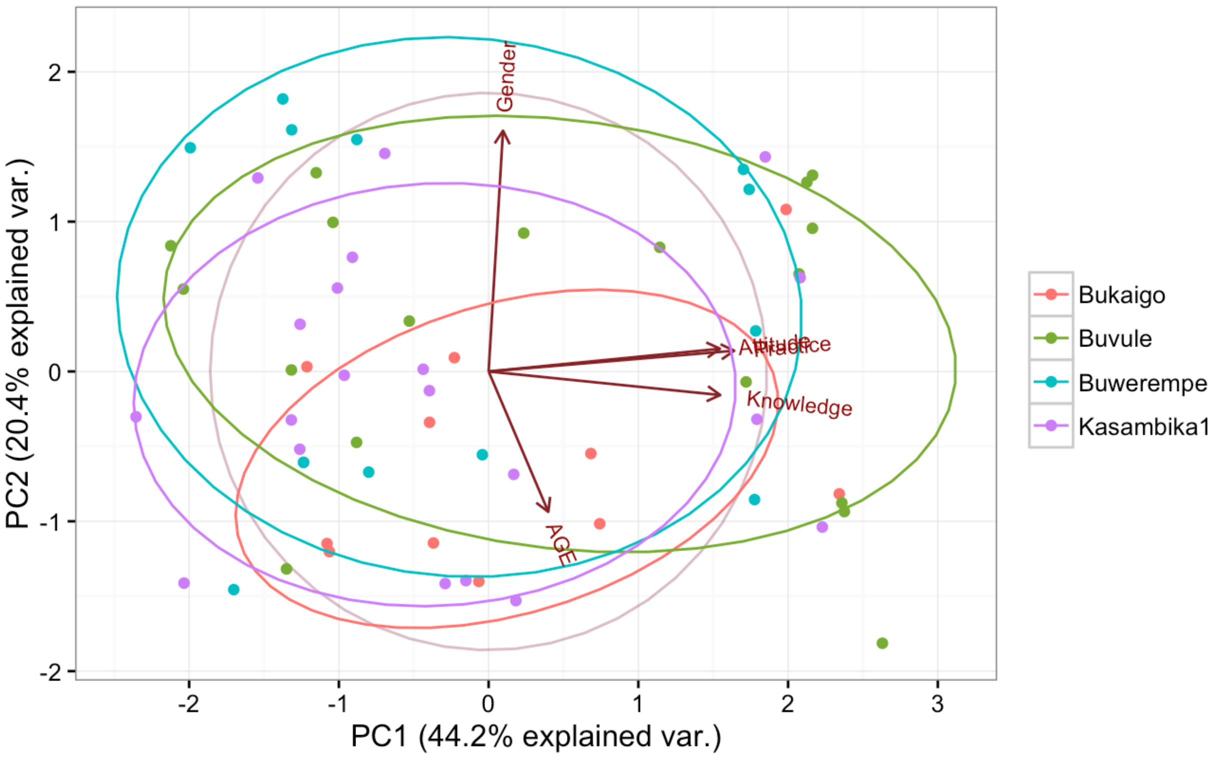


```
## Here is the analysis for the group that score high on the knowledge metric

KAP.pca_highscore <- prcomp(PESTICIDE_DBHIGH[,c(162:164,166,168),],
                            center = TRUE,
                            scale. = TRUE)

print(KAP.pca_highscore)
```

```
## Standard deviations:
## [1] 1.4272010 1.0693251 0.9260639 0.7930930 0.5771052
##
## Rotation:
##                   PC1           PC2        PC3        PC4          PC5
## Knowledge -0.50004617  0.0002919966 -0.3134344  0.7999378  0.108683775
## Attitude  -0.59425108 -0.1271512873  0.2918169 -0.1590872 -0.721271329
## Practice   0.58980495  0.0216239897 -0.2619102  0.3577170 -0.674614063
## AGE        0.20998373 -0.6642403404  0.6124233  0.3560681  0.113335368
## Gender     0.06968815  0.7363081136  0.6106903  0.2829220 -0.002543151
```

```
summary(KAP.pca_highscore)
```

```
## Importance of components:
##                           PC1    PC2    PC3    PC4     PC5
## Standard deviation     1.4272 1.0693 0.9261 0.7931 0.57711
## Proportion of Variance 0.4074 0.2287 0.1715 0.1258 0.06661
## Cumulative Proportion  0.4074 0.6361 0.8076 0.9334 1.00000
```

```
screeplot(KAP.pca_highscore,type="lines",col=3)
```

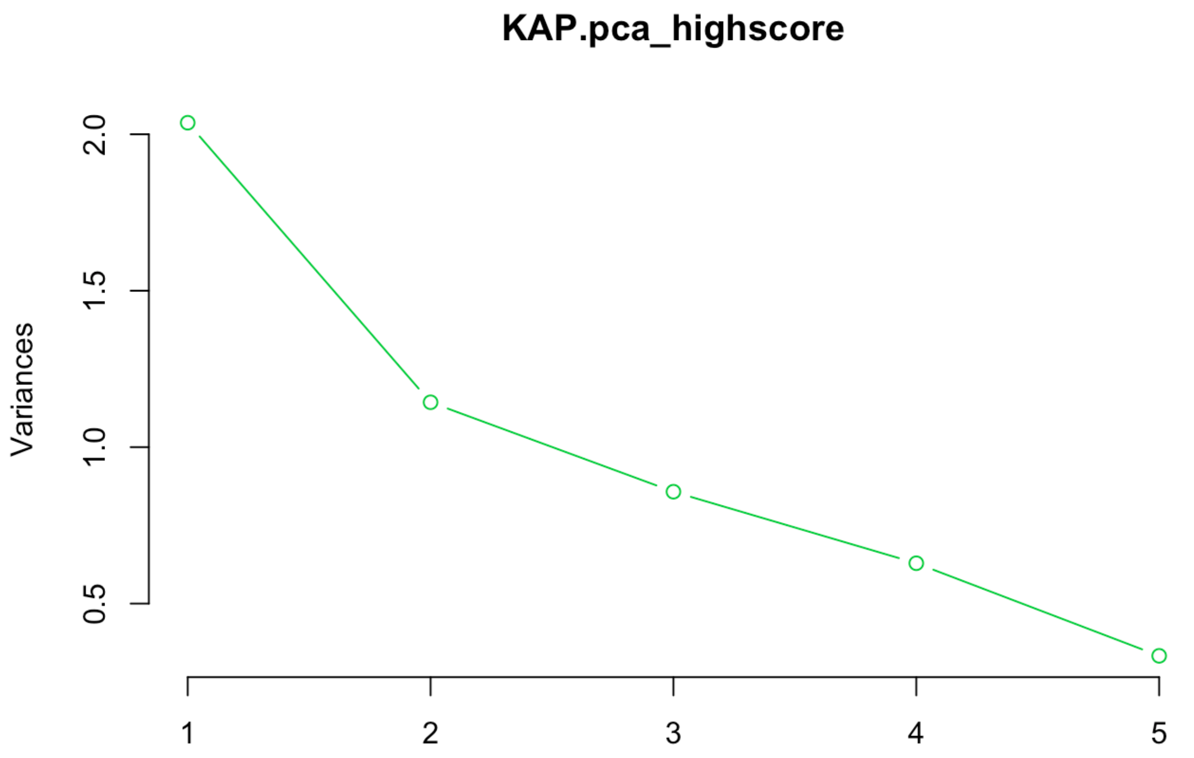

```
g_highscore <- ggbiplot(KAP.pca_highscore, obs.scale = 1, var.scale = 1,
                        groups = PESTICIDE_DBHIGH$N3VILLAG, ellipse = TRUE,
                        circle = TRUE)
g_highscore <- g_highscore + scale_color_discrete(name = '')
g_highscore <- g_highscore + theme(legend.direction = 'horizontal',
                                   legend.position = 'top') + theme_bw()
print(g_highscore)
```

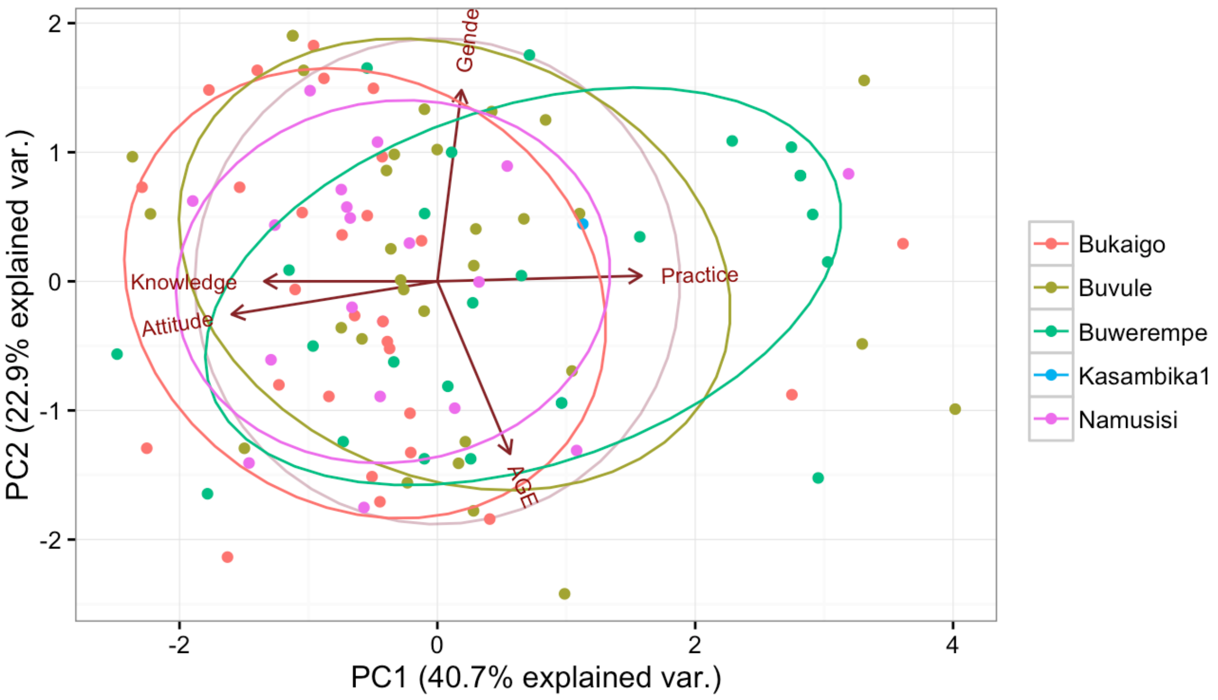

PARAMETRIC ANALYSIS Evaluating the linear relationship using Pearson’s correlation coefficient

Low score on knowledge metric

Here would like to see if the same is true if we used a parametric analysis, so we run the knowledge metrics for the group that had a low score and the attitude and practices using the pearson correlation coefficient. A positive correlation suggests a linear relation ship and negative suggests the opposite.

```
cor.test(PESTICIDE_DBLOW$Knowledge_Metric,PESTICIDE_DBLOW$Attitude_metric)
```

```
##
##  Pearson's product-moment correlation
##
## data:  PESTICIDE_DBLOW$Knowledge_Metric and PESTICIDE_DBLOW$Attitude_metric
## t = 5.9772, df = 60, p-value = 1.341e-07
## alternative hypothesis: true correlation is not equal to 0
## 95 percent confidence interval:
##  0.4261791 0.7467421
## sample estimates:
##       cor
## 0.6109176
```

```
cor.test(PESTICIDE_DBLOW$Knowledge_Metric,PESTICIDE_DBLOW$Practice_metric)
```

```
##
##  Pearson's product-moment correlation
##
## data:  PESTICIDE_DBLOW$Knowledge_Metric and PESTICIDE_DBLOW$Practice_metric
## t = 6.8547, df = 60, p-value = 4.425e-09
## alternative hypothesis: true correlation is not equal to 0
## 95 percent confidence interval:
##  0.4948473 0.7828889
## sample estimates:
##       cor
## 0.6627069
```

```
cor.test(PESTICIDE_DBLOW$Attitude_metric,PESTICIDE_DBLOW$Practice_metric)
```

```
##
##  Pearson's product-moment correlation
##
## data:  PESTICIDE_DBLOW$Attitude_metric and PESTICIDE_DBLOW$Practice_metric
## t = 7.4575, df = 60, p-value = 4.14e-10
## alternative hypothesis: true correlation is not equal to 0
## 95 percent confidence interval:
##  0.5367820 0.8040466
## sample estimates:
##       cor
## 0.6935634
```

High score on knowledge metric

This is the same analysis as above on the group that had a high score on the knowledge metric, please read the article for to get the interpretation.

```
cor.test(PESTICIDE_DBHIGH$Knowledge_Metric,PESTICIDE_DBHIGH$Attitude_metric)
```

```
##
##  Pearson's product-moment correlation
##
## data:  PESTICIDE_DBHIGH$Knowledge_Metric and PESTICIDE_DBHIGH$Attitude_metr
ic
## t = 4.4724, df = 102, p-value = 2.013e-05
## alternative hypothesis: true correlation is not equal to 0
## 95 percent confidence interval:
##  0.2302799 0.5542759
## sample estimates:
##       cor
## 0.4049107
```

```
cor.test(PESTICIDE_DBHIGH$Knowledge_Metric,PESTICIDE_DBHIGH$Practice_metric)
```

```
##
##  Pearson's product-moment correlation
##
## data:  PESTICIDE_DBHIGH$Knowledge_Metric and PESTICIDE_DBHIGH$Practice_metr
ic
## t = -4.1324, df = 102, p-value = 7.372e-05
## alternative hypothesis: true correlation is not equal to 0
## 95 percent confidence interval:
##  -0.5324493 -0.2007463
## sample estimates:
##        cor
## -0.3786937
```

```
cor.test(PESTICIDE_DBHIGH$Attitude_metric,PESTICIDE_DBHIGH$Practice_metric)
```

```
##
##  Pearson's product-moment correlation
##
## data:  PESTICIDE_DBHIGH$Attitude_metric and PESTICIDE_DBHIGH$Practice_metri
c
## t = -8.5994, df = 102, p-value = 1.007e-13
## alternative hypothesis: true correlation is not equal to 0
## 95 percent confidence interval:
##  -0.7475488 -0.5207208
## sample estimates:
##       cor
## -0.648295
```

Evaluating the weight of questions included in a questionnaire

Attitude questions

This analysis aims at evaluating the weight each question brings to the corresponding metric. We have used a PCA and we taking the correlation coefficient for each question in the PC1 (which explains the largest variation) as the weight.

```
ATTITUTE_QN1<-ATTITUTE_QN1[,-2]
KAP.pca_Att <- prcomp(ATTITUTE_QN1,
                      center = TRUE,
                      scale. = TRUE)

screeplot(KAP.pca_Att,type="lines",col=3)
```

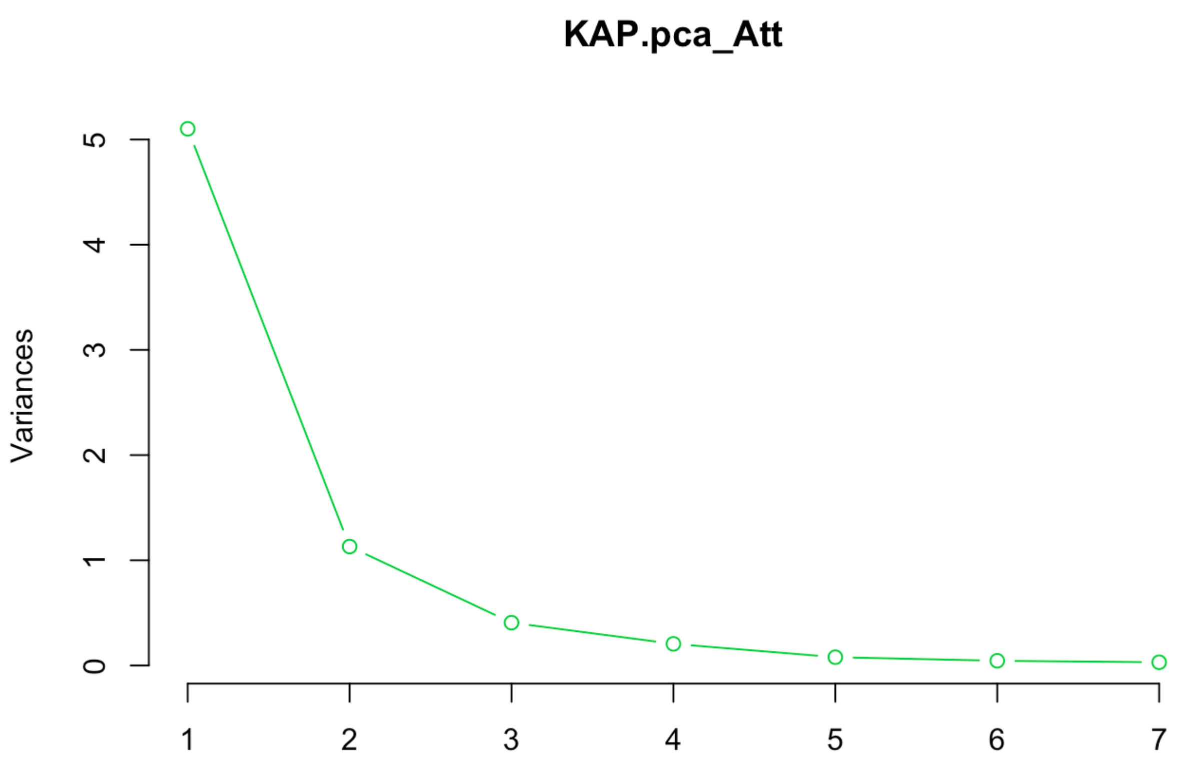


```
summary(KAP.pca_Att)
```

```
## Importance of components:
##                           PC1    PC2     PC3     PC4     PC5     PC6
## Standard deviation     2.2587 1.0632 0.63814 0.45354 0.28095 0.21209
## Proportion of Variance 0.7288 0.1615 0.05817 0.02939 0.01128 0.00643
## Cumulative Proportion  0.7288 0.8903 0.94850 0.97789 0.98917 0.99559
##                            PC7
## Standard deviation     0.17566
## Proportion of Variance 0.00441
## Cumulative Proportion  1.00000
```

```
print(KAP.pca_Att)
```

```
## Standard deviations:
## [1] 2.2587211 1.0632461 0.6381355 0.4535430 0.2809490 0.2120871 0.1756564
##
## Rotation:
##                         PC1          PC2         PC3        PC4
## ReCoded_N30DO     0.4126221  0.076337501 -0.35266369  0.5328275
## ReCoded_N32MIXIN -0.3403354  0.323779426 -0.80813767 -0.3400460
## ReCoded_N33DO     0.4228886  0.074070425 -0.29827104  0.3490422
## ReCoded_N35DO     0.4224837  0.003741583  0.10416918 -0.4279425
## ReCoded_N36DO     0.4315658  0.060451966 -0.08956715 -0.2437587
## ReCoded_N37AFTER  0.1611698 -0.843930484 -0.31944755 -0.2678525
## ReCoded_N38DO     0.3799513  0.409836480  0.11249878 -0.4053826
##                           PC5        PC6          PC7
## ReCoded_N30DO    -0.048880716  0.4457038  0.463246758
## ReCoded_N32MIXIN -0.100351011  0.0225254 -0.006576403
## ReCoded_N33DO    -0.001769396 -0.3217010 -0.708087644
## ReCoded_N35DO    -0.751683761  0.2274821 -0.103590527
## ReCoded_N36DO     0.089501546 -0.6974090  0.498263057
## ReCoded_N37AFTER  0.248439978  0.1511190 -0.058775236
## ReCoded_N38DO     0.593949319  0.3693031 -0.146695202
```

Practice questions

```
PRACTICE_QN1<-PRACTICE_QN1[,-c(2,6),]# removed columes with NA
 KAP.pca_prac <- prcomp(PRACTICE_QN1,
                        center = TRUE,
                       scale. = TRUE)

 screeplot(KAP.pca_prac,type="lines",col=3)
```

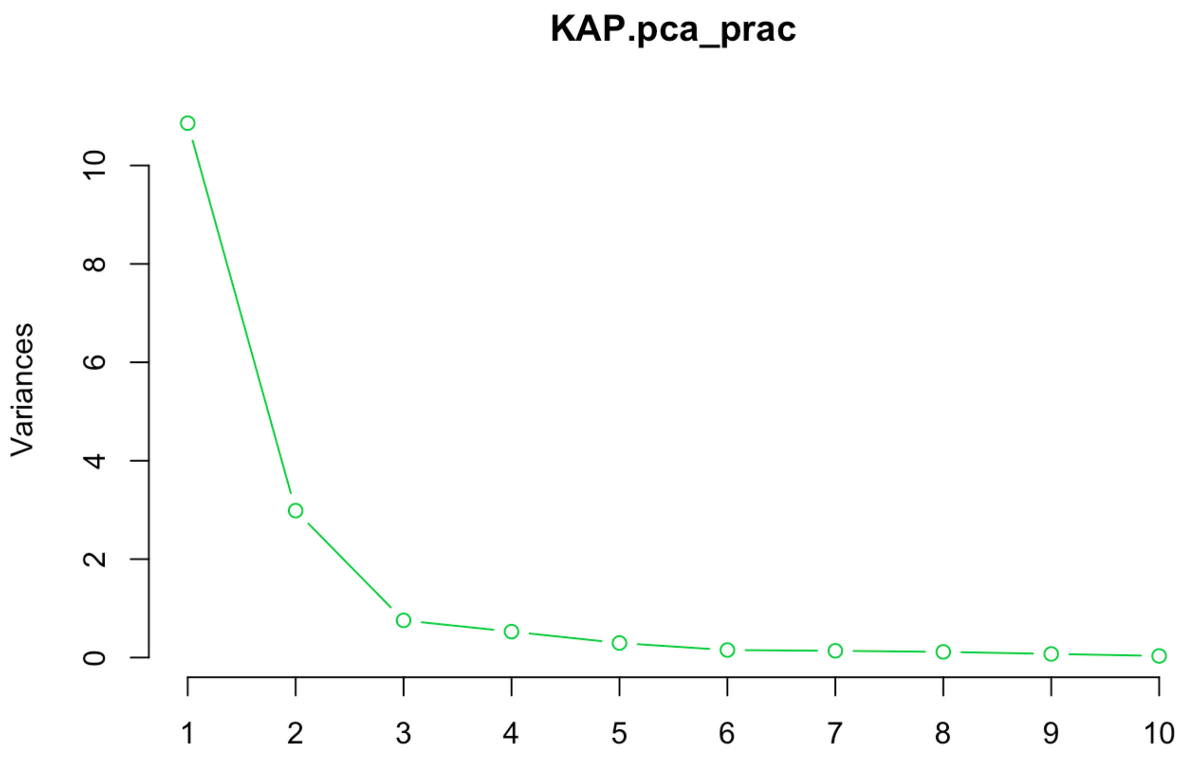


```
print(KAP.pca_prac)
```

```
## Standard deviations:
##  [1] 3.295592e+00 1.727973e+00 8.690084e-01 7.257374e-01 5.432658e-01
##  [6] 3.902441e-01 3.702052e-01 3.391939e-01 2.706090e-01 1.802173e-01
## [11] 1.368119e-01 1.150142e-01 1.094476e-01 1.078571e-01 1.025475e-01
## [16] 1.737463e-16
##
## Rotation:
##                          PC1          PC2          PC3         PC4
## ReCoded_N39DO    -0.2640956 -0.190254066  0.103409098 -0.09378346
## ReCoded_N42ON     0.2566397  0.280523682  0.133235281 -0.05418094
## ReCoded_N43HOW   -0.1621634  0.243880340 -0.503263939 -0.80833407
## ReCoded_N44DO     0.2526442 -0.282943672  0.021982087 -0.19798866
## ReCoded_N46IN    -0.2649780 -0.005875491  0.403935178 -0.21759403
## ReCoded_N47DO     0.2465466 -0.306926016  0.133509490 -0.20794756
## ReCoded_N48IF    -0.2649790  0.097982345  0.449898987 -0.19034558
## ReCoded_N49LOCAT -0.2645922  0.107834157  0.440076533 -0.17869645
## ReCoded_N50WHERE  0.2623812  0.252931561  0.037893875  0.01362465
## ReCoded_N51HOW    0.2535798 -0.274542670 -0.005826879 -0.18476357
## ReCoded_N52DOES  -0.2505147 -0.285678270 -0.168033445  0.08144654
## ReCoded_N53PROXI -0.2549804 -0.283840630 -0.148146097  0.06535515
## ReCoded_N54PROXI -0.2312334 -0.275614929 -0.198724467  0.11085960
## ReCoded_N55REACH  0.2444080 -0.310116219  0.156950061 -0.19837257
## ReCoded_N56STORA  0.2444080 -0.310116219  0.156950061 -0.19837257
## ReCoded_N58WHAT   0.2630273  0.245509490  0.017938433  0.02628992
##                            PC5         PC6           PC7          PC8
## ReCoded_N39DO    -0.467903753  0.16548731 -0.2972482157 -0.193547436
## ReCoded_N42ON    -0.046436521  0.16445793 -0.2598258180 -0.242619076
## ReCoded_N43HOW    0.043678872 -0.06156072 -0.0414404432  0.008946593
## ReCoded_N44DO    -0.007415807  0.37570807  0.3450130465 -0.119451301
## ReCoded_N46IN    -0.038739713  0.45262086 -0.2351401104  0.628520236
## ReCoded_N47DO     0.062322675 -0.18636755 -0.1335604741  0.030577856
## ReCoded_N48IF     0.233530436 -0.10524043  0.2153440157 -0.235879291
## ReCoded_N49LOCAT  0.241715044 -0.14494042  0.2221929011 -0.272980767
## ReCoded_N50WHERE  0.336822609  0.12003237 -0.0506036044  0.181695445
## ReCoded_N51HOW   -0.023557228  0.44016968  0.3877986298 -0.125633175
## ReCoded_N52DOES   0.248990493 -0.05647594  0.1421390965  0.212434929
## ReCoded_N53PROXI  0.120539940 -0.14540741  0.2531392014  0.274244423
## ReCoded_N54PROXI  0.556831340  0.33962042 -0.4626673134 -0.368481841
## ReCoded_N55REACH  0.073839784 -0.29387263 -0.2271762775  0.061154288
## ReCoded_N56STORA  0.073839784 -0.29387263 -0.2271762775  0.061154288
## ReCoded_N58WHAT   0.383540162  0.09157097 -0.0002876316  0.225393867
##                             PC9          PC10          PC11          PC12
## ReCoded_N39DO     0.7050125967 -4.128228e-02  6.114795e-05 -1.963471e-04
## ReCoded_N42ON    -0.0690047758  5.426250e-01 -8.491259e-07 -5.460106e-06
## ReCoded_N43HOW   -0.0002205906  2.136345e-07  7.150670e-03  8.695835e-04
## ReCoded_N44DO     0.0083620174 -2.953236e-05 -1.141874e-01  7.224731e-01
## ReCoded_N46IN    -0.2471446374  3.906730e-03 -1.187396e-03  5.847543e-03
## ReCoded_N47DO    -0.0004868588 -5.300335e-06 -8.209284e-01 -2.285789e-01
## ReCoded_N48IF     0.0404056085 -1.268303e-04  1.363467e-02 -9.897727e-02
## ReCoded_N49LOCAT  0.0559704044 -4.406191e-04 -3.045603e-03  7.722440e-02
## ReCoded_N50WHERE  0.4438029279 -5.912433e-02  1.058780e-05 -6.972048e-05
## ReCoded_N51HOW    0.0071885560 -8.985929e-06  2.339669e-01 -6.341968e-01
```

```
## ReCoded_N52DOES   0.2035750976  7.775703e-01 -1.316393e-06  6.826500e-06
## ReCoded_N53PROXI  0.1490527215 -2.294273e-01  1.998286e-06 -1.842411e-05
## ReCoded_N54PROXI -0.1326498730 -1.605919e-01  1.429133e-07 -5.060290e-07
## ReCoded_N55REACH -0.0025701512  6.040158e-06  3.592002e-01  6.238425e-02
## ReCoded_N56STORA -0.0025701512  6.040158e-06  3.592002e-01  6.238425e-02
## ReCoded_N58WHAT   0.3924259813 -1.315311e-01 -1.372033e-06  5.003219e-05
##                          PC13          PC14          PC15          PC16
## ReCoded_N39DO    -1.036916e-03 -5.713060e-02  2.046981e-02 -2.005259e-17
## ReCoded_N42ON    -3.105274e-04  2.047470e-01  5.814241e-01  3.769177e-17
## ReCoded_N43HOW   -5.924892e-05 -7.073999e-08  1.326567e-09  4.035117e-17
## ReCoded_N44DO    -8.270957e-02 -1.137801e-04  3.381114e-06 -2.049116e-16
## ReCoded_N46IN     3.757430e-02  1.717935e-03 -5.338047e-04  9.653998e-17
## ReCoded_N47DO     1.954120e-02  2.493065e-05 -5.864411e-07  7.035709e-16
## ReCoded_N48IF    -7.087340e-01 -1.405818e-03  1.058855e-04 -1.000650e-16
## ReCoded_N49LOCAT  6.930609e-01  1.294298e-03 -8.041482e-05  3.013350e-17
## ReCoded_N50WHERE -1.404830e-03  6.345313e-01 -3.147067e-01 -6.194482e-17
## ReCoded_N51HOW    9.315845e-02  1.368269e-04 -5.000569e-06  1.054817e-16
## ReCoded_N52DOES   1.890609e-04 -1.053711e-01 -1.818848e-01 -4.256448e-17
## ReCoded_N53PROXI -5.894229e-04  3.423782e-01  6.790887e-01 -2.113945e-17
## ReCoded_N54PROXI -1.259369e-05  6.802042e-03  1.054175e-02  8.952886e-17
## ReCoded_N55REACH -4.703774e-03 -5.786264e-06  1.202605e-07  7.071068e-01
## ReCoded_N56STORA -4.703774e-03 -5.786264e-06  1.202605e-07 -7.071068e-01
## ReCoded_N58WHAT   1.325747e-03 -6.510038e-01  2.610204e-01  2.565328e-17
```

```
summary(KAP.pca_prac)
```

```
## Importance of components:
##                           PC1    PC2    PC3     PC4     PC5     PC6
## Standard deviation     3.2956 1.7280 0.8690 0.72574 0.54327 0.39024
## Proportion of Variance 0.6788 0.1866 0.0472 0.03292 0.01845 0.00952
## Cumulative Proportion  0.6788 0.8654 0.9126 0.94554 0.96399 0.97351
##                            PC7     PC8     PC9    PC10    PC11    PC12
## Standard deviation     0.37021 0.33919 0.27061 0.18022 0.13681 0.11501
## Proportion of Variance 0.00857 0.00719 0.00458 0.00203 0.00117 0.00083
## Cumulative Proportion  0.98207 0.98926 0.99384 0.99587 0.99704 0.99787
##                           PC13    PC14    PC15      PC16
## Standard deviation     0.10945 0.10786 0.10255 1.737e-16
## Proportion of Variance 0.00075 0.00073 0.00066 0.000e+00
## Cumulative Proportion  0.99862 0.99934 1.00000 1.000e+00
```

Knowledge questions

```
KAP.pca_know <- prcomp(KNOWLEDGE_QN1,
                       center = TRUE,
                       scale. = TRUE)

print(KAP.pca_know)
```

```
## Standard deviations:
##  [1] 2.1919002 1.2310601 1.0817902 0.9162944 0.8231440 0.7590523 0.6299403
##  [8] 0.6078018 0.5891423 0.4427993 0.3272073
##
## Rotation:
##                                           PC1         PC2         PC3
## ReCoded_Mixingpesticides          -0.2772244  0.07471687  0.38159525
## ReCoded_Alternativetopesticides   -0.3874795 -0.17278065 -0.01201387
## ReCoded_Trainingonpesticdeuse     -0.3575161 -0.29646031 -0.10283461
## ReCoded_KnowledgeonPPE            -0.2452812  0.31787483 -0.47417258
## ReCoded_FarmerswearPPE            -0.2949953  0.46010033 -0.03586822
## ReCoded_SpiltPesticides           -0.4260238 -0.03347768  0.13220746
## ReCoded_Knowledgeonmarks          -0.3721320 -0.18338343  0.11794089
## ReCoded_trainingonsafePecdehandlg -0.2529991 -0.46196200 -0.14250318
## ReCoded_Pesticideexposuresymptoms -0.2017756  0.22050221 -0.59819159
## ReCoded_COntainers                -0.2484850  0.16385466  0.35555998
## ReCoded_Effectonhealth            -0.1075092  0.49021433  0.28563530
##                                           PC4         PC5          PC6
## ReCoded_Mixingpesticides          -0.27325231  0.49624869 -0.527797492
## ReCoded_Alternativetopesticides    0.01877308  0.15433617  0.308811404
## ReCoded_Trainingonpesticdeuse      0.19542188 -0.03608142  0.036193320
## ReCoded_KnowledgeonPPE            -0.19838994  0.26837624  0.416771602
## ReCoded_FarmerswearPPE             0.10263355  0.13048241  0.146684907
## ReCoded_SpiltPesticides           -0.05717022  0.04353041 -0.006212966
## ReCoded_Knowledgeonmarks          -0.10606622 -0.07217167 -0.093284930
## ReCoded_trainingonsafePecdehandlg  0.42766646 -0.11105732 -0.049415844
## ReCoded_Pesticideexposuresymptoms -0.06857584 -0.33751185 -0.612059744
## ReCoded_COntainers                -0.36164295 -0.70696098  0.197331996
## ReCoded_Effectonhealth             0.70992498 -0.08011740 -0.069286975
##                                           PC7          PC8         PC9
## ReCoded_Mixingpesticides           0.36163415 -0.135973544  0.05078222
## ReCoded_Alternativetopesticides   -0.06827782  0.130556940  0.37856456
## ReCoded_Trainingonpesticdeuse      0.20410527  0.378731865  0.45107125
## ReCoded_KnowledgeonPPE             0.12963440 -0.507627765  0.05232511
## ReCoded_FarmerswearPPE             0.05554218  0.583445029 -0.52794814
## ReCoded_SpiltPesticides           -0.21672269 -0.001951938 -0.10248642
## ReCoded_Knowledgeonmarks          -0.70314683 -0.201315405 -0.18533800
## ReCoded_trainingonsafePecdehandlg  0.39037715 -0.304809874 -0.49124879
## ReCoded_Pesticideexposuresymptoms -0.04306544  0.054882877  0.12829870
## ReCoded_COntainers                 0.31563756 -0.097749440  0.01705138
## ReCoded_Effectonhealth            -0.08803238 -0.277356018  0.25738548
##                                           PC10        PC11
## ReCoded_Mixingpesticides           0.013606448  0.14288901
## ReCoded_Alternativetopesticides    0.724104422  0.10478685
## ReCoded_Trainingonpesticdeuse     -0.576943807  0.10719508
## ReCoded_KnowledgeonPPE            -0.223003919  0.04370898
## ReCoded_FarmerswearPPE             0.009885230  0.16912479
## ReCoded_SpiltPesticides           -0.081727442 -0.85472034
## ReCoded_Knowledgeonmarks          -0.178798593  0.43307610
## ReCoded_trainingonsafePecdehandlg  0.128986503  0.03653583
## ReCoded_Pesticideexposuresymptoms  0.193028819 -0.03179217
## ReCoded_COntainers                 0.015425240  0.07585450
```

```
## ReCoded_Effectonhealth            -0.003337038  0.01991757
```

```
summary(KAP.pca_know)
```

```
## Importance of components:
##                           PC1    PC2    PC3     PC4    PC5     PC6     PC7
## Standard deviation     2.1919 1.2311 1.0818 0.91629 0.8231 0.75905 0.62994
## Proportion of Variance 0.4368 0.1378 0.1064 0.07633 0.0616 0.05238 0.03607
## Cumulative Proportion  0.4368 0.5745 0.6809 0.75725 0.8188 0.87123 0.90730
##                            PC8     PC9    PC10    PC11
## Standard deviation     0.60780 0.58914 0.44280 0.32721
## Proportion of Variance 0.03358 0.03155 0.01782 0.00973
## Cumulative Proportion  0.94089 0.97244 0.99027 1.00000
```

```
screeplot(KAP.pca_know,type="lines",col=3)
```

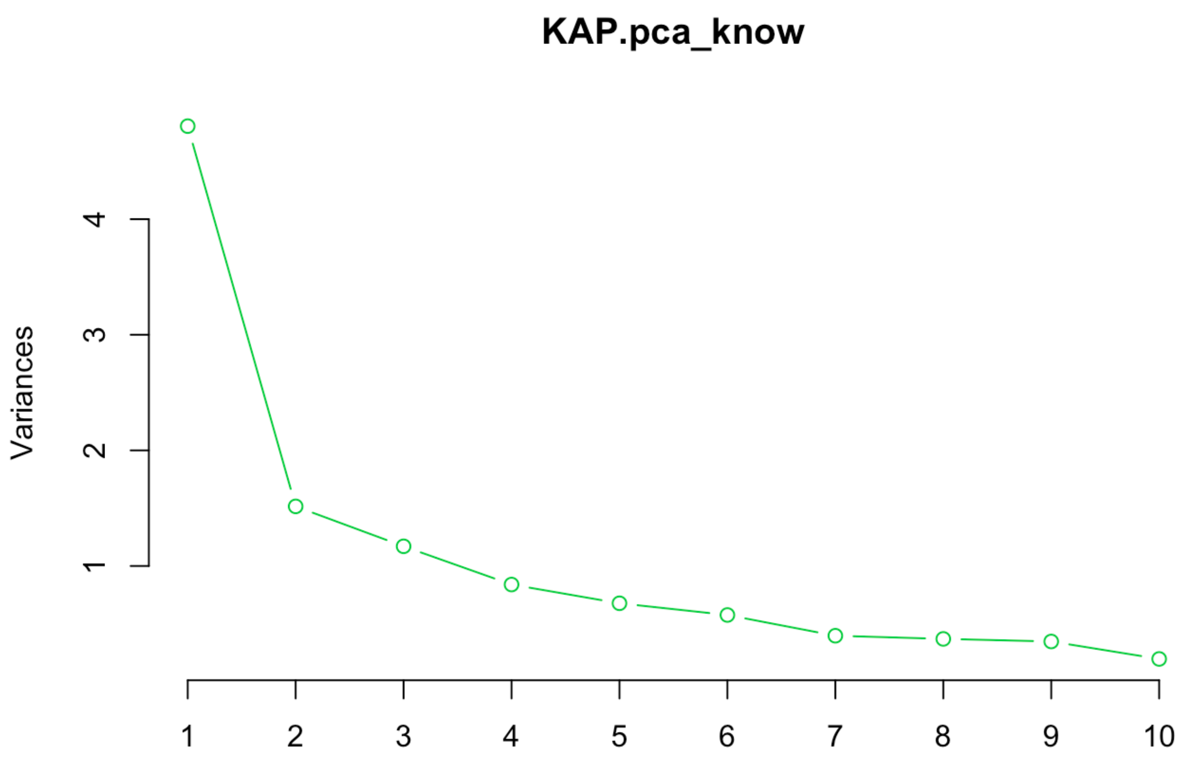

Logistic regression model

The code below assumes that you have run the t.test and univariable regression on all the variables to identify which one can be included in the model. Below we run a logistic regression and extract the odds ratios. We also evaluate the model fit using the HL test as well as the AUC.

```
PESTICIDE_DB$N31IF_REF = relevel(PESTICIDE_DB$N31IF, ref= "windy")
mylogit<-glm(knowlege_binary ~  N53PROXI+N31IF_REF+N36DO +N47DO , data = PESTI
CIDE_DB, family = "binomial")
summary((mylogit))
```

```
##
## Call:
## glm(formula = knowlege_binary ~ N53PROXI + N31IF_REF + N36DO +
##     N47DO, family = "binomial", data = PESTICIDE_DB)
##
## Deviance Residuals:
##     Min       1Q   Median       3Q      Max
## -2.3343  -1.0744   0.5719   0.8987   1.6408
##
## Coefficients:
##                         Estimate Std. Error z value Pr(>|z|)
## (Intercept)              0.63108    0.85198   0.741   0.4589
## N53PROXI>10metres(far)  -0.81406    0.38827  -2.097   0.0360 *
## N31IF_REFNOt-sure        0.22873    0.87726   0.261   0.7943
## N31IF_REFrainy           0.06698    0.59828   0.112   0.9109
## N31IF_REFvery-sunny&dry  1.22817    0.62434   1.967   0.0492 *
## N36DOYES                 0.79747    0.50304   1.585   0.1129
## N47DOYES                -0.92875    0.50322  -1.846   0.0649 .
## ---
## Signif. codes:  0 '***' 0.001 '**' 0.01 '*' 0.05 '.' 0.1 ' ' 1
##
## (Dispersion parameter for binomial family taken to be 1)
##
##     Null deviance: 215.58  on 161  degrees of freedom
## Residual deviance: 193.40  on 155  degrees of freedom
##   (5 observations deleted due to missingness)
## AIC: 207.4
##
## Number of Fisher Scoring iterations: 4
```

```
exp(cbind(Odds_and_OR=coef(mylogit), confint(mylogit)))
```

```
##                         Odds_and_OR     2.5 %     97.5 %
## (Intercept)               1.8796467 0.3543791 10.3622633
## N53PROXI>10metres(far)    0.4430542 0.2021419  0.9327032
## N31IF_REFNOt-sure         1.2570000 0.2263677  7.4151561
## N31IF_REFrainy            1.0692755 0.3283295  3.5380834
## N31IF_REFvery-sunny&dry   3.4149579 1.0049232 11.9752099
## N36DOYES                  2.2199212 0.8422995  6.1597782
## N47DOYES                  0.3950468 0.1380482  1.0152292
```

```
PESTICIDE_DB$knowlege_binary<- as.numeric(PESTICIDE_DB$knowlege_binary)
mylogit_l<- lrm(knowlege_binary ~ N53PROXI+N31IF+N36DO +N47DO , data = PESTICI
DE_DB, method = "lrm.fit" ,
                model = T, x = T, y = T,
                linear.predictors = T, se.fit = F)

mylogit_l
```

```
##
## Logistic Regression Model
##
## lrm(formula = knowlege_binary ~ N53PROXI + N31IF + N36DO + N47DO,
##     data = PESTICIDE_DB, method = "lrm.fit", model = T, x = T,
##     y = T, linear.predictors = T, se.fit = F)
## Frequencies of Missing Values Due to Each Variable
## knowlege_binary        N53PROXI           N31IF           N36DO
##               0               1               5               1
##           N47DO
##               1
##
##
##                        Model Likelihood     Discrimination    Rank Discrim.
##                           Ratio Test           Indexes           Indexes
## Obs            162    LR chi2     22.18     R2       0.174    C       0.708
##  0              62    d.f.            6     g        0.930    Dxy     0.415
##  1             100    Pr(> chi2) 0.0011     gr       2.534    gamma   0.464
## max |deriv| 2e-09                           gp       0.197    tau-a   0.197
##                                             Brier    0.204
##
##                           Coef    S.E.   Wald Z Pr(>|Z|)
## Intercept                  0.8598 0.8980  0.96  0.3383
## N53PROXI=>10metres(far) -0.8141 0.3883 -2.10  0.0360
## N31IF=rainy             -0.1617 0.7247 -0.22  0.8234
## N31IF=very-sunny&dry     0.9994 0.7671  1.30  0.1926
## N31IF=windy             -0.2287 0.8773 -0.26  0.7943
## N36DO=YES                0.7975 0.5030  1.59  0.1129
## N47DO=YES               -0.9288 0.5032 -1.85  0.0649
```

```
# goodness of fit evaluation
residuals(mylogit_l,type = "gof")
```

```
## Sum of squared errors     Expected value|H0                    SD
##            33.1023999            33.2423542             0.2012340
##                     Z                     P
##            -0.6954802             0.4867544
```

```
prob <- predict(mylogit, newdata=PESTICIDE_DB, type="response")
pred <- prediction(prob, PESTICIDE_DB$knowlege_binary)
perf <- performance(pred, measure = "tpr", x.measure = "fpr")
plot(perf)
```

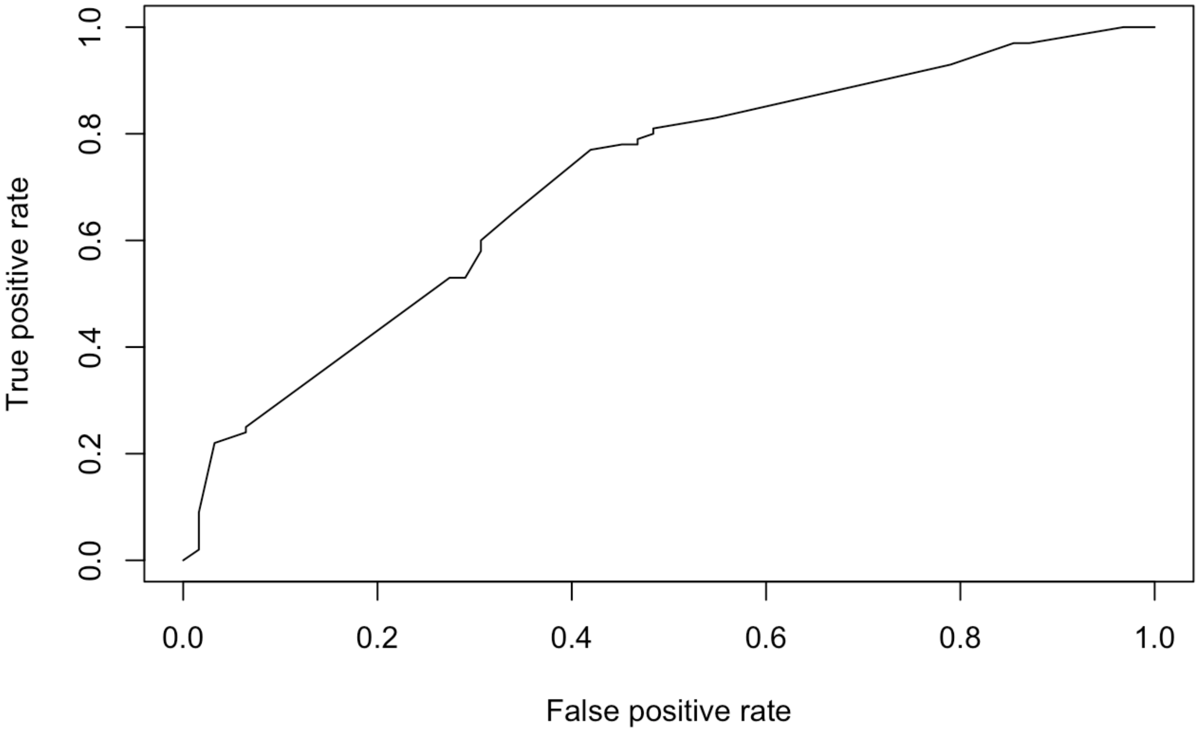


```
auc <- performance(pred, measure = "auc")
auc <- auc@y.values[[1]]
auc
```

```
## [1] 0.7075
```
