# Supplementary material for: A Framework for Integrating Qualitative and Quantitative Data in Knowledge, Attitude, and Practice Studies: A Case Study of Pesticide Usage in Eastern Uganda
Source: Front Public Health. 2017 Dec 8;5:318. doi: 10.3389/fpubh.2017.00318 (PMC5727069; doi:10.3389/fpubh.2017.00318)
Supplement: Supplementary file 1 [file Data_Sheet_1.zip › supplementary Material/Supplementary S3.pdf]

Supplementary material

A framework for integrating qualitative and quantitative data in knowledge attitude and practice studies: A case study of pesticide usage in eastern Uganda

Muleme James[1, 3,*], Kankya Clovice[1], John C Ssempebwa[3], Stella Mazeri[2], Adrian Muwonge[2]

Figure S1: Shows the variation in the KAP linear relationship for populations with different levels of knowledge

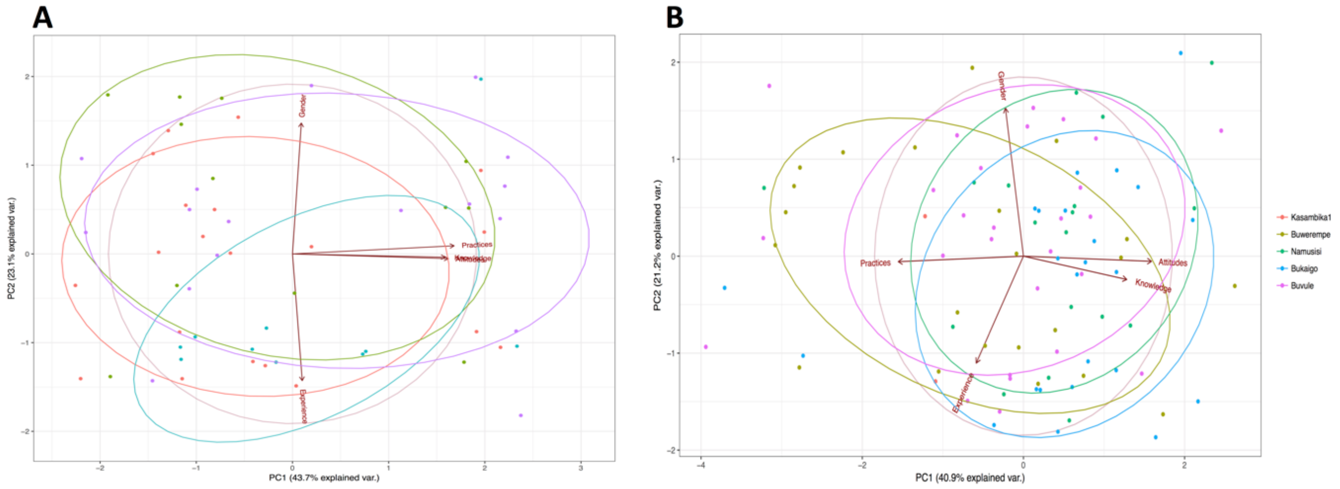

Panel A shows the principal component analysis for a population with low scores on the knowledge metric (<50%) we observe that the KAP linear relationship holds for this group. **Panel B** on the other hand is the principal component analysis for the population with high scores on the knowledge metric (>50%) and the KAP linear relationship does not hold for this group.

Figure S2. The relationship between practice and attitude and how it compares with Van Doorn's theory on the Practice-Attitude axis

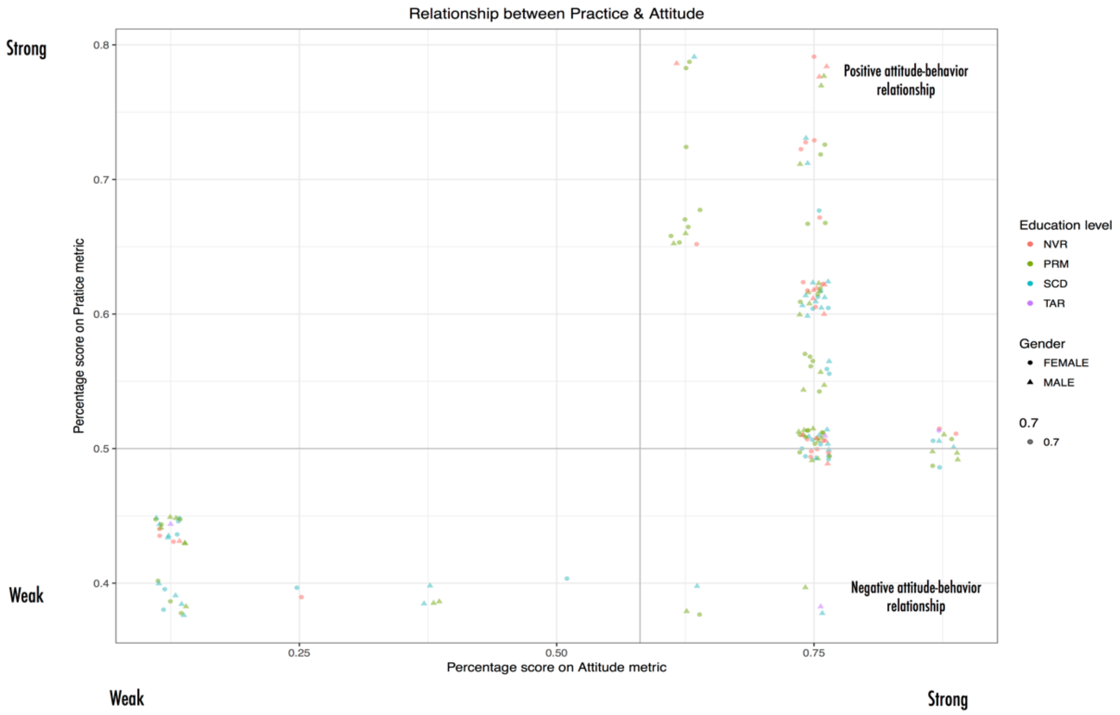

The figure shows the distribution of all the interviewed individuals in this study spread along the Practice metric on the y axis and the attitude metric on the x axis. The labelling weak – strong and the relationships in the two quadrants on the right are adopted from van Doorn J et al 2007. The figure shows that our data agrees with the authors theoretical postulate on the Practice-Attitude axis. The Education legende, NVR= never had formal education, PRM= Primary education, SCD= Secondary education and TAR= Tartiary education
